# Intrinsically Mitochondria‐Targeting Nanozyme via Coordination‐Assembly of Natural Quercetin for Cascade Antioxidant Therapy of Cerebral Ischemia‐Reperfusion Injury

**DOI:** 10.1002/advs.76038

**Published:** 2026-06-11

**Authors:** Wenxuan Zheng, Zhicheng Wang, Xin Zhou, Shuya Wang, Xiaojing Shi, Tingli Xiong, Ruishi Li, Yuting Lin, Zhen Chen, Jiawen Wei, Fei Li, Jinwen Ge, Kelong Ai, Chong Liu, Guiming Deng

**Affiliations:** ^1^ The Second Affiliated Hospital of Hunan University of Chinese Medicine Changsha China; ^2^ Xiangya School of Pharmaceutical Sciences Central South University Changsha China; ^3^ The First Affiliated Hospital of Hunan University of Chinese Medicine Changsha China; ^4^ Chongqing Jiulongpo Traditional Chinese Medicine Hospital Chongqing China; ^5^ Hunan Academy of Chinese Medicine Changsha China; ^6^ Hunan Provincial Key Laboratory of Cardiovascular Research Xiangya School of Pharmaceutical Sciences Central South University Changsha China

**Keywords:** cerebral ischemia/reperfusion injury, cGAS‐STING pathway, mitochondria‐targeting nanozymes, mtDNA, neuroinflammation

## Abstract

Mitochondrial dysfunction, culminating in oxidative stress‐driven release of mitochondrial DNA (mtDNA) and subsequent inflammatory activation, constitutes a central pathogenic axis in cerebral ischemia‐reperfusion injury. Disrupting this axis requires precise antioxidant delivery to neuronal mitochondria, a major therapeutic hurdle. Here, we uncover that the natural flavonoid quercetin (Quer) possesses an intrinsic ability to bind mitochondrial outer membrane proteins, revealing its unexploited potential as a natural mitochondrial‐targeting ligand. Leveraging this discovery, we engineered an ultrasmall mitochondria‐targeting cascade nanozyme through coordination‐driven self‐assembly of the natural flavonoid Quer with Fe^3+^. MCN currently generates Fe^2+^/Fe^3+^ dual‐valence centers that confer potent, superoxide dismutase‐catalase cascade catalytic enzyme activities. We further confirmed that the MCN traverse the compromised blood–brain barrier, localize within the ischemic brain, and are selectively delivered to neuronal mitochondria in a rodent stroke model. Through its cascade elimination of key ROS, MCN stabilizes mitochondrial function and prevents mtDNA leakage. By blocking the released mtDNA from activating the cGAS‐STING pathway in microglia, MCN reprograms the neuroinflammatory microenvironment and robustly attenuates brain injury, leading to significant functional recovery. This work establishes a paradigm of transforming inherent bioactivity of natural products into targeted catalytic nanomedicines, offering a precise therapeutic strategy for mitochondrial‐centric diseases.

## Graphical abstract

This study uncovers that quercetin naturally targets mitochondria. By coordinating quercetin with Fe^3+^, we engineer an ultrasmall cascade nanozyme (MCN) with superoxide dismutase‐catalase activities. MCN crosses the damaged blood–brain barrier, scavenges mitochondrial ROS, prevents mitochondrial DNA leakage, and blocks the cGAS‐STING pathway, thereby alleviating cerebral ischemia‐reperfusion injury in rats, offering a new paradigm for converting natural products into precision nanomedicines.

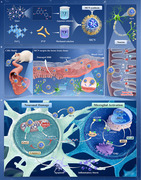

## 1 Introduction

Globally, ischemic stroke (IS) ranks among the foremost contributors to both mortality and enduring neurological impairment [1]. Currently, achieving vascular recanalization through intravenous thrombolysis or endovascular mechanical thrombectomy represents the gold‐standard therapy for restoring cerebral perfusion and salvaging neurons within the peri‐infarct region [2, 3]. However, this reperfusion process initiates multiple intricate and interlinked detrimental events, which paradoxically exacerbate cerebral tissue injury—termed cerebral ischemia‐reperfusion injury (CIRI). The presence of CIRI severely limits the ultimate efficacy of reperfusion therapy, making it a critical pathological factor contributing to poor neurological recovery and even secondary deterioration in patients [4]. The pathological progression of CIRI is intricate, involving a vicious cycle of multiple interconnected processes such as oxidative stress, mitochondrial dysfunction, endoplasmic reticulum (ER) stress, neuroinflammation, and apoptosis [5, 6, 7]. Among these, the uncontrolled burst of reactive oxygen species (ROS) in the early phase of reperfusion is widely recognized as the initiating factor that drives this vicious cycle [8]. Excessive ROS first directly attacks mitochondria, damaging the integrity of their inner membrane, resulting in the dissipation of the mitochondrial membrane potential (MMP), uncoupling of oxidative phosphorylation, and failure of ATP synthesis. More critically, ROS‐induced increases in mitochondrial membrane permeability promote the massive leakage of cytochrome c (Cyt c) and mitochondrial DNA (mtDNA) toward the cytoplasm. The leaked mtDNA, acting as a potent damage‐associated molecular pattern, drives microglial polarization toward a pro‐inflammatory M1 phenotype, accompanied by robust secretion of inflammatory mediators, resulting in a severe neuroinflammatory storm [9, 10, 11]. This inflammatory storm both directly destroys neurons and further intensifies oxidative stress alongside mitochondrial injury, creating a self‐perpetuating vicious cycle centered on ROS‐mitochondrial damage‐mtDNA leakage‐neuroinflammation. Ultimately, this cascade leads to irreversible neuronal apoptosis and cerebral infarction. Therefore, timely and efficient clearance of excessive ROS within neuronal mitochondria during the early stage of CIRI, thereby protecting mitochondrial integrity and blocking mtDNA leakage at its source, is considered one of the most promising strategies for disrupting this vicious cycle and achieving multi‐target synergistic neuroprotection.

However, achieving this strategy faces significant challenges. The core difficulty lies in how to precisely deliver therapeutic agents to the “bullseye”—the neuronal mitochondria within the injured brain region. Although mitochondrial targeting techniques (e.g., using lipophilic cations like triphenylphosphonium) have been explored, these positively charged targeting moieties often suffer from poor stability in vivo, rapid clearance, and difficulty in effectively crossing the blood–brain barrier (BBB), even in its pathologically compromised yet still selective state [12, 13]. Furthermore, many exogenous antioxidants, like superoxide dismutase (SOD) and catalase (CAT), themselves suffer from poor stability, short half‐lives, an inability to penetrate cell membranes, and a lack of mitochondrial localization capability [14]. Therefore, developing a novel therapeutic agent that combines excellent BBB penetration, efficient mitochondrial targeting, and wide‐ranging, sustained ROS elimination capability is key to overcoming the current therapeutic impasse in CIRI.

**SCHEME 1:**
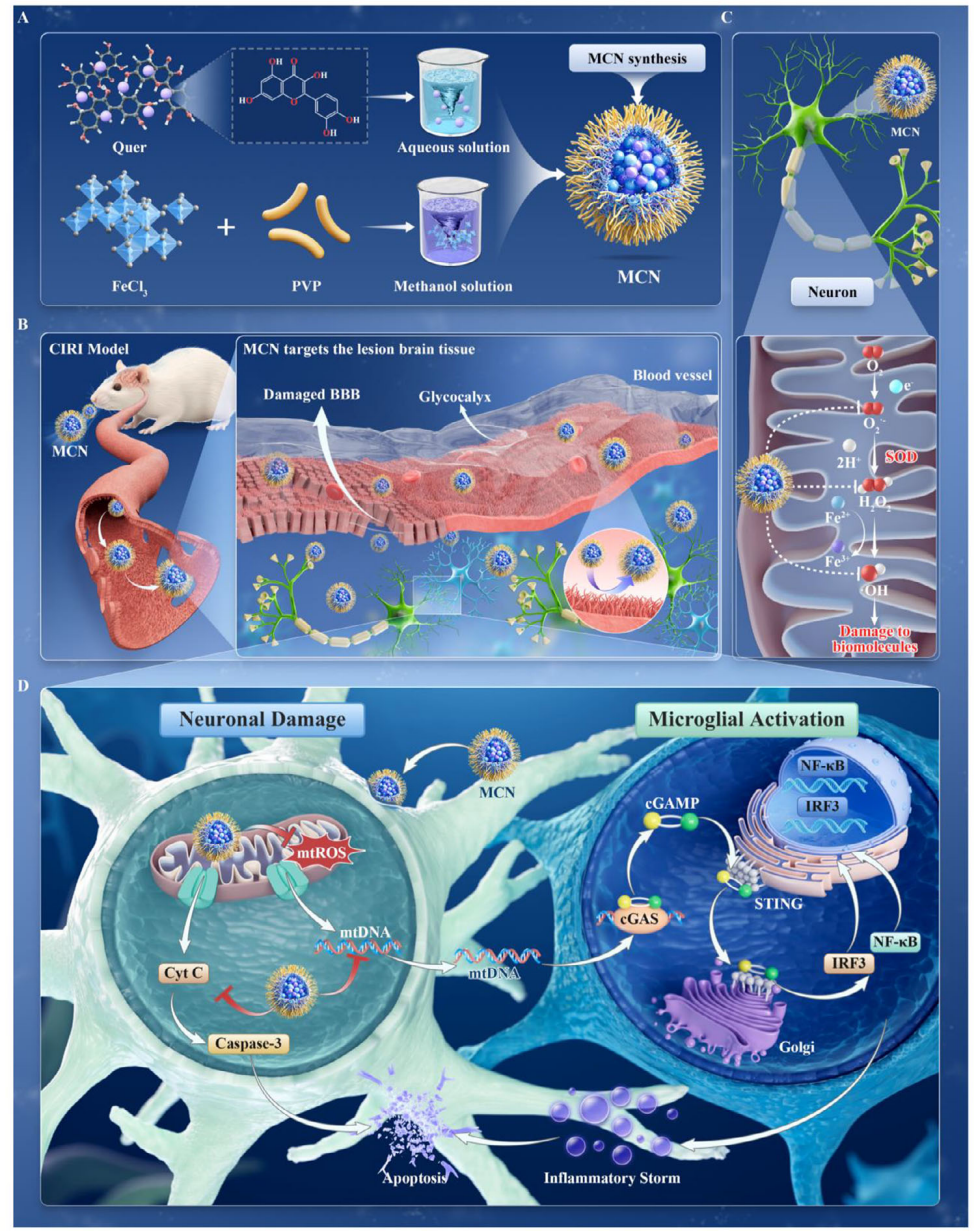
Schematic of MCN's therapeutic action in CIRI. (A) Illustrated scheme of MCN synthesis: MCNs are nano‐drugs obtained through the coordination reaction of Quer with Fe^3+^ at room temperature, where polyvinylpyrrolidone (PVP) is simultaneously introduced to regulate the growth and aggregation process of the nanoparticles. (B,C) MCN achieve efficient enrichment and antioxidant mechanisms in CIRI lesion tissues through a dual mechanism: they can traverse the compromised endothelial gaps of the BBB and exhibit high affinity for exposed matrix proteins (such as collagen III/IV) on injured cerebrovascular surfaces (B). Furthermore, by leveraging their Fe^2+^/Fe^3+^ dynamic dual‐valence centers, MCN enable “full‐course interception” of O_2_^·−^, H_2_O_2_, and ·OH (C). (D) The mitochondria‐targeting MCN protect mitochondrial functional integrity by scavenging mtROS, stabilizing the MMP, and maintaining ATP production, thereby reducing the leakage of mtDNA. The released mtDNA is detected by microglia, activating the cGAS‐STING signaling pathway, which in turn promotes the activation of nuclear factor kappa B (NF‐κB) and interferon regulatory factor 3 (IRF3). This drives the release of pro‐inflammatory factors and triggers an inflammatory storm, ultimately leading to neuronal apoptosis. By reducing mtDNA leakage at the source, MCN effectively suppresses the overactivation of the cGAS‐STING pathway, downregulates neuroinflammation, and alleviates ER stress. Ultimately, they inhibit Caspase 3‐mediated neuronal apoptosis, achieving multi‐pronged synergistic therapeutic effects against CIRI.

To address this challenge, we turned our attention to of quercetin (Quer), a flavonoid compound abundant in nature with rich pharmacological activities. Quer's exceptional antioxidant and anti‐inflammatory properties have been extensively documented [15]. Theoretically, Quer is an ideal candidate molecule for intervening in the aforementioned vicious cycle of CIRI. However, its inherent drawbacks—extremely poor water solubility, low oral bioavailability, instability under physiological conditions, and inability to autonomously cross the BBB—severely limit its direct application for treating central nervous system diseases [16]. Intriguingly, in this study, we made a breakthrough discovery: the Quer molecule itself exhibits exceptionally high binding affinity (with binding energies all below −4.8 kcal/mol) for several key proteins on the mitochondrial outer membrane. This computational prediction reveals that Quer is not merely a passive cargo needing to be carried; its own chemical structure harbors an intrinsic mitochondrial targeting code. This finding provides a revolutionary perspective and a solid theoretical foundation for directly developing Quer into an intelligent drug‐building unit that combines therapeutic activity with targeting functionality. Building on this insight, this study innovatively proposed a strategy of active ligand self‐assembly for in situ functionalization, successfully constructing mitochondria‐targeting cascade nanozymes (MCN). We ingeniously introduced ferric ions (Fe^3+^), which, through coordination‐driven self‐assembly with the ortho‐phenolic hydroxyl groups of Quer, enabled the one‐pot synthesis of ultra‐small (∼2.77 nm) nanocomplexes at room temperature [17]. This process achieved three key objectives simultaneously. First, the coordination interaction completely disrupted the crystalline structure of Quer, forming an amorphous metal‐polyphenol coordination network. This transformed Quer from being nearly water‐insoluble into highly water‐dispersible nanoparticles, perfectly solving its solubility issue (Scheme 1A). Second, during the coordination process, Quer acted as a reductant, partially reducing Fe^3+^ in situ to Fe^2+^, thereby forming a dynamic Fe^2+^/Fe^3+^ mixed‐valence catalytic center. This center endowed MCN with multiple nanozyme activities mimicking SOD and CAT, enabling the efficient, catalytic scavenging of the entire ROS cascade from superoxide anions (O_2_^.−^) to hydrogen peroxide (H_2_O_2_) and further to hydroxyl radicals (·OH). Their antioxidant efficiency far exceeds that of the pure Quer molecule. Finally, and most importantly, this self‐assembly process maximally preserved the surface chemical properties of the Quer molecule and the key functional groups responsible for its interaction with mitochondrial membrane proteins. Consequently, MCN fully inherited the intrinsic mitochondrial targeting capability of Quer.

Benefiting from their ultra‐small size of ∼2.77 nm, MCN can effectively traverse the gaps formed between endothelial cells of the compromised BBB following CIRI. Simultaneously, their surface‐rich phenolic hydroxyl groups confer high affinity for collagen exposed on the damaged vascular basement membrane, achieving specific enrichment in the ischemic brain region [18]. After entering the brain parenchyma, MCN are efficiently taken up by injured neurons and, relying on their inherent targeting property, precisely localize to mitochondria (Scheme 1B). Within mitochondria—the very source of ROS production and the epicenter of damage—MCN exert their multiple nanozyme activities, performing in situ efficient scavenging functions to rapidly eliminate excessive mitochondrial reactive oxygen species (mtROS). This fundamental intervention triggers a cascade of protective effects: the MMP is stabilized, ATP synthesis resumes, and mitochondrial morphology and functional integrity are preserved (Scheme 1C). The most direct and critical outcome is the inhibition of mitochondrial permeability transition, resulting in markedly diminished release of pro‐inflammatory mtDNA from mitochondria into the cytosol. Consequently, the activation of the mtDNA‐triggered cyclic GMP‐AMP synthase (cGAS)‐stimulator of interferon genes (STING) signaling pathway in microglia is effectively blocked. This promotes the polarization of microglia from the pro‐inflammatory M1 phenotype toward the anti‐inflammatory M2 phenotype, resulting in decreased release of inflammatory cytokines and increased expression of anti‐inflammatory factors, thereby quelling the neuroinflammatory storm. Concurrently, the improved mitochondrial function alleviates the closely coupled ER stress, further blocking the transmission of apoptotic signals (Scheme 1D). Ultimately, through this series of interlocking protective mechanisms, MCN effectively disrupts the vicious cycle of ROS‐mitochondrial damage‐inflammation‐apoptosis in CIRI. Significant neuroprotection is observed in both cellular and animal models following treatment with these agents, markedly reducing cerebral infarct volume, improving neurological deficits, and exhibiting excellent biocompatibility. In this work, we for the first time revealed and utilized the natural mitochondrial targeting property of Quer, establishing a novel integrated therapeutic paradigm of “activity itself is targeting”. Furthermore, by transforming active small molecules directly into functionally integrated, intelligent nanomedicine via coordination‐driven self‐assembly, we provide a novel design concept and a transferable paradigm for developing next‐generation synergistic therapeutic platforms targeting stroke and other complex pathological processes.

## 2 Results

### 2.1 Physicochemical Profiling of MCN

**FIGURE 1:**
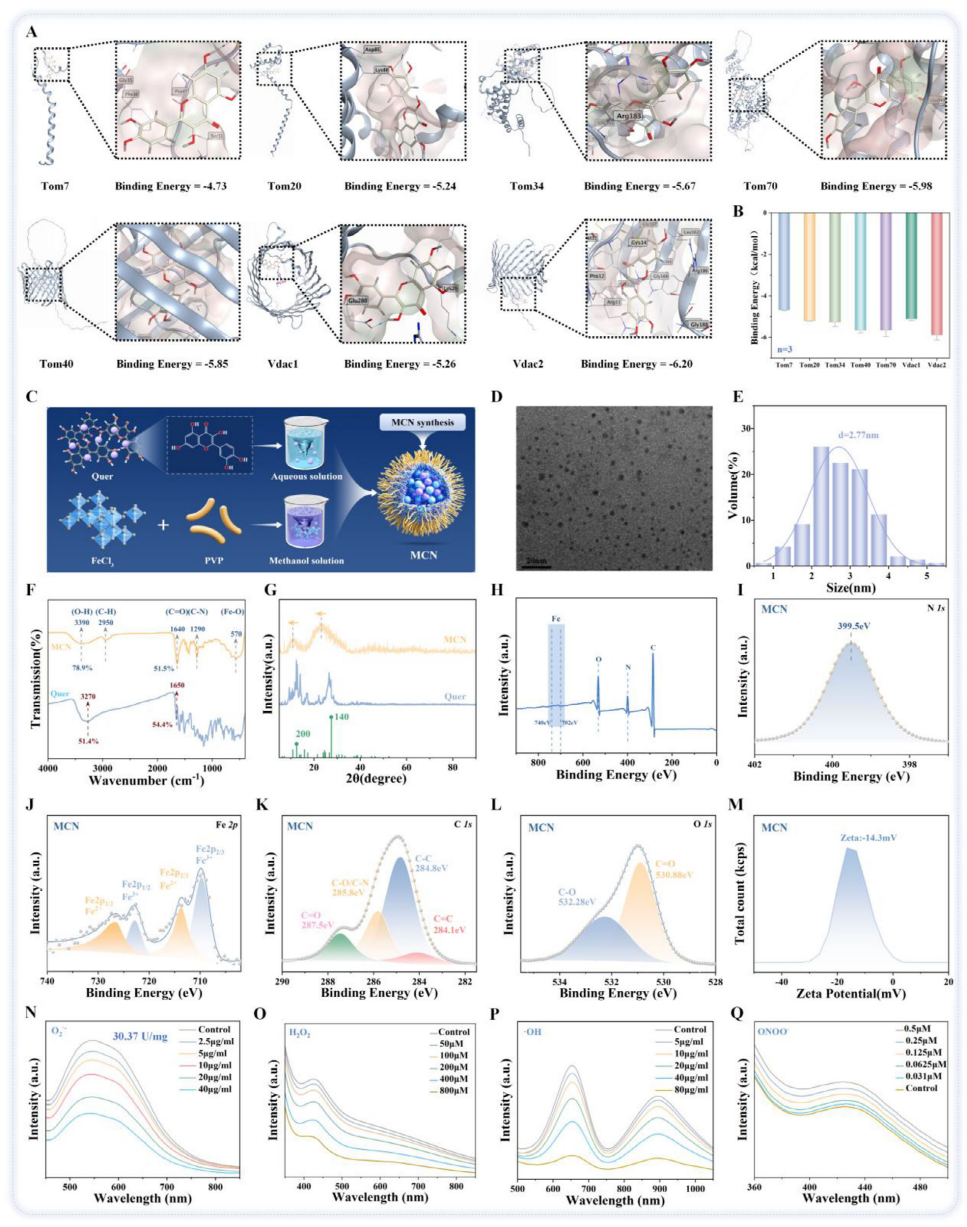
Physicochemical profiling of MCN. (A) Quer targets mitochondria: Illustrated scheme of molecular docking of Quer with the mitochondrial outer membrane proteins TOM7, TOM20, TOM34, TOM40, TOM70, Vdac1, and Vdac2. (B) Statistical chart of molecular docking binding energies. (C) Illustrated scheme of MCN synthesis. (D) High‐resolution TEM of MCN. Accelerating voltage: 200 kV; Scale bar: 20 nm. (E) Hydrodynamic diameter of MCN. (F) FTIR of MCN and Quer. (G) XRD patterns of MCN and Quer. (H) Total XPS spectrum for MCN. (I) Narrow‐scan XPS spectrum of N1s for MCN. (J) Narrow‐scan XPS spectrum of Fe2p for MCN. (K) Narrow‐scan XPS spectrum of C1s for MCN. (L) Narrow‐scan XPS spectrum of O1s for MCN. (M) Zeta potential diagram of MCN. (N) Generation and elimination activity of O_2_^.−^ in MCN. (O) Comparative assessment of the H_2_O_2_ elimination capacities of MCN. (P) Comparison of ·OH elimination capacities of MCN. (Q) Comparison of the ONOO^−^ elimination capacities of MCN.

To evaluate the mitochondrial targeting capability of Quer, molecular docking was carried out to examine the binding between Quer and a panel of mitochondrial outer‑membrane proteins, including Tom7, Tom20, Tom34, Tom40, Tom70, Vdac1, and Vdac2. The calculated binding energies were −4.73, −5.24, −5.67, −5.85, −5.98, −5.26, and −6.20 kcal/mol, respectively (binding energy < −4 kcal/mol is considered to indicate high affinity) (Figure 1A,B), demonstrating the promising mitochondrial‐targeting potential of MCN. As shown in Figure 1C, MCNs were prepared at room temperature via a coordination reaction among Quer, PVP, and Fe^3+^. In this system, Quer acts as a polyphenol ligand whose adjacent phenolic hydroxyl groups undergo Fe^3+^‐induced deprotonation, generating abundant negatively charged phenolate ions that subsequently coordinate with Fe^3+^ to form a stable coordination architecture. The introduction of PVP effectively regulates the growth and aggregation process of the MCN. The encapsulation efficiency (EE) of MCN was measured to be 82.6%, and the drug loading (DL) capacity was 31.6%. The high EE indicates that Quer was efficiently integrated into the coordination network structure, while the DL reflects the proportion of active ingredient in MCN. Notably, this DL is significantly higher than that of conventional inert nanocarriers (typically below 10%), fully demonstrating the advantage of the coordination‐driven self‐assembly strategy in achieving high drug loading—Quer not only serves as a therapeutic agent but also acts as a structural backbone participating in the formation of the coordination network, thereby avoiding the use of large amounts of inert carriers [19].

Transmission electron microscopy (TEM) imaging revealed that MCN exhibit uniform quasi‐spherical morphology with excellent monodispersity in aqueous solution, with a mean diameter of approximately 2.77 nm. (Figure 1D,E). Through Fourier‐transform infrared (FTIR) spectroscopy (Figure 1F), compared with free Quer, the absorption band of MCN spanning 1150–1200 cm^−1^, assigned to H─O─C stretching vibration, was significantly weakened, while a distinct band corresponding to the Fe─O coordination bond appeared at 571 cm^−1^, confirming successful coordination between Fe^3+^ and Quer and the formation of a meta‐polyphenol network. Moreover, a characteristic peak of PVP was observed at 1641 cm^−1^ in the MCN spectrum, indicating that PVP was successfully decorated on the MCN. X‐ray diffraction (XRD) was additionally utilized to examine the crystal structure of MCN (Figure 1G). The XRD pattern of free Quer showed sharp diffraction peaks at *2θ* ≈ 12.5° and 27.5°, which match well with the standard card (PDF#43‐1695) and can be indexed to crystal planes such as (200) and (140), indicating its well‐defined crystalline structure. In contrast, after forming MCN, these characteristic diffraction peaks of Quer completely disappeared, and only two broad halos were observed in the *2θ* range of 15°–30°. This distinct change confirms that coordination with iron ions completely disrupts the original crystalline structure of Quer, leading to the successful construction of an amorphous metal‐polyphenol coordination network. This structural feature constitutes an important physicochemical basis for the excellent water solubility and stability of MCN. X‐ray photoelectron spectroscopy (XPS) was employed to determine the elemental makeup and valence states of MCN. The XPS survey spectrum (Figure 1H) reveals that MCN contains C, O, N, and Fe. Additionally, a nitrogen signal typical of PVP is also observed, providing clear proof of the effective incorporation of Quer, PVP, and iron ions. The high‐resolution N 1s spectrum (Figure 1I) displays a symmetric single peak at 399.5 eV, corresponding to the nitrogen species of the amide group (─N─C═O) in PVP, confirming that PVP is modified on the MCN. The high‐resolution Fe 2p spectrum (Figure 1J) reveals the coexistence of Fe^2+^ and Fe^3+^ species, indicating that Quer acts as a reductant during the reaction and partially reduces Fe^3+^ to Fe^2+^. This mixed‐valence pair provides the chemical basis for MCN to achieve cyclic catalytic scavenging of diverse ROS. The C1s spectrum (Figure 1K) was resolved into four distinct peaks, assigned to C─C/C═C (284.8/284.1 eV) in the aromatic rings of Quer C─O/C─N (285.8 eV), and C═O in carbonyl/quinone groups (287.5 eV), confirming that MCN contains carbon structural features of both Quer and PVP. The O1s spectrum (Figure 1L) could be decomposed into two characteristic peaks at 530.88 eV (quinone C═O) and 532.28 eV (hydroxyl/ether C─O), indicating that the surface of MCN is rich in phenolic hydroxyl and quinone groups, which lays the foundation for its interaction via hydrogen bonding with exposed matrix proteins of injured cerebral vasculature and mitochondrial proteins. Zeta potential measurement showed that MCN carries a negative surface charge (−14.3 mV), resulting from the deprotonation of Quer during coordination, which is beneficial for achieving longer circulation time in the bloodstream (Figure 1M). Subsequently, we systematically evaluated the in vitro antioxidant performance of MCN. The nitroblue tetrazolium (NBT) assay showed that MCN possesses remarkable O_2_^.−^ scavenging ability, with an SOD‐mimetic activity of 30.37 U/mg (Figure 1N). Considering that H_2_O_2_ is a product of SOD catalysis, we further verified the H_2_O_2_‐scavenging capacity of MCN, and the results showed that MCN could effectively remove H_2_O_2_ in a concentration‐dependent manner via a CAT‐like catalytic reaction (Figure 1O). Crucially, this SOD‐CAT cascade catalytic property can efficiently eliminate ROS storms and reduce oxidative stress [20, 21]. The 3,3’,5,5’‐tetramethylbenzidine (TMB)‐based assay indicated that 80 µg/mL MCN could eliminate 79.21% of ·OH (Figure 1P). In addition, MCN also exhibited efficient scavenging ability toward the highly toxic ONOO^−^(Figure 1Q). These results collectively demonstrate that MCN possesses broad‐spectrum antioxidant functions and can cooperatively inhibit the ROS cascade.

To further evaluate the translational potential of MCN, their colloidal stability was assessed by incubating them in simulated physiological fluid (in PBS buffer at pH 7.4 with 10% FBS added) for 72 h. Dynamic light scattering (DLS) measurements revealed that the hydrodynamic size of MCN stayed between 2 and 4 nm over the entire incubation period. The polydispersity index (PDI) remained below 0.2, indicating excellent colloidal stability and no aggregation. Meanwhile, the absolute zeta potential value consistently remained above −10 mV, confirming that MCN possesses strong resistance to serum protein adsorption (Figure S1). Furthermore, the characteristic absorption peak of MCN at ∼350 nm retained approximately 70% of its initial intensity after 72 h, indicating slow degradation under physiological conditions. In sharp contrast, free Quer precipitated rapidly within 30 s after being added to the medium, and no effective signal could be detected by DLS (Figure S2). This fully highlights the superior stability imparted to MCN by the coordination‐driven self‐assembly strategy. We further investigated the catalytic stability of the Fe^2+^/Fe^3+^ dual‐valence centers in MCN using XPS. Before the catalytic reaction, the Fe 2p spectrum of MCN exhibited characteristic peaks of Fe^2+^ and Fe^3+^. Following an overnight reaction of MCN with H_2_O_2_ at 37°C, the Fe 2p spectrum remained largely unchanged, with no obvious shifts in binding energy, and the Fe^2+^/Fe^3+^ ratio remained largely unchanged (Figure S3). These results confirm that the Fe valence states in MCN remain stable during the catalytic cycle, providing a structural basis for their sustained SOD‐CAT cascade activity.

To quantitatively evaluate the catalytic efficiency of MCN, we determined its enzyme kinetic parameters by Michaelis–Menten fitting (Figure S4). The results showed that the SOD‐like activity had a *K*_m_ of 28.05 mm and a *V*_max_ of 34.2 U/mg; the CAT‐like activity had a *K*_m_ of 33.51 mm and a *V*_max_ of 126.99 U/mg. To further compare the stability of the nanozyme, we measured the O_2_^.−^ scavenging ability of the nanozyme and natural enzymes under different pH conditions (Figures S5 and S6). Notably, under acidic conditions simulating the ischemic core (pH 6) and penumbra (pH 6.5), the scavenging ability of MCN did not decrease significantly. A key limitation of natural SOD is its narrow pH tolerance. While natural SOD exhibits high catalytic activity at physiological pH 7.4, its activity is greatly reduced in the acidic environment (pH 6.5–6) faced by ischemic cerebral tissue. In contrast, MCN maintains robust SOD‐like activity at both pH 6.5 and 6. More importantly, the near‐complete inactivation of natural SOD under acidic conditions prevents the generation of its substrate H_2_O_2_, thereby rendering CAT non‐functional and ultimately causing failure of the entire endogenous antioxidant cascade. The inherent SOD‐CAT cascade capability of MCN enables them to continuously scavenge ROS even in acidic pathological environments, constituting a significant advantage over natural enzymes.

### 2.2 MCN Target Mitochondria in Neurons of CIRI Lesion

The BBB is a highly specialized functional barrier unit. Its core structure consists of brain capillary endothelial cells and the tight junctions between them, together with the underlying basement membrane. The periphery is enveloped by the end‐feet of astrocytes. It serves as a crucial barrier isolating the systemic circulation from the central nervous system and maintaining neural homeostasis [22]. However, while the BBB performs its normal physiological functions and protects the homeostasis of the brain microenvironment, it also constitutes a substantial hindrance to delivering drugs to the brain. Following the occurrence of CIRI, the BBB is compromised, with pericytes and astrocytes swelling and tight junctions between vascular endothelial cells disrupted, thereby allowing drugs with smaller particle sizes to pass through [23, 24]. Therefore, considering the presence of the BBB, whether MCN can specifically target the damaged areas of CIRI is crucial for ensuring their therapeutic efficacy. We established a rat CIRI model using the intraluminal filament occlusion method. Specifically, blood supply in the middle cerebral artery was blocked for 2 h to induce ischemia, followed by filament removal to restore blood flow for 24 h of reperfusion. Cerebral blood flow was monitored using a laser Doppler flowmeter during the procedure. During the ischemic phase, cerebral blood flow in the left hemisphere (ischemic side) was significantly decreased by 75.96% compared to the right hemisphere (contralateral control side), confirming successful blockage of the middle cerebral artery (Figure S7). The animal experimentation protocol was approved by the Ethics Committee of the Laboratory Animal Center, the First Hospital of Hunan University of Chinese Medicine (Ethics No.: 202404024).

In vitro experiments, we used mouse hippocampal neuronal cells (HT22 cells) as the research subject. A neuronal hypoxia/reoxygenation (H/R) model was successfully established by inducing cell hypoxia with cobalt chloride (CoCl_2_) for 16 h followed by 8 h of reoxygenation. To validate the reliability of the H/R model, we compared the cytotoxicity of CoCl_2_ treatment with that of the gold‐standard oxygen‐glucose deprivation/reoxygenation (OGD/R) model. HT22 cells were subjected to either OGD/R (glucose‐free medium, 95% N_2_/5% CO_2_, hypoxia for 4 h, followed by reoxygenation for 24 h) or treatment with various concentrations of CoCl_2_ (100, 200, and 400 µm) for 16 h, followed by reoxygenation for 8 h. Cell viability was evaluated by the Cell Counting Kit‐8 (CCK‐8) method, while live/dead cell staining was carried out with Calcein‐AM/PI. The results showed that OGD/R reduced cell viability to 58.38% of the control group. CoCl_2_ treatment decreased cell viability in a concentration‐dependent manner, and the viability in the 400 µm CoCl_2_ group was 56.22%, showing no statistically significant difference from the OGD/R group. Similarly, live/dead cell staining demonstrated the same trend. Consistently, live/dead cell staining revealed comparable proportions of PI‐positive (dead) cells in the OGD/R and 400 µm CoCl_2_ groups, whereas lower concentrations of CoCl_2_ caused only mild injury (Figure S8). These findings indicate that treatment with 400 µm CoCl_2_ for 16 h followed by 8 h of reoxygenation effectively recapitulates the level of cellular injury induced by OGD/R in HT22 cells. Numerous studies have validated the CoCl_2_‑induced H/R model as a reliable and reproducible in vitro model for cerebral ischemia‑reperfusion injury research [25, 26].

**FIGURE 2:**
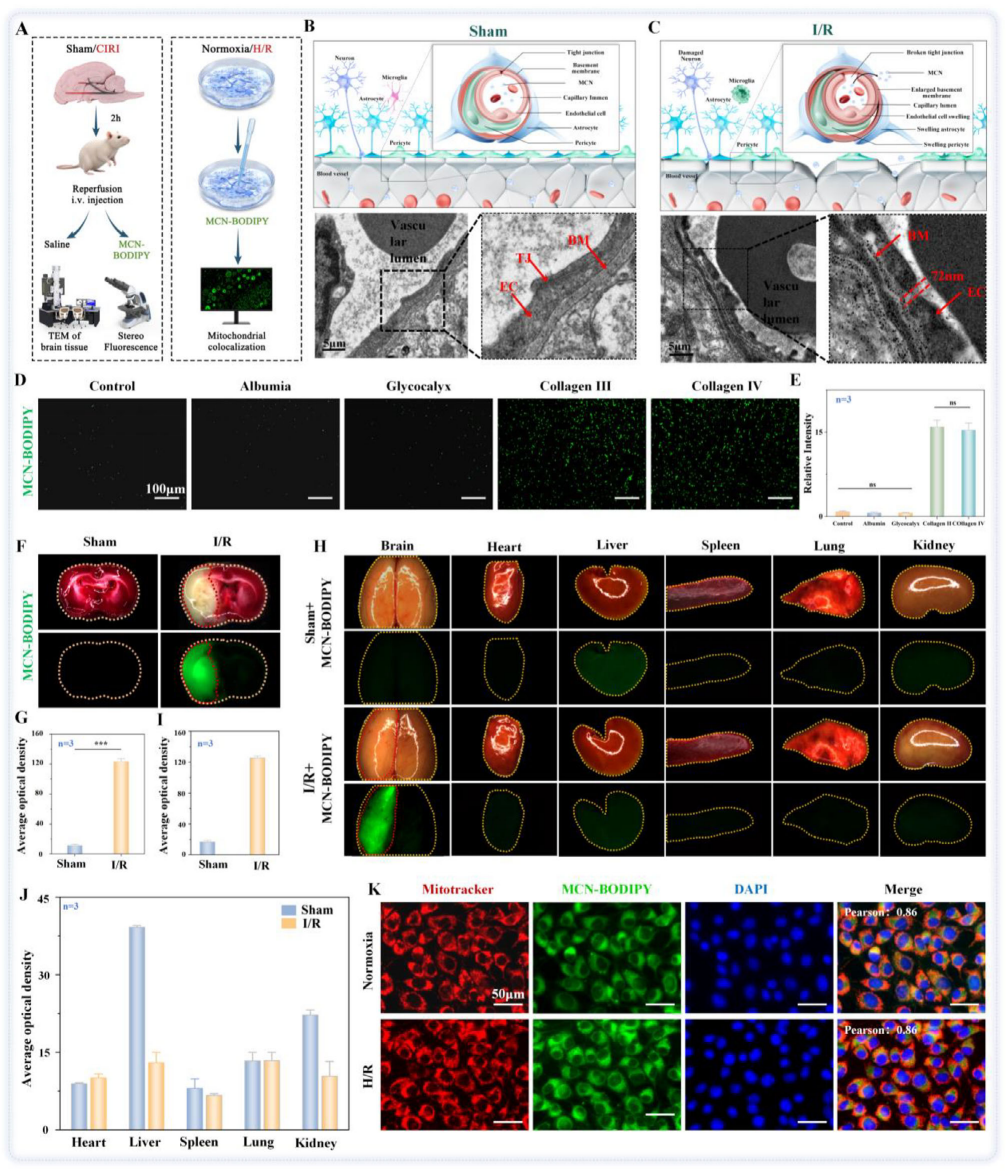
MCN specifically targeted damaged cerebral tissue and efficiently improved CIRI. (A) Illustrated scheme of the specific targeting of MCN to damaged cerebral tissue and hypoxic neurons assessed by stereofluorescence and cerebral tissue by TEM. (B) Illustrated scheme of the BBB and representative TEM images of capillaries in normal cerebral tissue. Accelerating voltage: 200 kV. (C) Schematic diagram of the BBB and representative TEM images of capillaries in I/R‐injured cerebral tissue. Scale bar: 5 µm. (D) Representative images of protein‐coated plate experiments were used to measure the binding affinity of MCN toward albumin, glycocalyx, collagen III, and collagen IV. Scale bar: 100 µm. (E) Quantitative analysis of the protein‐coated plate experiment. (F,G) TTC and stereofluorescence imaging of brain slices obtained from Sham and I/R group rats after sublingual intravenous administration of MCN‐BODIPY (F) and statistics of fluorescence intensity (G). (H–J) Representative images of fluorescence imaging (H) and statistics of fluorescence intensity (I,J) in major organs (brain, heart, liver, spleen, lung, and kidney) of rats in the I/R group 1 h after sublingual intravenous injection of MCN‐BODIPY. (K) Representative fluorescence images showing colocalization of MCN‐BODIPY with mitochondria in HT22 cells. Scale bar: 50 µm. All data are presented as the mean ± SE. For in vitro studies, three independent replicates were performed; for in vivo studies, each group consisted of three animals. Statistical significance was determined by one‐way ANOVA with Tukey post hoc test. ns: *P* > 0.05, ^***^P < 0.001.

This model was then co‐incubated with BODIPY‐labeled MCN (both BODIPY and MCN‐BODIPY exhibited fluorescence under both normal and ultraviolet (UV) light) to investigate the targeting and localization capability of MCN (Figure 2A; Figure S9). First, TEM observation revealed a clearly visible vascular basement membrane in the Sham‐operated group, with normal endothelial cell architecture and preserved tight junction complexes (Figure 2B). In contrast, following I/R, degradation of the basement membrane, swollen endothelial cells, and opened tight junctions forming gaps of approximately 72 nm were observed in the cerebral tissue. This gap size is significantly larger than the particle size of MCN (∼2.77 nm), providing the possibility for MCN to specifically target the damaged cerebral tissue by traversing the compromised endothelial gaps of the BBB (Figure 2C).

We further validated the penetration capability of MCN using an in vitro BBB model. This model was established using the most recognized and widely used human brain microvascular endothelial cell line (hCMEC/D3) in current BBB research [27]. First, the barrier integrity of the hCMEC/D3 monolayer was assessed via measurement of transendothelial electrical resistance (TEER) values. The results showed that the TEER value of endothelial cells in the Normoxia group was 99.01 Ω cm^2^, indicating intact tight junctions and robust barrier integrity. In contrast, after H/R, the TEER reading for endothelial cells significantly decreased to 41.82 Ω cm^2^, demonstrating that hypoxia/reoxygenation injury disrupted the tight junctions between endothelial cells, thereby creating conditions for MCN to infiltrate across the barrier. Subsequently, the apparent permeability coefficient (Papp) was determined by quantifying the fluorescence signal of MCN‑BODIPY in the lower chamber. The Papp value in the Normoxia group was extremely low, indicating that an intact BBB effectively impedes MCN penetration. In marked contrast, the Papp value in the H/R group rose to 53.46 times that of the Normoxia group (Figure S10). These results indicate that H/R treatment leads to disruption of endothelial tight junctions, thereby providing an opportunity for MCN to traverse the BBB. Next, we further investigated the interaction between MCN and the exposed matrix proteins (collagen III and IV) on damaged blood vessels, using the glycocalyx as a control. As shown in Figure 2D,E, by co‐incubating BODIPY‐labeled MCN with plates coated with albumin, glycocalyx, collagen III, or collagen IV, we observed that MCN‐BODIPY exhibited almost no affinity for albumin or glycocalyx but demonstrated high affinity for collagen III and collagen IV. To quantitatively evaluate the binding affinity of MCN to the basement membrane proteins collagen III and collagen IV exposed after vascular injury, we performed affinity analysis using surface plasmon resonance technology. Collagen III and collagen IV proteins were immobilized on the chip surface, and various concentrations of MCN solution (10–2560 nm) were used as the mobile phase to detect the interaction between them. The results showed that MCN specifically bound to both collagen III and IV, with equilibrium dissociation constant (KD) values of 5.41 and 25.10 nm, respectively, both in the nanomolar range (10^−9^–10^−8^ m), indicating that MCN possesses high binding affinity for both collagen proteins (Figure S11). These findings confirm that MCN can efficiently recognize and tightly bind to collagen III and IV exposed on the damaged vascular basement membrane, providing direct molecular evidence for their targeted enrichment in the lesioned area during CIRI. This is because the Quer‐iron coordination network within MCN can form strong hydrogen bonds with glutamate, methionine, arginine, lysine, and phenylalanine residues in collagen III and collagen IV via its abundant surface phenolic hydroxyl and carboxyl groups [18]. Based on the aforementioned results, we propose that MCN achieves efficient accumulation in CIRI‐lesioned cerebral tissue through a dual mechanism: the ability to traverse the damaged endothelial gaps of the BBB and a high affinity for exposed matrix proteins (collagen III/IV) on injured cerebral blood vessels. Subsequently, to verify the in vivo targeting distribution of MCN, we administered MCN‐BODIPY via sublingual venous injection to I/R group rats before reperfusion. First, combining 2,3,5‐triphenyltetrazolium chloride (TTC) staining with stereofluorescence imaging, we found that following intravenous injection, MCN‐BODIPY specifically accumulated only in the I/R‑damaged cerebral tissue, with almost no distribution observed in regions without infarctions. This indicates that the intact BBB hinders the entry of MCN‐BODIPY into cerebral tissue, while I/R injury promotes its specific targeting and enrichment, thereby achieving targeted therapy for CIRI with MCN (Figure 2F,G). Additionally, we simultaneously examined the distribution of MCN‐BODIPY in the major organs (brain, heart, liver, spleen, lungs, and kidneys) of rats at 1 h after sublingual venous injection. In the Sham, the drug fluorescence predominantly localized to the liver and kidneys, with almost no drug accumulation detected in the cerebral tissue, indicating that MCN can be metabolized and excreted via the liver and kidneys. In sharp contrast, a clear and specific targeting of MCN‐BODIPY to the infarcted cerebral tissue was observed in I/R rats (7.8‐fold that of the Sham‑operated group), providing direct evidence once again for the damaged cerebral tissue targeting capability of MCN (Figure 2H–J). The above results indicate that MCN possesses favorable brain‐targeting properties, providing a prerequisite for investigating their subsequent mechanisms of action. To further elucidate the in vivo pharmacokinetic behavior and tissue distribution dynamics of MCN, we extended the observation period to 24 h and performed stereoscopic fluorescence imaging at three time points (1, 6, and 24 h), as well as plasma concentration measurements at multiple time points (5 min, 15 min, 30 min, 1 h, 2 h, 4 h, 6 h, 8 h, 12 h, and 24 h) by detecting the fluorescence intensity of MCN‐BODIPY in plasma. In the Sham‐operated group, the fluorescence of MCN‐BODIPY was mainly enriched in the liver and kidneys, peaking at 1 h, markedly diminishing by 6 h, and almost disappearing by 24 h, suggesting that MCN is rapidly cleared primarily via the hepatic and renal routes. In the I/R, in addition to the liver and kidneys, obvious fluorescence was clearly detectable within cerebral tissue at 1 h, peaked at 6 h, and residual signals were still detectable at 24 h, whereas no significant fluorescence was observed in the contralateral normal cerebral tissue throughout the entire period (Figures S12 and S13). Plasma pharmacokinetic analysis revealed that the temporal profile of plasma levels of MCN‐BODIPY exhibited a typical biexponential decay, fitting a two‐compartment model. The distribution half‐life (*t*_1/2_α) was approximately 0.8 h, and the elimination half‐life (*t*_1/2_β) was approximately 11.1 h. The inflection point of the curve appeared at 4 h post‐administration, indicating that the drug primarily underwent rapid distribution before this time point, followed by a slow elimination phase. Notably, when the fluorescence in the ischemic cerebral tissue peaked at 6 h, the plasma drug concentration had already decreased to approximately 25% of its initial peak value after the early rapid distribution phase. By 24 h, the plasma drug concentration approached the lower limit of quantification, while a relatively strong fluorescence signal was still detectable in the brain (Figure S14). Together, these findings demonstrate that MCN can rapidly cross the damaged BBB and exhibit long‐term retention in ischemic cerebral tissue, with a distinct “lagged peak” characteristic of brain accumulation. This provides direct pharmacokinetic evidence supporting the therapeutic efficacy of MCN even when administered at delayed time points after reperfusion.

We employed MitoTracker to stain mitochondria for further evaluation of the mitochondrial targeting ability of MCN‐BODIPY in HT22 cells. As shown in Figure 2K, MCN exhibited high mitochondrial targeting under both Normoxia and H/R conditions, with Pearson's correlation coefficients reaching 0.86. This result indicates that H/R injury did not affect the mitochondrial targeting performance of MCN in HT22 cells.

We further validated the mitochondrial targeting capability of MCN using immunofluorescence colocalization in both cellular and animal models. In vitro, we examined the mitochondrial colocalization of MCN in primary neurons, BV2 cells, and CTX TNA2 cells. The results showed that MCN‐BODIPY exhibited high colocalization with Mitotracker in all these cell types, with Pearson correlation coefficients ≥ 0.84 (Figure S15 *)*. In vivo, after injecting MCN‐BODIPY into Sham and I/R model rats, cerebral tissues were collected for frozen sectioning and co‐stained with the neuronal marker NeuN and the mitochondrial marker TOM20. The staining results revealed that in the ischemic cortex, hippocampus, and striatum, the fluorescence signal of MCN‐BODIPY was highly colocalized with TOM20 in NeuN‐positive neurons, with Pearson correlation coefficients all above 0.85. In contrast, due to the intact BBB in the Sham, no green fluorescence of MCN‐BODIPY was observed in any of the three brain regions, further confirming the ability of MCN to go through the damaged BBB (Figure S16). Notably, in vitro experiments showed that MCN efficiently colocalized with mitochondria in multiple brain cell types, including primary neurons, BV2 microglia, and CTX TNA2 astrocytes (Pearson correlation coefficient ≥ 0.84), suggesting that MCN themselves possess broad‐spectrum mitochondrial targeting capability. However, in the in vivo CIRI model, the fluorescence signal of MCN‐BODIPY was mainly observed in NeuN‐positive neurons, while it was barely detectable in microglia or astrocytes. We attribute this discrepancy primarily to the unique pathological and anatomical environment in vivo. In the ischemic core and penumbra, neurons are the first and most directly exposed cell type to blood components after BBB disruption. Neuronal cell bodies and processes are broadly dispersed across the brain parenchyma, and many neurons are located in proximity to microvessel walls. Therefore, when the BBB is compromised, circulating MCN can extravasate directly from blood vessels and be rapidly taken up by adjacent neurons [28]. Furthermore, in vitro experiments involve static, uniform culture conditions where all cells are equally exposed to MCN. In contrast, the transport of MCN into the cerebral parenchyma through the damaged BBB in vivo is a dynamic, localized, and concentration‐gradient‐driven process, and neurons may exhibit higher uptake efficiency due to their high mitochondrial density and active metabolism. Thus, while MCN themselves possess broad‐spectrum mitochondrial targeting capability, in the complex pathological environment of in vivo cerebral ischemia‐reperfusion, neurons become the preferential target cells after MCN cross the damaged BBB due to their unique spatial localization and metabolic demands. This phenomenon does not negate the potential mitochondrial targeting of MCN toward glial cells but rather reflects the high spatiotemporal heterogeneity of in vivo drug distribution. To elucidate the molecular mechanism underlying the mitochondrial targeting of MCN, we knocked down TOM20 expression in HT22 cells using siRNA (knockdown efficiency of 75.6%) (Figure S17). After incubating the cells with MCN‐BODIPY, mitochondria were stained with MitoTracker. Relative to the negative control siNC group, the findings revealed that TOM20 knockdown significantly reduced the uptake of MCN‐BODIPY by HT22 cells, and the colocalization signal with MitoTracker was almost undetectable. This loss‐of‐function experiment demonstrated that knockdown of TOM20 alone substantially impaired the mitochondrial localization ability of MCN (Figure S18). In contrast, other mitochondrial outer membrane proteins, such as VDAC1, primarily function as metabolite channels and are not directly involved in protein import processes [29, 30]. Therefore, TOM20 plays an irreplaceable and critical role in the mitochondrial targeting of MCN, serving as the main receptor mediating the entry of MCN into mitochondria.

In summary, the collective evidence strongly demonstrates that MCN can exhibit selective homing to I/R‑damaged cerebral tissue and is rapidly taken up by damaged neurons, providing a foundation for the efficient and safe treatment of CIRI by MCN.

### 2.3 In Vivo Efficacy Evaluation of MCN and Mechanistic Analysis Based on Transcriptomics

**FIGURE 3:**
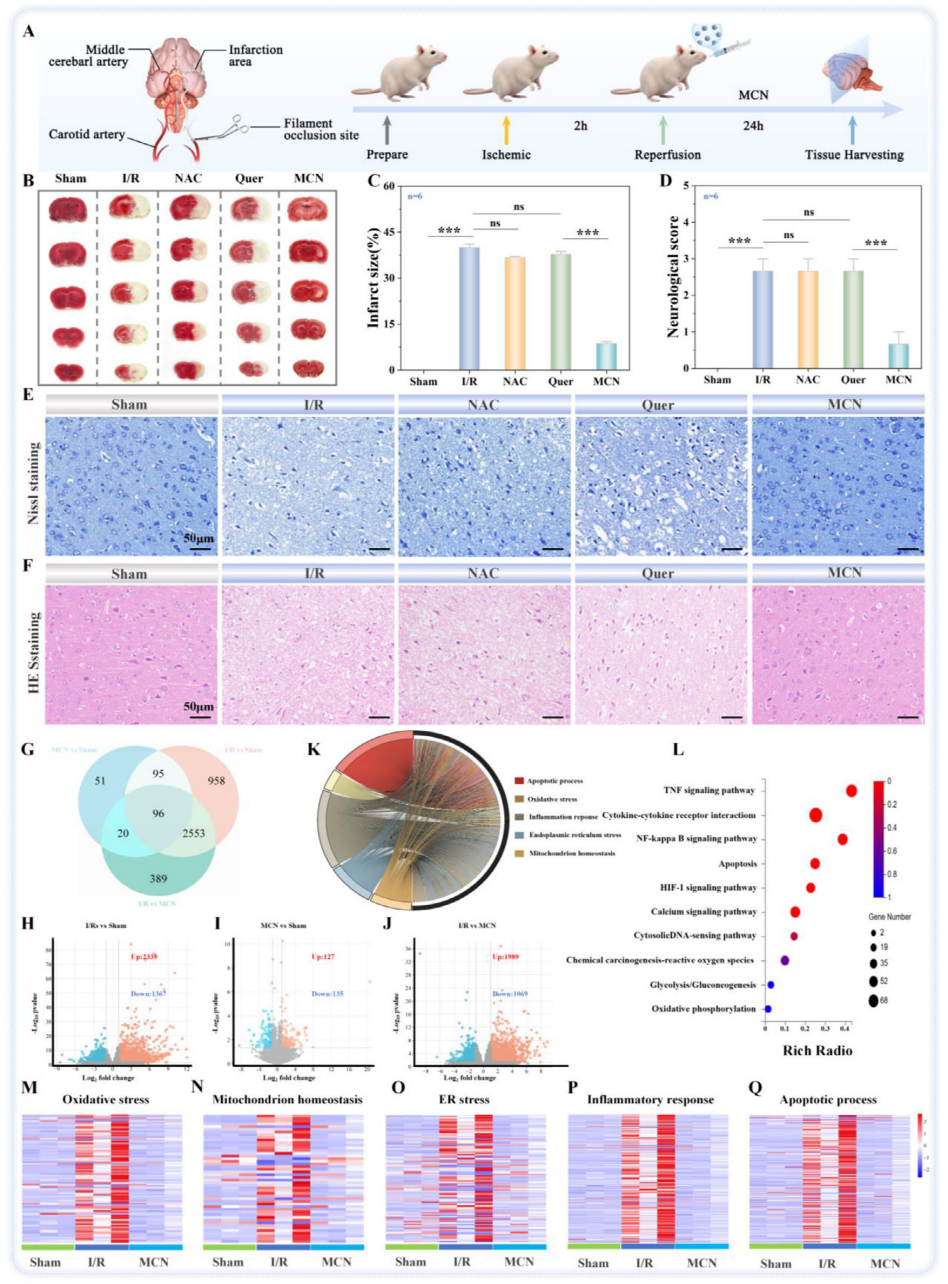
MCN relieves improved I/R. (A) Illustrated scheme of MCN therapeutic evaluation in CIRI. (B,C) Representative images of TTC‐stained brain sections (B) and quantitative analysis of infarct volume (C) across the various treatment groups. (D) Assessment of neurological deficits in rats across the various treatment groups. (E) Representative Nissl‐stained sections illustrating the influence of MCN on cerebral tissue morphology and neuronal integrity. Scale bar: 50 µm. (F) Representative HE‐stained sections of rat cerebral tissue from the various treatment groups. Scale bar: 50 µm. (G) Analysis using Venn/UpSet diagrams reveals the overlap of DEGs across different groups. (H–J) Differential gene volcano plots (|log_2_FC| ≥ 1 and *P* ≤ 0.05) for the Sham and I/R group (H), Sham and MCN group (I), I/R and MCN group (J). (K) GO enrichment analysis of biological processes involved in DEGs between the I/R group and the MCN‐treated group. (L) KEGG pathway enrichment analysis of DEGs in the MCN‐treated group versus the I/R group. (M–Q) Heatmap of DEGs in selected GO biological processes, including oxidative stress (M), mitochondrion homeostasis (N), ER stress (O), inflammation response (P), apoptotic process (Q). All data are presented as the mean ± SE. For (B,C), each group consisted of six animals; for (E–Q), each group consisted of three animals. Statistical significance was determined by one‐way ANOVA and Tukey's post hoc test: ns: *P* > 0.05, ^***^ *P* < 0.001.

To comprehensively assess the treatment efficacy of MCN on cerebral tissue in CIRI, we established a CIRI rat model using the embolization method. Following 2 h of ischemia, drugs or an equivalent volume of saline were administered via sublingual venous injection. After 24 h of reperfusion, we examined the therapeutic efficacy of MCN against I/R injury in vivo (Figure 3A). First, we used TTC staining results and neurological function scores as efficacy criteria to screen for the optimal dosing regimen. As shown in Figures S19–S21, TTC staining showed that the infarct area occupied 37.88% of the total brain area in the I/R group. Notably, even at 4 mg/kg, MCN demonstrated a significant ameliorative effect on the cerebral infarct area in I/R rats, which undoubtedly highlights its promising therapeutic potential for CIRI. It is noteworthy that the treatment effectiveness of MCN against CIRI exhibited a clear dose‐dependent trend over a defined dosage window. At 8 mg/kg, MCN nearly entirely reversed the infarct area in the I/R‐lesioned cerebral tissue and significantly improved neurological function scores. The infarct percentage decreased from 37.88% to 7.80%, and the neurological score also dropped from 3–4 points to an average of 0–1 points. Given that the efficacy of MCN at 12 mg/kg showed no significant difference compared to that at 8 mg/kg, we ultimately selected 8 mg/kg as the optimal dose for MCN in treating CIRI and proceeded with subsequent in‐depth studies using this dose. Simultaneously, we used equivalent doses of Quer and the clinically approved antioxidant drug N‐acetylcysteine (NAC) as controls to further evaluate the therapeutic effect of MCN. As shown in Figure 3B,C, large cerebral infarcts were observed in the I/R group, confirming the successful establishment of the rat CIRI model. Compared to the I/R group, the cerebral infarct area in the MCN group was almost completely reversed, indicating that MCN effectively ameliorates CIRI. However, because of their absence of brain‐targeting capability, the antioxidant drug NAC and Quer at equivalent doses did not show significant improvement in the cerebral infarct area of I/R rats. Neurological function scores were used to statistically assess neurological damage in rat cerebral tissue after different drug treatments. The findings showed that the I/R group had the most profound neurological impairment, with scores of 3–4 points. In contrast, neurological scores in the MCN treatment group significantly decreased to only 0–1 points, almost identical to the Sham‑operated group. In comparison, NAC and Quer at equivalent doses showed no significant therapeutic effect (Figure 3D). This result aligns with the trend observed in the TTC staining, indicating that 8 mg/kg of MCN can significantly improve I/R‐induced neurological functional impairment, with efficacy markedly superior to that of NAC and Quer. To more intuitively observe the pathological changes in cerebral tissue and the extent of neuronal injury, HE and Nissl staining were employed to examine the histological organization of the cerebral tissue (Figure 3E,F). Relative to the Sham‐operated group, the cortical region of the I/R group showed shrunken or even lysed neuronal cells, with numerous vacuoles and tissue necrosis visible between cells, and Nissl bodies were significantly atrophied. After MCN treatment, vacuoles were markedly reduced, the cerebral tissue structure of I/R was significantly improved, and the count and appearance of Nissl bodies returned to values approaching those observed in the Sham‐operated group. Similarly, consistent with the TTC staining and neurological function scoring results, NAC and Quer did not show significant recovery of the tissue organization of I/R‐damaged cerebral tissue.

We next evaluated the therapeutic effect of MCN regarding the sustained neurological impairments in I/R‑subjected animals. First, the modified neurological severity score (mNSS) was used to assess locomotor activity, sensory responses, reflexes, and equilibrium in every experimental group. I/R group exhibited severe sensorimotor impairment, showing a mean mNSS value of around 13. Remarkably, the neurological impairments in the MCN‐treated group significantly improved over time, and their mNSS approached the values observed in the Sham‐operated group by day 14 post‐surgery. In agreement with the in vivo treatment outcomes, no obvious neurological improvement was observed in the NAC or Quer groups (Figure S22). Next, we conducted the adhesive removal test to further assess tactile sensitivity and sensorimotor performance. Compared with the Sham‐operated group, the I/R group required substantially more time to both contact and peel off the adhesive tape, reflecting profound sensorimotor deficits. In the MCN‐treated group, the touch/removal time gradually shortened over time after surgery and approached the Sham‐operated group level by day 14, whereas no significant improvement was observed in the NAC or Quer groups (Figures S23 and S24). The corner test, which reflects sensorimotor asymmetry caused by unilateral dopaminergic system damage, showed that the proportion of right turns was significantly reduced in the I/R group. This phenomenon was markedly ameliorated by MCN treatment, while no significant changes were observed in the NAC or Quer groups (Figure S25). Furthermore, administration of MCN markedly extended the lifespan of I/R‑subjected rats. Rats in the I/R and NAC groups did not survive beyond 9 days, and rats in the Quer group did not survive beyond 10 days, whereas the proportion of surviving rats in the MCN‑treated group rose markedly (Figure S26).

To further define the optimal treatment window for MCN for CIRI, we administered a single intravenous injection of MCN (8 mg/kg) at different time points after reperfusion (0, 2, 4, 6, and 8 h). At 24 h post‐surgery, cerebral infarct size was quantified using TTC staining, and neurological function was evaluated using the Longa score. TTC analysis revealed that the cerebral infarct area was 8.64% in the 0 h post‐reperfusion group and 9.55% in the 2 h group, both markedly reduced relative to the I/R model group (40.99%), indicating that MCN exerted a significant protective effect within this time window. The infarct area was 20.21% in the 4 h group and 27.28% in the 6 h group, still exhibiting a certain degree of protection, with the 4 h group showing better efficacy than the 6 h group. In the 8 h group, the infarct area was 39.76%, close to that of the I/R (40.99%), suggesting that MCN had largely lost its efficacy by this time point (Figures S27 and S28). The Longa score results were consistent with the TTC staining trend, further validating the above observations (Figure S29). These observations offer experimental support for the application of MCN in delayed reperfusion therapy for clinical IS patients.

Collectively, these data strongly indicate that MCN can significantly ameliorate CIRI, and its therapeutic efficacy is markedly superior to that of existing antioxidant drugs, NAC and the Quer, at equivalent doses. Because NAC and Quer failed to exert therapeutic benefits in the experiments described above, they were not carried forward for more detailed analysis in later studies.

Based on the significant neuroprotective effects exhibited by MCN in the in vivo CIRI model, we performed RNA sequencing (RNA‐Seq) on the Sham, I/R, and MCN groups to investigate the underlying therapeutic mechanisms. First, we examined the overlap of differentially expressed genes (DEGs) between groups using VENN/UpSetR plots. Figure 3G reveals that 3702 DEGs were detected when comparing the Sham and I/R groups, which validates the successful generation of the CIRI model and demonstrates that I/R injury profoundly alters gene expression in cerebral tissue. Notably, while 3058 DEGs existed between the MCN and I/R groups, only 262 DEGs were found between the MCN and Sham‐operated groups, restoring the dysregulated gene expression caused by I/R injury. Second, we generated volcano plots using thresholds of |log_2_FC| ≥ 1 and *P*‐value ≤ 0.05. Compared to the Sham‐operated group, the I/R group showed 2335 upregulated and 1367 downregulated genes. In contrast, compared to the I/R group, the MCN group exhibited 1989 upregulated and 1069 downregulated genes, while compared to the Sham‐operated group, the MCN group had only 127 upregulated and 135 downregulated genes. These results further confirm the significant ameliorating effect of MCN on CIRI‐related gene expression (Figure 3H–J). To further explore the molecular basis underlying the protective effects of MCN against CIRI, we performed gene ontology (GO) analysis on the DEGs between the I/R group and the MCN‐treated group. As shown in Figure 3K, these DEGs were primarily involved in biological processes such as the apoptotic process, inflammatory response, mitochondrial homeostasis, response to oxidative stress, and response to ER stress. Heatmap cluster analysis of the relevant genes according to these five core pathways revealed that the gene expression pattern of the I/R group was significantly different from that of both the Sham and MCN‐treated groups. In contrast, the expression pattern of the MCN‐treated group was highly similar to that of the Sham‐operated group, suggesting that MCN likely exert their therapeutic effects by regulating these related genes (Figure 3M–Q). Further Kyoto Encyclopedia of Genes and Genomes (KEGG) pathway analysis showed that the DEGs in the MCN‐treated group were mainly enriched in inflammation‐related signaling pathways (such as the cytosolic DNA‐sensing pathway, TNF signaling pathway, and NF‐kappa B signaling pathway), mitochondrial damage‐related pathways (such as cytokine‐cytokine receptor interaction and protein processing in the ER), as well as apoptosis and HIF‐1 signaling pathways (Figure 3L).

To gain further insight into the molecular mechanisms underlying the protective effects of MCN, we extracted a set of key differentially expressed genes from our transcriptomic dataset, focusing on those associated with oxidative stress (Sod2, Cybb, Cyba), mitophagy (Park2), mitochondrial dynamics (Tspo), and the electron transport chain (Ak2, Cox4i2, Polg2, and Atp7a). A heatmap of these genes (Figure S30) shows that, compared with the Sham‐operated group, the expression of Sod2, Cybb, Cyba, Ak2, Tspo, Cox4i2, Polg2, and Atp7a was significantly increased in the I/R group, while Park2 expression was decreased. MCN treatment substantially reversed these changes, bringing the expression levels back to near‑Sham values. These results suggest that MCN may protect mitochondrial function and exert its neuroprotective effects at least in part through regulating these mitochondrial‑related targets.

Based on these findings, we hypothesize that MCN may achieve therapeutic effects against CIRI by alleviating ER stress and mitochondrial damage, thereby ameliorating oxidative stress and inflammatory responses in cerebral tissue, and subsequently inhibiting apoptosis. Subsequent experiments were designed to validate the therapeutic mechanisms of MCN suggested by this transcriptomic analysis.

### 2.4 MCN Eliminates Mitochondrial Damage

**FIGURE 4:**
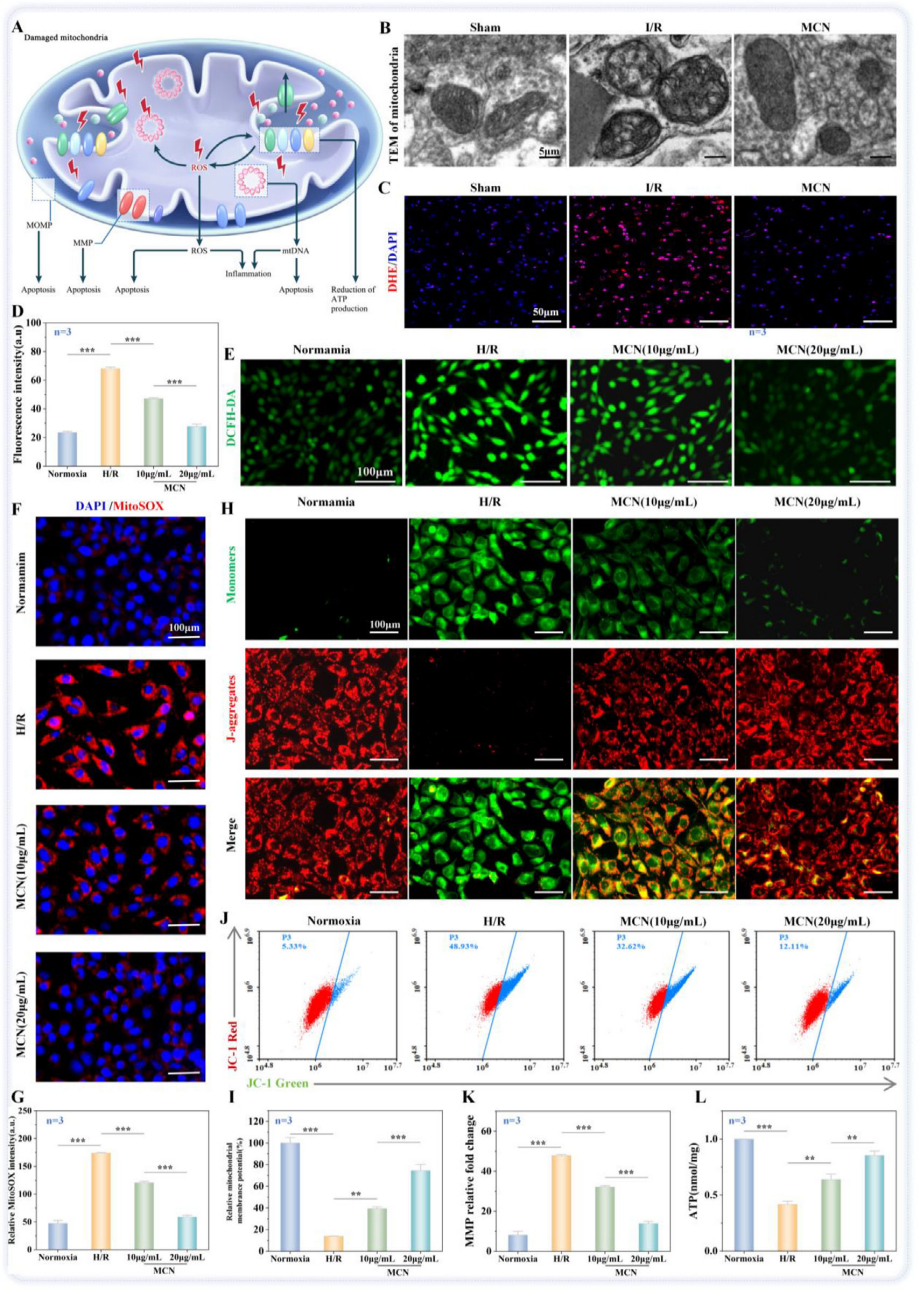
MCN eliminates mitochondrial damage. (A) Illustrated scheme of damaged mitochondria. (B) TEM micrographs showing neuronal mitochondrial morphology. Accelerating voltage: 200 kV; Scale bar 5 µm. (C) Representative images depicting ROS levels in cerebral tissue across all experimental groups. Scale bar 50 µm. (D,E) Quantification of ROS staining intensity (D) and representative fluorescence micrographs (E) in HT22 cells across the various treatment groups. Scale bar: 100 µm. (F,G) Representative fluorescence micrographs (F) and quantification (G) of MitoSOX staining in HT22 cells across the various treatment groups. Scale bar: 100 µm. (H,I) Representative fluorescence micrographs of JC‐1 staining (H) and quantification (I) in HT22 cells across the various treatment groups. Scale bar: 100 µm. (J,K) Flow cytometric analysis of MMP (J) in HT22 cells and quantification analysis (K) of the relative mean fluorescence intensity. (L) The ATP content in HT22 cells across the various treatment groups. All data are presented as the mean ± SE. For in vitro studies, three independent replicates were performed; for in vivo studies, each group consisted of three animals. Statistical significance was determined by one‐way ANOVA with Tukey post hoc test. ^**^ *P* < 0.01, ^***^ *P* < 0.001.

Mitochondria serve as the cell's energy hubs and constitute the main source of ROS production within neurons. During CIRI, the electron transport chain (ETC) on the inner mitochondrial membrane fails to transfer electrons properly, leading to a decrease in the MMP [31, 32]. This not only results in the release of mtDNA, contributing to neuroinflammation, but also impairs ATP synthesis, exacerbating cellular damage (Figure 4A). Thus, it is critically important to examine how MCN protects neuronal mitochondria. TEM was employed to visualize the impact of MCN on neuronal mitochondrial integrity during CIRI. As shown in Figure 4B, Relative to the Sham‐operated group, mitochondria within I/R neurons exhibited pronounced injury, including swelling, disruption of cristae, compromised membrane integrity, and vacuole formation. In contrast, the morphology of neuronal mitochondria in the MCN group was restored to a state comparable to that observed in the Sham‐operated group, clearly demonstrating that MCN can effectively alleviate mitochondrial damage. Subsequently, we investigated the antioxidative effect of MCN in cerebral tissue. ROS in cerebral tissue sections was labeled using the dihydroethidium (DHE) probe. ROS levels in the damaged cerebral tissue of the I/R group rose to 2.15 times those in the Sham‐operated group, suggesting that I/R challenge triggered a robust surge of ROS within the injured brain region. Notably, after treatment with MCN, the ROS level in the lesioned cerebral tissue significantly decreased to only 1.09‐fold of the Sham‐operated group (Figure 4C; Figure S31). In parallel, the effect of MCN on improving H/R‐induced mitochondrial damage in HT22 cells was further investigated in vitro. First, cell viability assessed by the CCK‐8 assay determined that the optimal dose of MCN for ameliorating H/R‐induced HT22 cell damage was 20 µg/mL, and its efficacy was dose‐dependent (Figure S32). Subsequently, we selected the optimal dose of 20 µg/mL and half the optimal dose (10 µg/mL) to further investigate the improvement of mitochondrial damage in HT22 cells by MCN. DCFH‐DA and MitoSOX probes were used to detect the scavenging effects of MCN on H/R‐induced ROS and mtROS in HT22 cells, respectively. Exposure to H/R markedly elevated ROS and mtROS levels in HT22 cells, reaching 2.9 times and 3.65 times those in the Normoxia group, respectively. Treatment with MCN effectively eliminated these excessively produced ROS. At the 10 µg/mL dose, cellular ROS and mtROS declined to 2.01 and 2.53 times the levels measured in the Normoxia group, respectively. At the higher concentration of 20 µg/mL, both ROS and mtROS nearly returned to baseline values (1.19‐ and 1.24‐times the Normoxia levels, respectively) (Figure 4D–G).

To further quantitatively evaluate the overall clearance efficiency of MCN toward intracellular ROS, we detected ROS levels in HT22 cells from different treatment groups using flow cytometry. As shown in Figure S33, in the H/R group, the proportion of ROS‑positive cells rose to 45.96%, markedly exceeding the value observed in the Normoxia group (5.92%). Following exposure to MCN at concentrations of 10 and 20 µg/mL, this proportion fell dose‑dependently to 17.96% and 4.73%, respectively, providing additional evidence for the potent ROS‑eliminating capacity of MCN. To verify whether the therapeutic effect of MCN depends on mitochondria‐targeted mtROS scavenging, we knocked down TOM20 expression in HT22 cells using siRNA and detected mtROS levels and live/dead cell staining in each group. In the negative control siNC group, H/R treatment led to a significant increase in mtROS level (3.52‐fold higher than that of the Normoxia+siNC group) and a substantial increase in cell death rate. After MCN (20 µg/mL) treatment, the mtROS level decreased to 1.27‐fold that of the Normoxia + siNC group, and live/dead cell staining showed that the proportion of dead cells was significantly reduced to a level similar to that of the Normoxia + siNC group. However, in the TOM20 knockdown group (siTOM20), H/R treatment also induced mtROS elevation and cell death, but MCN failed to effectively reduce mtROS (which remained at 2.72‐fold that of the Normoxia + siTOM20 group), and live/dead cell staining showed that the protective effect of MCN against cell death was almost completely lost. Furthermore, compared with the Normoxia + siNC group, the Normoxia + siTOM20 group showed no significant differences in mtROS levels or cell viability, indicating that TOM20 knockdown itself does not directly affect the basal state of the cells (Figures S34 and S35). These results demonstrate that TOM20 is a key receptor for MCN to enter mitochondria and exert its antioxidant and cytoprotective effects. Knockdown of TOM20 prevents MCN from targeting mitochondria to scavenge ROS, thereby greatly diminishing the protective capability of MCN, confirming that the mitochondrial targeting of MCN is essential for its function.

Collectively, these findings clearly indicate that MCN can effectively scavenge the abnormally produced peroxides in nerve cells under hypoxic conditions. As mentioned earlier, mitochondria rely on the membrane potential gradient generated by the ETC to maintain normal ATP synthesis, and the preservation of MMP integrity is essential for both oxidative phosphorylation and ATP synthesis within mitochondria.

JC‐1, a fluorescent indicator for monitoring MMP, builds up in the mitochondrial matrix of healthy cells and generates polymeric J‐aggregates that display red fluorescence. When the MMP is too low, JC‐1 primarily exists in the monomeric form, displaying green fluorescence. Therefore, we represented changes in MMP using the fluorescence intensity ratio of aggregates to monomers. As shown in Figure 4H,I, after H/R, the MMP of HT22 cells decreased from 100% to 13.85%, indicating severely impaired neuronal mitochondrial function under H/R stimulation. The MCN treatment groups significantly increased the MMP in H/R‐injured HT22 cells in a dose‐dependent manner: at the 10 µg/mL dose, MMP increased to 39.39%, and at the 20 µg/mL dose, the MMP percentage could be raised to 74.79%, demonstrating that MCN can effectively restore MMP. We also assessed the MMP level in HT22 cells using flow cytometry. The results similarly showed that MCN effectively improved the MMP level in H/R‐injured HT22 cells, with efficacy being dose‐dependent (Figure 4J,K). Subsequently, ATP generation was used to further elucidate mitochondrial function. As shown in Figure 4L, MCN effectively reversed the H/R‐induced reduction in ATP production in HT22 cells. At the 10 µg/mL dose, ATP production increased from 0.42‐fold to 0.64‐fold of the Normoxia group; at the 20 µg/mL dose, it increased to 0.85‐fold of the Normoxia group. As shown in Figures S36 and S37, to further verify the protective effect of MCN on mitochondria at the molecular and functional levels, we first measured the oxygen consumption rate (OCR) and extracellular acidification rate (ECAR) of neurons. The results showed that basal respiration, ATP‐linked respiration, and maximal respiratory capacity were significantly lower in the I/R group than in the Sham‐operated group, whereas MCN treatment significantly restored these OCR parameters. Meanwhile, ECAR results revealed that the glycolysis level was compensatorily elevated in the I/R group and returned to near‐normal levels after MCN treatment, indicating that MCN effectively restores mitochondrial respiratory function and metabolic homeostasis.

We next performed Quantitative Real‐time polymerase chain reaction (qPCR) to evaluate the transcript levels of mitochondrial function‑related and antioxidant enzyme genes. In vivo, relative to the Sham‐operated group, the I/R group exhibited a marked downregulation of the ATP synthase subunit (*ATP5tb*) mRNA to 0.43 times the control level, while the mRNA of the pro‑apoptotic factor cytochrome c (*cycs*) was significantly upregulated to 2.81 times the Sham value. Following MCN treatment, ATP5tb expression was largely restored, and cycs expression was effectively suppressed. Moreover, the mRNA abundance of the antioxidant enzymes *SOD2* and *GPx1* in the I/R group fell to 0.41 and 0.37 times the Sham‐operated group levels, respectively. MCN administration markedly elevated the expression of both genes, bringing them back to 0.88 and 0.85 times the Sham values, respectively. Comparable results were obtained from in vitro experiments (Figures S38 and S39). These results confirm at the transcriptional level that MCN not only restores mitochondrial energy metabolism and inhibits apoptotic pathways but also enhances the endogenous antioxidant defense capacity of neurons, which is highly consistent with the OCR/ECAR and TEM observations described above.

The above evidence indicates that MCN can target mitochondria, ameliorate the oxidative stress state of mitochondria under hypoxia, and maintain normal ATP levels by stabilizing the MMP, thereby improving neuronal mitochondrial damage.

### 2.5 MCN Inhibit Neuroinflammation

**FIGURE 5:**
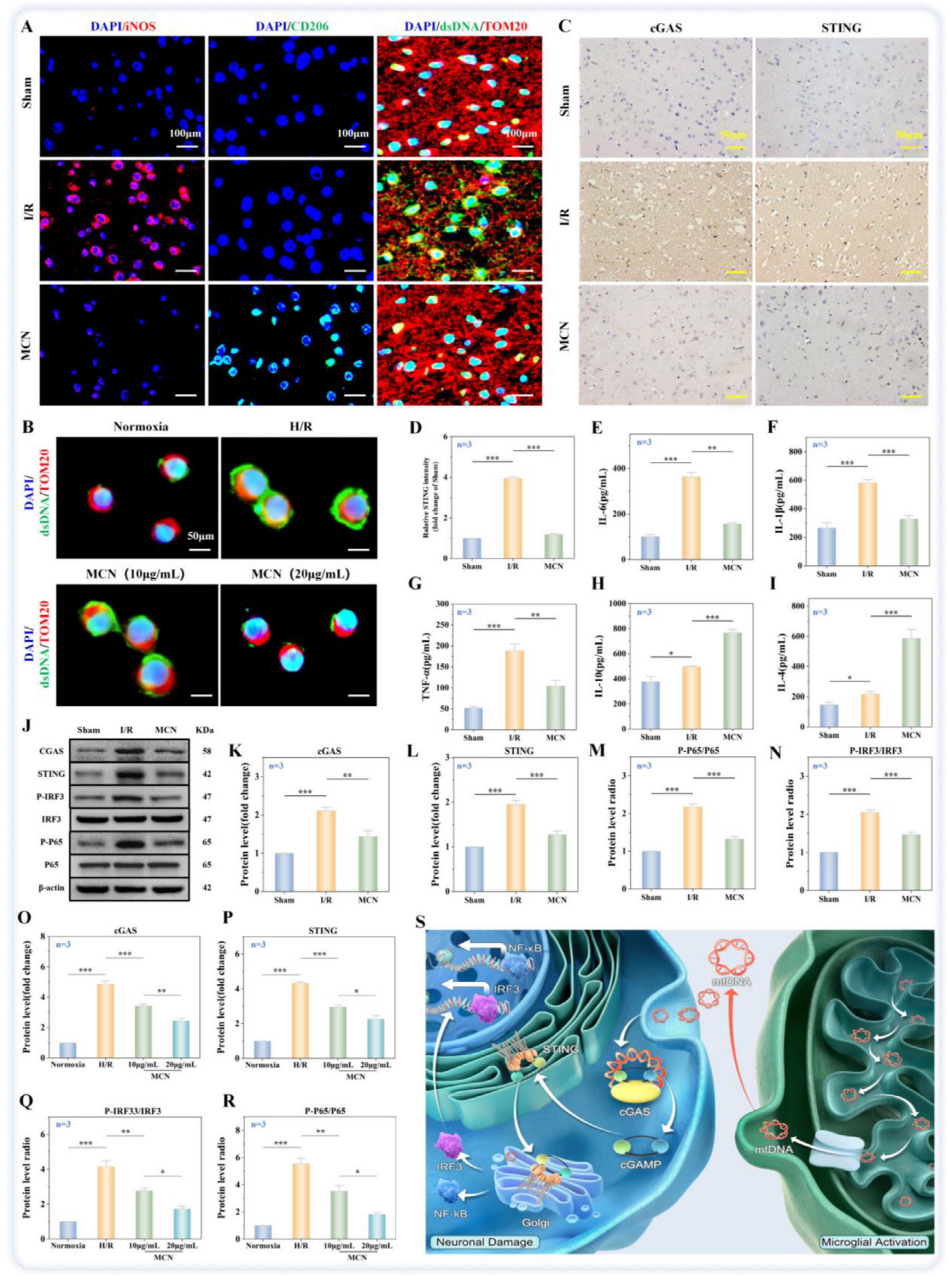
MCN inhibits cGAS‐STING pathway activation. (A) Representative fluorescence micrographs of immunofluorescence staining of iNOS (left), CD206 (center), and dsDNA/Tom20 (right) in cerebral tissue across all experimental groups. Scale bar: 10 µm. (B) Representative fluorescence micrographs of dsDNA/Tom20 immunofluorescence staining of BV2 cells across all experimental groups. Scale bar: 50 µm. (C) Representative fluorescence micrographs of cGAS (left) and STING (right) immunohistochemical staining in cerebral tissue across all experimental groups. Scale bar: 50 µm. (D) Quantification of STING immunohistochemical staining in cerebral tissue across all experimental groups. (E–G) Expression of pro‐inflammatory factors (TNF‐α, IL‐1β, IL‐4) in cerebral tissue across all experimental groups. (H,I) Expression of anti‐inflammatory factors (IL‐10, IL‐6) in cerebral tissue across all experimental groups. (J–R) Representative WB images (J) and Quantification (K–R) of inflammation‐related proteins (cGAS, STING, P‐IRF3/IRF3, P‐P65/P65) in cerebral tissue across all experimental groups. (S) Illustrated scheme of MCN inhibiting the cGAS‐STING signaling pathway to reverse neuroinflammation. All data are presented as the mean ± SE. For in vitro studies, three independent replicates were performed; for in vivo studies, each group consisted of three animals. Statistical significance was determined by one‐way ANOVA with Tukey post hoc test. ^*^ *P* < 0.05, ^**^ *P* < 0.01, ^***^ *P* < 0.001.

Ischemia‑reperfusion injury provokes oxidative stress, which in turn damages mitochondria and promotes mtDNA leakage, thus fueling neuroinflammation. We first characterized microglial phenotypes in lesioned cerebral tissue from the various treatment groups by staining M1‑type microglia with iNOS and M2‑type microglia with CD206. Figure 5A and Figure S40 show that in the I/R group, most microglia had adopted the M1 phenotype, with a CD206/iNOS ratio of 0.25. Remarkably, relative to the I/R group, the MCN‑treated group exhibited a marked reduction in M1‑polarized microglia and a substantial increase in M2‑polarized microglia, resulting in a CD206/iNOS ratio of 5.97. To further quantitatively validate the regulatory effect of MCN on microglial polarization, we employed qPCR to detect the mRNA expression of M1‐type markers (*iNOS* and *CD86*) and M2‐type markers *(Mrc1*(CD206) and *Arg1*). The results showed that, compared with the Sham‐operated group, the mRNA levels of *iNOS* and *CD86* in the I/R group were markedly elevated, while the levels of *Mrc1* and *Arg1* were markedly reduced. After MCN treatment, the expression of *iNOS* and *CD86* was markedly suppressed, whereas the levels of *Mrc1* and *Arg1* were significantly restored (Figure S41). These qPCR results are highly consistent with the immunofluorescence staining data described above, further confirming at the transcriptional level that MCN effectively inhibits microglial polarization toward the M1 phenotype and promotes their conversion to the M2 phenotype. This indicates that MCN regulates the phenotypic switch of microglia, thereby reversing neuroinflammation. Subsequently, we further investigated the specific mechanism by which MCN regulates the phenotypic switch of microglia. The localization pattern of mtDNA within I/R‐damaged cerebral tissue was visually assessed using a Tom20 probe to label mitochondria and a dsDNA probe to label mtDNA. As shown in Figure 5A, no aberrant mtDNA distribution was observed in the cerebral tissue of the Sham‐operated group. In contrast, a significant amount of mtDNA localized outside of mitochondria could be observed in the lesioned cerebral tissue of the I/R group, indicating that neuronal mitochondria suffered severe damage and released mtDNA. Notably, owing to the protective influence of neuronal mitochondria afforded by MCN, aberrant mtDNA distribution was also nearly absent within the injured cerebral tissue of animals receiving MCN. Furthermore, to validate this mechanism, we established an in vitro microglial inflammation model by co‐culturing BV2 microglial cells with HT22 neuronal cells to simulate the cerebral tissue microenvironment, aiming to further evaluate the anti‐inflammatory effects of MCN [33]. As depicted in Figure 5B, compared with the Normoxia group, the cytoplasm of BV2 cells in the H/R group contained a large amount of abnormally localized mtDNA, suggesting that mtDNA liberated from injured neurons can be taken up by BV2 cells and thereby elicit an inflammatory response. In line with the in vivo observations, MCN treatment significantly reduced the mtDNA content in BV2 cells in a concentration‑dependent manner, and at 20 µg/mL, abnormal mtDNA distribution was almost completely absent.

Given that the cGAS‑STING pathway serves as a critical axis for microglial recognition of mtDNA and subsequent polarization toward the M1 phenotype, we next examined the activation status of this pathway in cerebral tissue. Immunohistochemical analysis revealed that, relative to the Sham‐operated group, I/R injury markedly elevated the levels of cGAS and STING in the lesioned brain region, reaching 3.82 and 3.95 times the Sham values, respectively, indicative of robust cGAS‐STING pathway activation. As anticipated, MCN treatment strongly suppressed the I/R‑induced upregulation of cGAS and STING; in the MCN‑treated group, the levels of these proteins fell to 1.13 and 1.18 times those of the Sham‐operated group (Figure 5C,D; Figure S42).

We then directly assessed the ability of MCN to reverse neuroinflammation by measuring inflammatory cytokine levels in cerebral tissue and microglial cells. In vivo, the I/R group exhibited substantially elevated levels of the pro‑inflammatory factors IL‑1β, IL‑6, and TNF‑α, which rose to 2.19, 3.61, and 3.62 times the Sham‐operated group levels, respectively. In contrast, the MCN group showed significantly lower levels of these cytokines, which were reduced to 1.24, 1.55, and 2.01 times the Sham values, indicating that MCN effectively suppressed I/R‑induced pro‑inflammatory factor expression (Figure 5E–G). More importantly, MCN not only curbed the expression of pro‑inflammatory mediators in the ischemic cerebral lesion but also promoted the production of anti‑inflammatory factors, thereby favorably reshaping the local inflammatory microenvironment.

As presented in Figure 5H,I, the levels of the anti‑inflammatory cytokines IL‐4 and IL‐10 in cerebral tissue from the MCN‐treated group were markedly elevated, reaching 2.69 times and 1.54 times those measured in the I/R group, respectively. This finding indicates that MCN facilitates the phenotypic switching of microglia from the M1 to the M2 subtype, thereby mitigating neuroinflammation. Consistently, in vitro experiments demonstrated that MCN also suppressed the H/R‐induced upregulation of pro‐inflammatory factors while enhancing anti‐inflammatory factor expression in microglial cells (Figure S43). H/R challenge alone raised the levels of the pro‐inflammatory mediators IL‐1β, IL‐6, and TNF‐αto 4.04, 2.79, and 2.32 times the respective Normoxia values. Treatment with 10 µg/mL MCN effectively lowered these levels to 2.83, 2.13, and 1.73 times the Normoxia levels. An even more pronounced effect was observed with 20 µg/mL MCN, which reduced IL‐1β, IL‐6, and TNF‐α to 1.96, 1.26, and 1.42 times the Normoxia group values. Moreover, MCN dose‐dependently boosted the expression of the anti‐inflammatory factors IL‐4 and IL‐10: at 10 and 20 µg/mL, IL‐4 rose to 1.93 and 2.51 times the H/R group levels, while IL‐10 increased to 1.94 and 2.98 times the H/R group levels, respectively.

QPCR analysis corroborated these trends at the transcriptional level. In vivo, the mRNA levels of *IL‐1β*, *IL‐6*, and *TNF‐α* in the I/R group were increased to 2.49, 2.69, and 3.48 times those of the Sham‐operated group; after MCN intervention, they dropped to 1.02, 1.14, and 1.23 times the Sham values, respectively. Relative to the I/R group, the mRNA levels of *IL‐4* and *IL‐10* in the MCN group were elevated by 2.39‐ and 2.09‐fold, respectively (Figure S44). In vitro, exposure to H/R elevated the mRNA levels of *IL‑1β*, *IL‑6*, and *TNF‑α* to 2.32, 2.53, and 2.92 times those of the Normoxia group, respectively. Following treatment with MCN at 10 and 20 µg/mL, the *IL‑1β* transcript levels declined to 1.70 and 1.22 times the Normoxia values; *IL‑6* dropped to 1.83 and 1.39 times; and *TNF‑α* fell to 2.14 and 1.27 times, respectively. Relative to the H/R group, the mRNA abundance of *IL‑4* rose by factors of 1.65 (10 µg/mL) and 2.22 (20 µg/mL), while IL‑10 increased by 1.52‑ and 2.60‑fold, respectively. These qPCR results are consistent with the ELISA data and provide further evidence that MCN regulate the expression of both pro‑inflammatory and anti‑inflammatory cytokines (Figure S45).

Western blot(WB) analysis was further performed to examine the protein levels of cGAS‑STING pathway components in lesioned cerebral tissue from the different treatment groups. As shown in Figure 5J–N, MCN treatment reversed the I/R‑induced elevation of cGAS and STING, as well as the phosphorylation of their downstream effectors IRF3 and P65, bringing these levels close to those seen in the Sham‐operated group. These results provided additional confirmation that MCN suppressed activation of the cGAS‑STING signaling pathway in microglia during CIRI. Specifically, relative to the Sham‐operated group, the I/R group exhibited increases in cGAS, STING, p‑IRF3/IRF3, and p‑P65/P65 by factors of 2.12, 1.96, 2.05, and 2.18, respectively; in the MCN group, these values were reduced to 1.44, 1.27, 1.46, and 1.32 times those of the Sham‐operated group, respectively.

Consistent with the in vivo findings, MCN also significantly attenuated the H/R‑induced upregulation of cGAS, STING, and the phosphorylation of IRF3 and P65 in BV2 cells within the in vitro neuron‑microglia co‑culture model (Figure 5O–R; Figure S46). In BV2 cells, H/R challenge increased the levels of cGAS, STING, p‑IRF3/IRF3, and p‑P65/P65 to 4.85, 4.32, 4.15, and 5.55 times those of the Normoxia group, respectively. Treatment with 10 µg/mL MCN restored these values to 3.42, 2.96, 2.77, and 3.53 times the Normoxia levels, while 20 µg/mL MCN further reduced them to 2.45, 2.25, 1.73, and 1.83 times the Normoxia group values, respectively.

To further elucidate the activation state of STING within the cGAS‑STING axis and the ensuing type I interferon response, we assessed the phosphorylation level of STING (p‑STING) in cerebral tissue and BV2 cells using Western blot (WB), while concurrently measuring IFN‑β content via ELISA. As presented in Figure S47, in cerebral tissue, the p‑STING level in the I/R group rose to 1.94 times that of the Sham‐operated group, indicating STING activation. Following MCN treatment, this value dropped to 1.25 times the Sham‐operated group level. In BV2 cells, the p‑STING level in the H/R group increased to 2.72 times the Normoxia group value. After exposure to low‑dose (10 µg/mL) and high‑dose (20 µg/mL) MCN, the p‑STING levels declined to 1.64 and 1.11 times the Normoxia group levels, respectively.

ELISA measurements revealed that the IFN‑β level in cerebral tissue was elevated to 2.71 times the Sham‐operated group level in the I/R group, and was reduced to 1.45 times after MCN treatment. In BV2 cells, the IFN‑β level in the H/R group increased to 2.79 times the Normoxia group value, and decreased to 1.98 and 1.48 times following administration of low and high doses of MCN, respectively (Figure S48). These observations are in strong agreement with the alterations in cGAS, STING, p‑IRF3, and p‑P65 expression, providing further evidence that MCN potently suppresses cGAS‑STING pathway activation, thereby abrogating the downstream type I interferon response and neuroinflammation.

To further verify that mtDNA released from neurons serves as a crucial trigger for microglial activation, we first carried out immunofluorescence staining in HT22 neurons. As illustrated in Figure S49, following H/R exposure, abundant dsDNA signals colocalizing with mitochondria appeared in the neuronal cytoplasm, indicating that mtDNA had escaped from mitochondria into the cytosol; this signal was markedly diminished by MCN treatment. We next separated cytoplasmic and mitochondrial fractions and measured the relative mtDNA copy number by qPCR. In the H/R group, the cytoplasmic mtDNA copy number was elevated by approximately 5.99 times relative to the Normoxia group, whereas the mtDNA copy number in the mitochondrial fraction fell to 0.30 times the control level. After treatment with MCN at 10 and 20 µg/mL, the cytoplasmic mtDNA decreased to 1.61 and 1.20 times the control values, respectively, while the mitochondrial mtDNA recovered to 0.51 and 0.88 times the control levels, respectively. To further determine whether mtDNA leakage is dependent on mPTP opening, we pre‑treated neurons with the mPTP inhibitor cyclosporine A (CsA, 5 µm). As shown in Figure S50, CsA treatment alone significantly suppressed the H/R‑induced rise in cytoplasmic mtDNA (reducing it to 1.33 times the control level), and no additive effect was observed when CsA was combined with MCN treatment. These findings indicate that MCN reduces mtDNA leakage by inhibiting mPTP opening.

To further verify that the mtDNA responsible for activating BV2 cells in the co‑culture system originates predominantly from neurons rather than from the BV2 cells themselves, we harvested the supernatant of HT22 neurons from various treatment groups to serve as conditioned medium (CM), specifically Normoxia CM, H/R CM, and H/R + MCN CM. Direct H/R exposure of BV2 cells was included as a positive control. These conditioned media were then added to naive BV2 cells and incubated for 24 h, after which the supernatants were collected for measurement of inflammatory cytokine levels. ELISA results revealed that, in BV2 cells exposed to H/R CM, the secreted levels of the pro‑inflammatory cytokines IL‑1β, TNF‑α, and IL‑6 rose to 3.98, 2.43, and 3.24 times those observed in the Normoxia CM group, respectively. By contrast, the H/R+MCN CM group markedly reversed these elevations, bringing the cytokine levels to values comparable to those in the Normoxia CM group. Direct H/R treatment of BV2 cells did not trigger a notable inflammatory response. Moreover, the levels of the anti‑inflammatory cytokines IL‑4 and IL‑10 in the H/R+MCN CM group were significantly increased, reaching 2.58 and 2.26 times the levels measured in the I/R group, respectively, findings that align closely with the observations from the co‑culture system (Figure S51). Collectively, these results demonstrate that mtDNA released from neurons following H/R injury (an event dependent on mPTP opening) is sufficient to activate microglia, and that MCN indirectly suppresses microglial inflammation by preserving neuronal mitochondrial integrity and limiting mtDNA leakage.

To summarize, microglia readily recognize mtDNA released by damaged neurons and quickly convert to a pro‐inflammatory M1 phenotype via the cGAS‐STING signaling cascade, which in turn sets off neuroinflammation (Figure 5S). The accumulated evidence shows that MCNs robustly preserve neuronal mitochondrial integrity, reduce mtDNA leakage from neurons, interrupt cGAS‑STING activation in microglia, and thereby facilitate the phenotypic switch of microglia from M1 to M2, ultimately ameliorating neuroinflammation.

### 2.6 MCN Improves Endoplasmic Reticulum Stress

**FIGURE 6:**
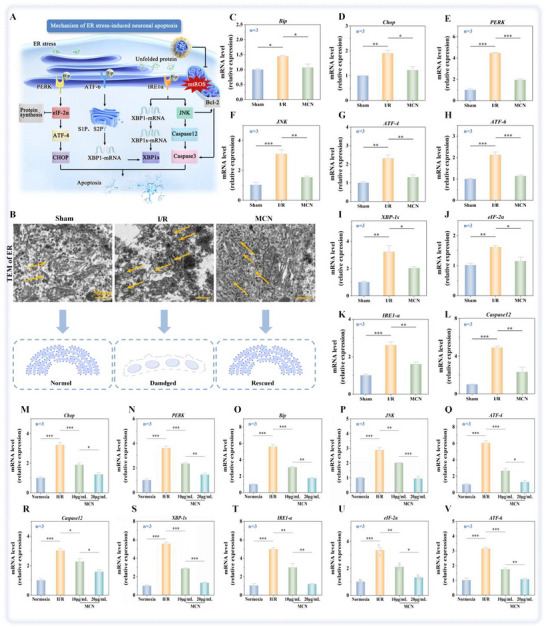
MCN inhibits ER stress. (A) Illustrated scheme of MCN inhibiting ER stress during CIRI through intrinsic and extrinsic pathways. (B) TEM images of ER in neurons in cerebral tissue. Scale bar: 500 nm. (C–L) QPCR was used to detect the expression levels of ER stress‐related factors (*Bip*, *PERK*, *IRE1‐α*, *ATF‐6*, *eIf‐2α*, *ATF‐4*, *Chop*, *XBP‐1s*, *JNK*, *and Caspase 12*) in cerebral tissue across all experimental groups. (M–V) QPCR was used to detect the expression levels of ER stress‐related factors (*Bip*, *PERK*, *IRE1‐α*, *ATF‐6*, *eIf‐2α*, *ATF‐4*, *Chop*, *XBP‐1s*, *JNK*, *and Caspase 12*) in HT22 cells across the various treatment groups. All data are presented as the mean ± SE. For in vitro studies, three independent replicates were performed; for in vivo studies, each group consisted of three animals. Statistical significance was determined by one‐way ANOVA with Tukey post hoc test. ^*^ *P* < 0.05, ^**^ *P* < 0.01, ^***^ *P* < 0.001.

Neuronal mitochondria are in direct contact with the ER. Mitochondrial damage and the resulting excessive production of mtROS can trigger ER stress. As a crucial organelle, the ER is responsible for synthesizing, folding, and modifying secretory and transmembrane proteins. When ER stress occurs, these proteins become misfolded or unfolded and accumulate. The signaling pathways associated with this process include the *IRE1α* pathway, the *PERK* pathway, and the *ATF6* pathway [34]. Under normal conditions, the molecular chaperone binding immunoglobulin protein (*Bip*) inhibits these three pathways. When stress occurs, it leads to the activation of these pathways and their downstream signaling [35]. Specifically, *Bip* dissociates from *IRE1α*, leading to *IRE1α* activation. Activated *IRE1α* can activate the protein kinase *JNK* and the downstream signal *Caspase‐12*, thereby promoting the activation of apoptotic pathways. Furthermore, through its protein kinase and ribonuclease activities, activated *IRE1α* catalyzes the splicing of *XBP1* mRNA, generating the transcriptionally active spliced form *XBP1s*. *XBP1s* then translocates to the nucleus, where it regulates the expression of downstream stress‐responsive genes [36]. The dissociation of Bip activates *PERK*, which in turn promotes the phosphorylation of eukaryotic translation initiation factor 2α (*eIF2α*). On one hand, this phosphorylation slows down overall protein synthesis within the cell, alleviating the folding burden on the ER. On the other hand, it facilitates the translation of specific stress‐related genes, particularly activating transcription factor 4 (*ATF4*) [37, 38, 39]. Once activated, *ATF4* becomes involved in regulating the expression of various stress response genes. This includes genes that promote cellular adaptation and survival, such as antioxidant enzymes and molecular chaperones, which help the cell combat stress and maintain function. However, *ATF4* can also promote the expression of pro‐apoptotic genes, such as C/EBP homologous protein (*CHOP*). The overexpression of *CHOP* is closely linked to ER stress‐induced apoptosis [40]. ATF6 translocates to the Golgi apparatus, where it is cleaved by site‐1 protease (*S1P*) and site‐2 protease (*S2P*), generating an active fragment containing a nuclear localization signal. This fragment then cooperates with *XBP1s* to regulate the ER stress response. This series of ER‐related signaling pathways forms an interconnected cascade (Figure 6A). Therefore, we investigated the role of MCN in improving ER stress during CIRI. First, we directly observed morphological changes in the ER within cerebral tissue using TEM. The results revealed that I/R treatment caused the ER to become significantly enlarged and edematous. Intervention with MCN restored/maintained the normal morphology of the ER, indicating that MCN can alleviate ER stress, thereby preserving the normal morphology and physiological function of the ER (Figure 6B). Subsequently, the expression levels of ER stress‐related factors in cerebral tissue and HT22 cells from different treatment groups were further detected using qPCR technology. In vivo experimental results showed that compared to the Sham‐operated group, the expression of *Bip*, *PERK*, *IRE1*, *ATF‐6*, and their downstream molecules (*eIF‐2α*, *ATF‐4*, *Chop*, *XBP‐1s*, *JNK*, and *Caspase 12*) was significantly upregulated in the cerebral tissue of the I/R group, indicating that I/R stimulation can trigger a severe ER stress response. However, intervention with MCN effectively reversed the expression levels of these factors, significantly suppressing ER stress (Figure 6C–L). In vitro experiments further confirmed that MCN could also markedly improve the ER stress state in H/R‐induced HT22 cells (Figure 6M–V).

To further validate at the protein level that MCN suppresses endoplasmic reticulum stress, we examined the expression and activation status of key ER stress‑related proteins in cerebral tissue and HT22 cells by Western blot. In cerebral tissue, relative to the Sham‐operated group, the I/R group exhibited elevated ratios of p‑eIF2α/eIF2α, along with increased protein levels of CHOP, XBP1s, and cleaved Caspase‑12, reaching 2.02, 2.52, and 2.86 times those of the Sham‐operated group, respectively. Following MCN treatment, these values fell to 1.25, 1.63, and 1.57 times the Sham levels, approaching those seen in the Sham‐operated group. In HT22 cells, the H/R group showed rises in the same parameters to 3.56 times (p‑eIF2α/eIF2α), 3.85 times (CHOP), 2.99 times (XBP1s), and 3.08 times (cleaved Caspase‑12) the Normoxia group values. Upon treatment with MCN at 10 and 20 µg/mL, all indicators declined in a concentration‑dependent manner: the p‑eIF2α/eIF2α ratio dropped to 2.61 and 1.28 times the Normoxia levels; CHOP decreased to 2.47 and 1.32 times; XBP1s fell to 1.86 and 1.12 times; and cleaved Caspase‑12 decreased to 2.17 and 1.19 times, respectively. These Western blot findings are in strong agreement with the qPCR data, providing further confirmation that MCN effectively attenuates ER stress and its downstream pro‑apoptotic signaling (Figures S52–S54).

### 2.7 MCN Inhibits Neuronal Apoptosis

**FIGURE 7:**
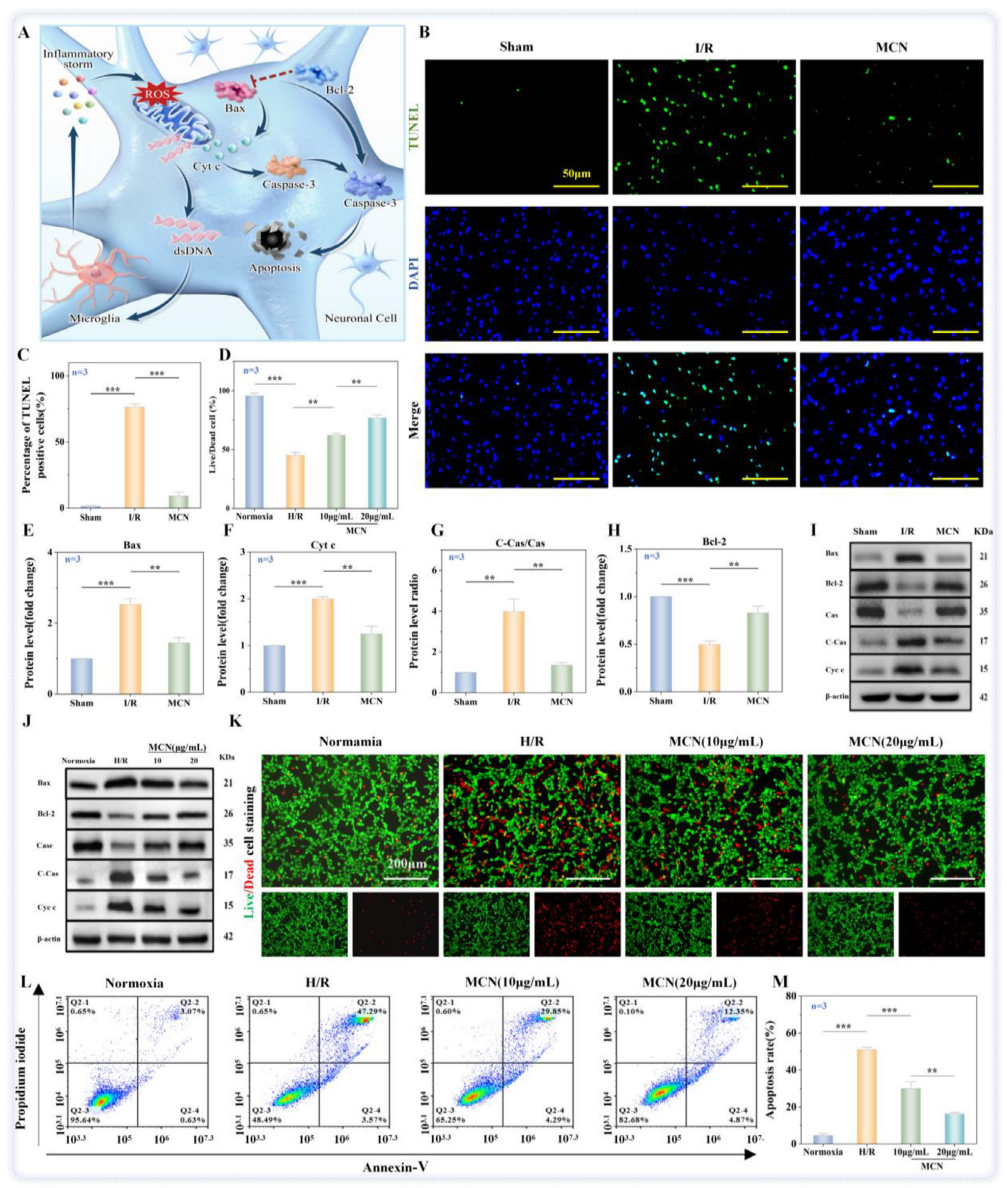
MCN inhibits neuronal apoptosis. (A) Illustrated scheme of apoptosis improvement by MCN. (B) TUNEL staining in cerebral tissue across all experimental groups. Scale bar: 50 µm. (C) Quantitative statistics of TUNEL immunofluorescence staining in cerebral tissue. (D) Quantitative statistics of Live/Dead cell immunofluorescence staining in HT22 cells. (E–I) Representative images and statistical analysis of apoptosis‐related protein (Bax, Bcl‐2, C‐Cas/Cas, Cyt c) expression in cerebral tissue across all experimental groups. (J) Representative images of apoptosis‐related protein (Bax, Bcl‐2, C‐Cas/Cas, Cyt c) expression in HT22 cells across the various treatment groups. (K) Representative fluorescence micrographs of Live/Dead cell staining in HT22 cells across the various treatment groups. Scale bar: 200 µm. (L,M) Representative graphs and statistical analysis of HT22 cell apoptosis rates in different treatment groups detected by flow cytometry. All data are presented as the mean ± SE. For in vitro studies, three independent replicates were performed; for in vivo studies, each group consisted of three animals. Statistical significance was determined by one‐way ANOVA with Tukey post hoc test. ^**^ *P* < 0.01, ^***^ *P* < 0.001.

Mitochondrial damage and ER stress can further exacerbate the process of apoptosis (Figure 7A) [41]. Encouragingly, the experimental data presented above demonstrate that MCN possesses a potent capacity to counteract mitochondrial damage, ER stress, and neuroinflammation. We therefore proceeded to directly evaluate the anti‑apoptotic effect of MCN. Terminal deoxynucleotidyl transferase dUTP nick end labeling (TUNEL) staining detects apoptotic cells by recognizing DNA fragmentation and is capable of visualizing apoptotic nuclei and apoptotic bodies within tissues. As shown in Figure 7B,C, the proportion of apoptotic cells in the ischemic cerebral tissue of the I/R group reached 76.49%. MCN treatment robustly suppressed I/R‑induced apoptosis, lowering the apoptotic percentage to 9.35%, thus indicating that MCN markedly inhibits cellular apoptosis.

Subsequent Western blot analysis was carried out to quantify the expression of apoptosis‑related proteins in lesioned cerebral tissue across the different treatment groups. The I/R group exhibited significant elevations in the pro‑apoptotic proteins Bax, cleaved caspase‑3/caspase‑3, and cytochrome c (Cyt‐c), with levels rising to 2.53, 3.98, and 2 times those of the Sham‐operated group, respectively. Administration of MCN substantially reversed these I/R‑induced increases, reducing the values to 1.45, 1.36, and 1.25 times the Sham‐operated group levels. Moreover, MCN strongly enhanced the expression of the anti‑apoptotic protein Bcl‑2 in cerebral tissue: Bcl‑2 expression in the I/R group fell to 0.50 times the Sham level, whereas after MCN treatment it recovered to 0.83 times the Sham level (Figure 7E–I).

Similar trends were observed in vitro in H/R‑challenged HT22 cells (Figure 7J; Figure S55). H/R exposure markedly elevated the levels of Bax, cleaved caspase‐3/caspase‐3, and Cyt‐c to 2.36, 7.61, and 5.37 times the Normoxia group values, respectively. MCN treatment significantly reduced these pro‑apoptotic protein levels in a dose‑dependent manner: at 10 µg/mL, they fell to 1.73, 2.52, and 3.37 times the Normoxia values; at 20 µg/mL, they were further decreased to 1.27, 1.48, and 2.07 times, respectively. In addition, relative to the Normoxia group, Bcl‑2 expression in the H/R group was only 0.40 times the control level; it increased to 0.63 times with 10 µg/mL MCN and to 0.84 times with 20 µg/mL MCN.

We also employed live/dead cell staining to further assess the protective effect of MCN against H/R‑induced cell death. As shown in Figure 7D,K, H/R stimulation reduced the proportion of viable HT22 cells to 47.20% of the Normoxia group. Treatment with 10 µg/mL MCN raised the viable cell fraction to 65.13% of the Normoxia group, while 20 µg/mL MCN restored it to a level comparable to the Normoxia group (80.49% of the Normoxia group). Flow cytometry analysis further confirmed the anti‑apoptotic action of MCN (Figure 7L,M). The apoptosis rate in the H/R group was 51.09%; MCN treatment dose‑dependently lowered this rate to 29.97% at 10 µg/mL and further to 16.30% at 20 µg/mL. In summary, these findings collectively demonstrate that MCN are highly effective at suppressing neuronal apoptosis.

### 2.8 Biocompatibility and Safety of MCN

Last, the biocompatibility and safety profile of MCN were examined. Initially, normal HT22 and BV2 cells were exposed to various concentrations of MCN under in vitro conditions. The CCK‑8 assay was employed to measure cell viability at 24 and 48 h after treatment. It was found that even at a dose as high as 160 µg/mL of MCN (which is 8 times the effective dose), the viability of both cell types showed no difference compared to cells without drug intervention, indicating that MCN possesses good biocompatibility at the cellular level (Figures S56 and S57).

**FIGURE 8:**
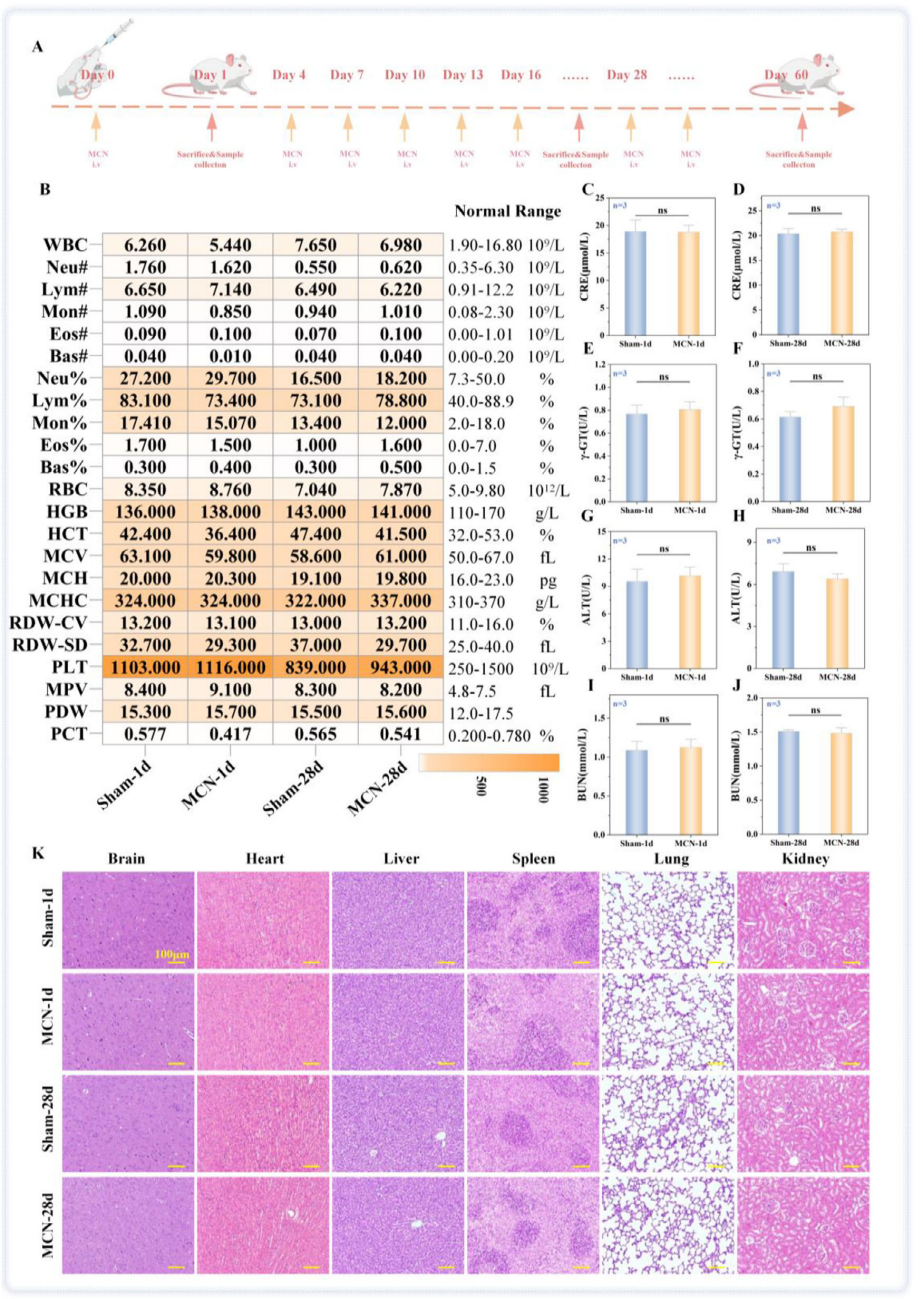
Biocompatibility of MCN. (A) Illustrated scheme of toxicology experiment administration in rats. (B) Heatmap of Complete Blood Count for 1, 28, and 60 days. (C) Creatinine clearance rate for 1 day. (D) Creatinine clearance rate for 28 days. (E) γ‐glutamyl transpeptidase for 1 day. (F) γ‐glutamyl transpeptidase for 28 days. (G) Alanine Aminotransferase for 1 day. (H) Alanine Aminotransferase for 28 days. (I) Blood Urea Nitrogen for 1 day. (J) Blood Urea Nitrogen for 28 days. (K) Representative images of HE staining of major organs of rats for 1, 28, and 60 days. Scale bar: 100 µm. All data are presented as the mean ± SE. Each group consisted of three animals. Statistical significance was determined by one‐way ANOVA with Tukey post hoc test. ns: *P* > 0.05.

To further evaluate the biosafety of MCN, we conducted systematic validation experiments at the animal level. A tenfold therapeutic dose of MCN was administered to rats via multiple sublingual venous injections. Multidimensional assessments were performed at 1 day (acute toxicity), 28 days (subchronic toxicity), and 60 days (chronic toxicity post‐administration (Figure 8A). First, we assessed the biocompatibility and biosafety of MCN via complete blood count (CBC) analysis. As shown in Figure 8B and Figure S58, all CBC parameters of rats in the MCN‐administered groups at both 1, 28, and 60 days post‐treatment were within the normal ranges and showed no significant differences compared to the Sham‐operated group. This indicates that sublingual venous injection of MCN does not cause abnormalities in rat blood parameters. Subsequently, further serum biochemical index tests revealed that liver function indicators (Alanine Aminotransferase (ALT) and γ‐glutamyl transpeptidase (γ‐GT)) and kidney function indicators (Creatinine (CR) and Blood Urea Nitrogen (BUN)) in rats from the MCN‐administered groups showed no statistically significant differences compared to the normal control group, confirming that MCN have no impact on liver and kidney function (Figure 8C–J; Figure S59). Finally, HE staining of major organs including the brain, heart, liver, spleen, lungs, and kidneys from rats administered MCN for 1, 28, and 60 days revealed no observable morphological or structural abnormalities, indicating no damaging effects on organ tissues (Figure 8K; Figure S60). The above results demonstrate that even at doses several times the therapeutic level, MCN exhibits excellent biocompatibility and safety both in vitro and in vivo.

## 3 Conclusion

This study reports the construction of a novel MCN via an active ligand self‐assembly strategy. By employing the Quer as the self‐assembling ligand and coordinating Quer with iron ions, MCN concurrently addresses its intrinsic limitations of poor aqueous solubility and low stability. Simultaneously, MCN constructs dynamic Fe^2+^/Fe^3+^ valence‐changing catalytic centers, which enable a transition from an exogenous antioxidant to an endogenous catalytic platform, and endow MCN with efficient SOD‐CAT cascade enzyme‐like activities. Mechanistic studies reveal that MCN can traverse the compromised BBB and specifically accumulate in the ischemic brain region. More importantly, we confirm that Quer possesses an intrinsic, high‐affinity interaction with mitochondrial outer‐membrane proteins, enabling MCN to precisely target neuronal mitochondria. At this epicenter of injury, MCN efficiently scavenges ROS via cascade catalysis, stabilizing the MMP and maintaining ATP synthesis, thus maintaining both the architecture and functionality of mitochondria. This fundamental protection directly blocks the leakage of the pro‐inflammatory mediator mtDNA, severs the activation of the mtDNA‐cGAS‐STING signaling axis, and subsequently drives the phenotypic shift of microglia from the M1 to the M2 type. This reshapes the cerebral inflammatory microenvironment, dismantling at its source the vicious cycle of oxidative stress‐mitochondrial damage‐neuroinflammation. In summary, we for the first time unveil and harness the inherent mitochondrial‐targeting property of the natural active molecule Quer, and offer a potent, precise, and potentially safe novel therapeutic approach for CIRI. This work not only provides new insights for the high‐value pharmaceutical application of Quer and other polyphenolic natural products, but also offers valuable conceptual reference and a design paradigm for developing next‐generation intelligent, synergistic nanotherapeutics targeting dynamic, multi‐pathway pathological networks such as stroke.

## 4 Materials and Methods

### 4.1 Synthesis of MCN

A 20 mg/mL solution of FeCl_3_·6H_2_O in methanol, a 13.2 mg/mL solution of PVP, and a 10 mg/mL solution of Quer were prepared. Subsequently, the prepared FeCl3 · 6H2O solution was added dropwise to the PVP solution at a volume ratio of 1:5 and mixed thoroughly by ultrasonication. The resulting mixture was then added dropwise to the Quer solution at a volume ratio of 1:6 (the final molar ratio of Quer to Fe^3+^ was approximately 16:1). The combined solution was stirred vigorously and allowed to react at room temperature for 3 h. The initial pH of the reaction mixture was approximately 5.5 (without external adjustment). The obtained deep green liquid was then dialyzed against pure water for 12 h using a dialysis membrane with a molecular weight cut‐off (MWCO) of 3500 Da to remove excess iron ions and methanol, yielding the final water‑soluble MCN. The dialyzed MCN solution was freeze‑dried to obtain MCN powder, which was stored at −20°C protected from light.

### 4.2 Synthesis of MCN‐BODIPY

Prepared the aforementioned 20 mg/mL FeCl_3_·6H_2_O solution dissolved in methanol and 13.2 mg/mL PVP solution. Mixed these solutions in a 1:5 ratio to obtain Solution A. Prepared a 20 mg/mL Quer solution in methanol. Subsequently, prepared a 1 mg/mL BODIPY solution in methanol, ensuring complete dissolution. Used this solution to dilute the 20 mg/mL Quer solution to a concentration of 10 mg/mL, yielding Solution B. Combined solutions A and B in a flask, stirred, and reacted for 3, then dialyzed again in ultrapure water for 24 h before centrifuging to obtain MCN‐BODIPY.

### 4.3 The Drug Loading Efficiency and Encapsulation Efficiency of MCN

A standard calibration curve for Quer was established by ultraviolet–visible spectroscopy (UV‐Vis). During the loading process, 100 µL of supernatant was collected after ultracentrifugation, and its absorbance was measured. The concentration of free Quer in the supernatant was determined using the established calibration curve. The encapsulation efficiency and drug loading were calculated according to the following formulas:

Encapsulation efficiency (%) = (Total Quer added − Free Quer in supernatant)/Total Quer added × 100%

Drug loading(%) = (Total Quer added − Free Quer in supernatant)/Mass of lyophilized MCN powder × 100%

### 4.4 Characterization of MCN

TEM images were captured using a high‐resolution transmission electron microscope; the zeta potential and particle size of MCN were determined via dynamic light scattering; FTIR analyzed the functional group structure of MCN; XRD patterns characterized the phase structure of MCN; and XPS detected the elemental composition of MCN. Absorption characteristics were determined using an Aoyi Instruments A580 UV spectrophotometer.

### 4.5 Colocalization of HT22 With MCN‐BODIPY

HT22 cells were seeded at a density of 1 × 10^5^ cells per well in 12‐well plates. Following cell attachment, the cells were incubated in medium supplemented with MCN‐BODIPY. Co‐localization of MCN‐BODIPY with HT22 cells was observed under a fluorescence microscope, and images were captured.

### 4.6 Molecular Docking

Molecular docking analysis was performed using the MOE 2022 software on all selected compounds via the standard script method. Protein codes were as follows: TOM7: AF‐Q9P0U1‐F1, TOM20: AF‐Q15388‐F1, TOM34: AF‐Q15785‐F1, TOM40: AF‐O96008‐F1, TOM70: AF‐O94826‐F1, VDAC1: AF‐P21796‐F1, VDAC2: AF‐P45880‐F1.

### 4.7 Degradation of MCN and Quer

The MCN was allowed to stand at 37°C, protected from light, for 0, 1, 3, 6, 12, 24, and 72 h. The UV–vis spectrophotometer was used to detect the UV‐absorption spectra of the MCN solutions at different time points of standing. The Agilent/1290 Infinity II analytical liquid chromatography purification system was used to determine the content of MCN solutions at different time points. A digital camera was used to record the color change of the MCN solution at different time points during resting.

### 4.8 Determination of SOD‐Like and CAT‐Like Specific Activities

#### 4.8.1 SOD‐Like Specific Activity Assay

The SOD‐like activity of MCN was determined using the WST‐1 method (Solarbio, BC5160). MCN was diluted with PBS buffer to a series of concentrations (0–40 µg/mL). In a 96‐well plate, the sample, WST‐1 working solution, and enzyme working solution (xanthine oxidase) were added sequentially according to the manufacturer's instructions. After mixing, the plate was incubated at 37°C for 20 min, and the absorbance was measured at 450 nm using a microplate reader. The inhibition rate was calculated, and the SOD activity (*U*) was determined using a standard curve provided by the kit. Specific activity was defined as enzyme activity units per milligram of MCN (U/mg).

#### 4.8.2 CAT‐Like Specific Activity Assay

The CAT‐like activity of MCN was determined using the ammonium molybdate method (Nanjing Jiancheng, A007‐1‐1). MCN was diluted to an appropriate concentration with PBS buffer. According to the kit protocol, MCN and the substrate H_2_O_2_ (10 mm) were added to the reaction system, incubated in a water bath at 37°C for 1 min, and then the ammonium molybdate stop solution was added. After mixing, the absorbance was measured at 405 nm. CAT activity (*U*) was calculated using a standard curve, and specific activity was expressed as U/mg.

### 4.9 Detection of the Ability to Remove Superoxide Anions (O_2_^.−^) of MCN

The NBT assay was employed to assess the scavenging capacity of MCN toward O_2_^.−^. In the presence of methionine, riboflavin underwent photochemical reduction to generate O_2_^.−^. This radical reduced NBT to form blue methylhydrazine, exhibiting maximum absorption at 560 nm. The absorbance at 560 nm was measured to quantify MCN's scavenging capacity for O_2_^.−^. In a cuvette, sequentially added 390 µL methionine (0.1 m), 7 µL riboflavin (20 µm), 23 µL NBT (0.01 m), and 15 µL MCN at different concentrations (0, 2.5, 5, 10, 20, and 40 µg/mL) of MCN into a cuvette, followed by 1.5 mL phosphate‐buffered saline (PBS, 0.1 m, pH 7.4) and 1.065 mL deionized water to form a 3 mL total volume system. The mixture was thoroughly mixed, and the baseline was determined. After irradiating the cuvette with UV light for 5 min, the absorbance at 560 nm was measured.

### 4.10 Detection of the Ability to Scavenge Hydroxyl Radicals (·OH) of MCN

The scavenging capacity of MCN toward ·OH radicals was assessed via the oxidative reaction of TMB. The Fenton reaction between FeSO_4_ and H_2_O_2_ catalyzed the generation of ·OH radicals, which oxidized TMB to form a colored product. This colored substance served as an indicator for the concentration of ·OH radicals in solution. A 25 mm TMB solution prepared in dimethyl sulfoxide was mixed with an appropriate volume of 0.1 m hydrogen peroxide, PBS (0.01 m, pH 7.4), and MCN solutions at various concentrations (0, 5, 10, 20, 40, and 80 µg/mL). The mixture was allowed to react at room temperature for 7 min. Upon completion of the reaction, the absorbance at 652 nm was measured by UV–vis spectrophotometry to analyze the MCN scavenging capacity for ·OH radicals.

### 4.11 Detection of the Ability to Scavenge Peroxynitrite Anion (ONOO^−^) of MCN

Different concentrations of ONOO^−^ (0, 0.031, 0.0625, 0.125, 0.25, 0.5 µm) were reacted with MCN. After incubation at 37°C in the dark for 12 h, the absorbance at 652 nm was measured using UV–vis spectrophotometry to determine the scavenging efficiency of MCN toward ONOO^−^.

### 4.12 Detection of the Ability to Scavenge Hydrogen Peroxide (H_2_O_2_) of MCN

H_2_O_2_ solutions at various concentrations (0, 50, 100, 200, 400, and 800 µm) were mixed with MCN and incubated at 37°C in the dark for 12 h. The H_2_O_2_ scavenging efficiency of MCN was measured by detecting UV absorption at 425 nm using UV–vis spectrophotometry.

### 4.13 Construction of Animal Models of CIRI

SPF‐grade male SD rats, body weight (260–280 g), supplied by Hunan SJA LABORATORY ANIMAL Co., Ltd., License No.: SCXK (Xiang) 2019‐0004. The animal experimentation protocol was approved by the Ethics Committee of the Laboratory Animal Center, the First Hospital of Hunan University of Chinese Medicine (Ethics No.: 202404024).

### 4.14 Construction of the CIRI Model

The middle cerebral artery occlusion (MCAO) technique was employed to simulate the cerebral ischemia phase, whilst cessation of arterial occlusion and restoration of blood flow simulated the reperfusion phase to establish the CIRI model. SD rats were deeply anaesthetized with pentobarbital, then fixed supine on the operating table following shaving and disinfection. A longitudinal neck incision was made, sequentially dissecting the left common carotid artery (CCA), external carotid artery (ECA), and internal carotid artery (ICA). The ECA was ligated with nylon suture near its bifurcation with the CCA and clamped with an arterial clamp. A small ‘V’‐shaped incision was made at the proximal end of the CCA.

A silicone‐coated monofilament suture (2838A4, diameter 0.28 mm, tip diameter 0.38 mm; Beijing Sinon Biotechnology Co., Ltd., China) was gently inserted through the incision into the ICA. The suture was advanced until a distinct resistance was felt (approximately 20–22 mm from the carotid bifurcation), indicating successful occlusion of the middle cerebral artery. The thread was then secured within the vessel.

A saline‐soaked cotton ball was applied to the rat's neck wound to maintain moisture, and the animal was placed under an electric blanket for warmth. Following 2 h of ischemia, sublingual venous administration was performed: the sham‐operated group and model group received equal volumes of saline solution, respectively. The embolization thread was removed and the wound sutured, then disinfected with povidone‑iodine. Upon recovery, the rat was returned to its cage for a 24 h post‐operative period.

### 4.15 Confirmation of MCAO Model

Cerebral blood flow in both hemispheres of rats was monitored using a laser Doppler flowmeter (RFLSI III, RWD). After anesthesia, MCAO rats were fixed on a stereotaxic instrument and placed under the laser probe of the flowmeter. Blood flow in the left and right hemispheres was observed. A significant decrease (>70%) in blood flow in the left hemisphere (ischemic side) compared to the right hemisphere (contralateral control side) indicated successful occlusion of the middle cerebral artery.

### 4.16 Cell Culture

The HT22 and BV2 cell lines were both procured from the Chinese Academy of Sciences Cell Bank. HT22 cells were cultured in DMEM medium supplemented with 10% fetal bovine serum, whilst BV2 cells were cultured in DMEM medium supplemented with 10% newborn calf serum. All cells were maintained at 37°C in a 5% CO_2_ environment.

### 4.17 Construction of a H/R Model in HT22 Cells

HT22 cells were incubated in medium containing 400 µm cobalt chloride for 16 h to simulate the cellular hypoxia phase, followed by recovery in normal medium for 8 h to simulate the reoxygenation phase, thereby establishing an H/R model.

### 4.18 Establishment of a Model in HT22 Cells

Commissioned Sangon Biotech to construct the corresponding siRNAs for Tomm20. The above siRNAs were introduced into H9c2 cells using the transfection reagent Lipo3000 (Thermo Fisher). After transfection for 6–8 h, the medium was replaced, and the cells were cultured for an additional 24 h before proceeding with subsequent experiments.

### 4.19 Establishment of OGD/R in HT22 Cells

To establish an OGD/R model, the culture medium was replaced with glucose‐free OGD medium and incubated in a 94% N_2_, 5% CO_2_, and 1% O_2_ incubator for 4 h. After the OGD, the culture medium was returned to glucose‐containing regular medium, and the cells were returned to the Normoxic incubator for reoxygenation.

### 4.20 In Vitro BBB Endothelial Cell Permeability Assay

hCMEC/D3 cells were divided into Normoxia and H/R groups. MCN‐BODIPY was added to the upper chamber at the start of reoxygenation. After 20 h of reoxygenation, 50 µL of the sample was collected from the lower chamber, and fluorescence intensity was measured in a black 96‐well plate. A standard curve was prepared in the same plate (serial dilutions of the nanodrug in culture medium) and calibrated using cell‐free inserts and culture medium blanks. Finally, the fluorescence intensity of MCN‐BODIPY was measured using a microplate reader at an excitation wavelength of 488 nm and an emission wavelength of 525 nm.

### 4.21 TEM of Cerebral Tissue

SD rats were randomly assigned to the Sham‐operated group, I/R group, and MCN treatment group (8 mg/kg). Cerebral tissue was excised in rice‐grain‐sized pieces and fixed in fresh electron micrographic fixative. This was followed by three washes with 0.1 m PBS (pH 7.4), each lasting 15 min. Specimens were then fixed with 1% OsO_4_ at room temperature, protected from light, for 2 h. The specimens were washed three times with PBS for 15 min each. Following dehydration at room temperature, embedding and polymerization were performed. Subsequently, tissue blocks were sectioned into 60–80 nm ultrathin slices using an ultramicrotome. After staining and washing on copper grids, the grids were dried and observed under a TEM for imaging.

### 4.22 Transmission Electron Microscopy

Coated six‐well plates with Glycocalyx, Collagen III, Collagen IV, Collagen III + Quer, and Collagen IV + Quer, then incubated at 4°C for 12 h. Gently rinsed three times with PBS, then added MCN‐BODIPY solution (20 µg/mL) and incubated at 4°C in the dark for 12 h. After thorough rinsing with PBS three times, images were captured under a fluorescence microscope and fluorescence intensity was measured using ImageJ software.

### 4.23 Biological Distribution of MCN

SD rats were randomly assigned to two groups: the Sham‐operated group and the MCN treatment group. A cerebral ischemia model was established using the MCAO method. Following successful modelling, MCN‐BODIPY was administered via sublingual intravenous injection. One, six, and twenty‐four hours post‐administration, euthanasia was performed. The heart, liver, spleen, lungs, kidneys, and cerebral tissues were completely excised and immediately placed in pre‐chilled PBS at 4°C for preservation. Mucous membranes and blood vessels were meticulously dissected from organ surfaces. Using a Leica fluorescence microscope, systematic acquisition of fluorescence signal distribution images was performed across all organs.

### 4.24 Colocalization of Mitochondria and MCN‐BODIPY in HT22 Cells

HT22 cells were seeded at a density of 1 × 10^5^ cells per well in a 12‐well plate. Following cell attachment, cells were incubated with medium supplemented with 400 µm cobalt chloride, MCN‐BODIPY, or MCN‐BODIPY alone. The mitochondrial red fluorescent probe Mitotracker was employed to label HT22 cell mitochondria. Colocalization of MCN‐BODIPY with HT22 cell mitochondria was observed via fluorescence microscopy, and images were captured.

### 4.25 TTC Staining of Cerebral Tissue and Neurological Functional Assessment

#### 4.25.1 TTC Staining

Cerebral tissue was uniformly sliced into 2 mm thick sections. The middle five sections were placed into a six‐well plate. Added 2 mL of 1% TTC solution to each well. Incubated at 37°C in the dark for 15 min, then inverted the plate and continued incubation for a further 15 min. Subsequently, aspirated the TTC solution and added 2 mL of 4% paraformaldehyde to each well for fixation for 24 h. The following day, photographed the stained cerebral tissue sections and imported the images into ImageJ to quantify the infarct area.

#### 4.25.2 ZeaLonga Neurological Functional Scoring

Behavioral scoring was performed on all rats 24 h after reperfusion. 0 points: No neurological deficit; 1 point: Inability to fully extend contralateral forelimb; 2 points: Turning toward paralyzed side; 3 points: Falling toward contralateral side; 4 points: Inability to walk independently, loss of consciousness.

### 4.26 Dose Screening for MCN to Improve CIRI

SD rats were randomly assigned to the Sham‐operated group, the I/R group, and the MCN treatment groups at different doses (4, 8, 12 mg/kg). Each group comprised six SD rats (*n* = 6). Rats in the drug treatment groups received sublingual intravenous administration of varying MCN doses prior to reperfusion initiation. Systemic evaluation was conducted 24 h post‐reperfusion. Neurological deficit severity was quantified using the Zea‐Longa scoring system; cerebral infarct volume was measured via TTC staining, with infarct area percentage calculated.

### 4.27 Efficacy Assessment of MCN in Improving CIRI

SD rats were randomly assigned to the following groups: the Sham‐operated group, the I/R group, the NAC treatment group (8 mg/kg), the Quer treatment group (8 mg/kg), and the MCN treatment group (8 mg/kg). Each group comprised three SD rats (*n* = 3). Rats in the drug treatment groups received sublingual intravenous injections prior to reperfusion initiation. Systemic evaluations were conducted 24 h post‐reperfusion. Neurological deficit severity was quantified using the Zea‐Longa scoring system; cerebral infarct volume was measured via TTC staining, with infarct area percentage calculated.

### 4.28 HE Staining of Cerebral Tissue

SD rats were randomly assigned to the following groups: the Sham‐operated group, the I/R group, the NAC treatment group (8 mg/kg), the Quer treatment group (8 mg/kg), and the MCN treatment group (8 mg/kg). Cerebral tissue from each treatment group was harvested 24 h after ischemia‐reperfusion and fixed in 4% paraformaldehyde for 24 h. The following day, tissues underwent graded dehydration, paraffin embedding, and sectioning (5 µm thickness). Paraffin sections from each group were eluted in xylene and graded ethanol solutions, followed by rehydration in ultrapure water. Hematoxylin staining solution was applied to sections, incubated for 5–10 min, then rinsed to remove excess dye. Differentiation was performed using a 1% hydrochloric acid‐ethanol mixture. After incubation for 10 s, excess dye was rinsed off with pure water. A drop of bluing solution was added, incubated, and then rinsed. Eosin staining solution was added for staining. Subsequently, gradient dehydration was performed in the reverse order of the hydration steps. The sections were immersed in xylene until transparent, mounted with neutral resin, and observed under a microscope.

### 4.29 Nissl Staining of Cerebral Tissue

SD rats were randomly assigned to the following groups: the Sham‐operated group, the I/R group, the NAC treatment group (8 mg/kg), the Quer treatment group (8 mg/kg), and the MCN treatment group (8 mg/kg). After ischemia‐reperfusion of 24 h, cerebral tissue was collected from each treatment group and fixed in 4% paraformaldehyde for 24 h. The following day, tissues underwent stepwise dehydration, paraffin embedding, and sectioning (5 µm thickness). Sections from each treatment group were sequentially immersed in xylene I and xylene II for 10 min each to ensure complete dewaxing. Subsequently, sections were rehydrated by sequential immersion in graded ethanol solutions for 5 min each, followed by rinsing with distilled water. Sections were then immersed in a toluidine blue staining solution and incubated at 37°C for 15 min. Following staining, slides were promptly rinsed with distilled water and transferred to 0.1% glacial acetic acid in ethanol for differentiation. This process was monitored under a microscope until Nissl bodies became clearly visible. Subsequent dehydration was performed using a gradient ethanol series, followed by clearing with xylene for 5 min. Finally, slides were mounted with neutral resin and examined under a microscope.

### 4.30 Modified Neurological Severity Score (mNSS)

The mNSS test was used to evaluate the motor, sensory, reflex, and balance nerve functions of rats in different treatment groups after I/R. The normal score was 0 points, and the maximum functional deficit was 18 points. The higher the score, the more severe the nerve damage.

The mNSS Scoring Criteria for this study are in Table 1.

**TABLE 1:** Scoring Criteria.

Context	Details	Points
Raising rat by the tail (normal score = 0; maximum possible summary score 3)	Flexion of forelimb after raising rat by the tail	1
	Flexion of hindlimb after raising rat by the tail	1
	Head moved >10° to vertical axis within 30 s after raising rat by the tail	1
Placing rat on the floor (normal score = 0; maximum possible summary score = 3)	Normal walk after placing rat on the floor	0
	Inability to walk straight after placing rat on the floor	1
	Circling toward paretic side after placing rat on the floor	2
	Falls down to paretic side after placing rat on the floor	3
Sensory test (normal score = 0; maximum possible summary score = 2)	Placing test (visual and tactile test): take a pen and rotate it at a high frequency in front of the rat's eyes to observe whether there is a blinking reaction; take a face stick and gently scratch the rat's abdomen to observe whether there is an abdominal wall reflex. If there is no blinking reaction, no abdominal wall reflex, or both, add 1 point	1
	Proprioception test: press the mouse paw to the edge of the table to stimulate the limb muscles. If the deep sense is lost, add 1 point	1
Beam and balance test (normal score = 0; maximum possible summary score = 6)	Balances with steady posture	0
	Grasps side of beam	1
	Hugs beam and 1 limb falls down from beam	2
	Hugs beam and 2 limbs fall down from beam, or spins on beam (>60 s)	3
	Attempts to balance on beam, but falls off (>40 s)	4
	Attempts to balance on beam, but falls off (>20 s)	5
	Falls off; no attempt to balance or hang on to beam (<20 s)	6
Reflex absence and abnormal movements test (normal score = 0; maximum possible summary score = 4)	Pinna reflex (shaking head when touching the external auditory canal is normal, and 1 point is added if no shaking head occurs)	1
	Corneal reflex (blinking when cleaning the cornea with cotton wool is normal, and 1 point will be added if no blinking occurs)	1
	Startle reflex (if there is a motor reaction to the noise of the cardboard being bounced quickly, it is normal; if there is no motor reaction, add 1 point.)	1
	Seizure, myoclonus, myodystonia	1

### 4.31 Behavior Tests

In behavioral tests, rats were trained 3 times a day for 5 days before I/R. Tests were performed on days 1, 3, 5, 7, 10, and 14 after I/R.

Adhesive test: This test was used to assess tactile responses and sensorimotor function. A 3 × 3 mm^2^ sticker was placed on the rat's paralyzed front paw. Next, the rats were placed back in their cages, and a timer was started. The time from the beginning until the rat touched the sticker was recorded as the touch time. The time from start to the time the mouse successfully removed the sticker was recorded as the time of removal.

Corner turn test: This test evaluated sensorimotor impairment in rats through unilateral dopaminergic system damage. The rat was placed in a 30‐degree corner formed by two baffles. The operation was repeated 15 times, and the number of times the mouse left the corner by turning left or right was recorded. The percentage of right turns: R/(L + R) × 100(%). Left, L; right, R.

### 4.32 Transcriptome Sequencing

RNA was extracted from cerebral tissue of rats in the Sham‐operated group, the I/R group, and the MCN treatment group. Total RNA was extracted using the standard TRIzol protocol, and cDNA libraries were synthesized via mRNA enrichment processing. Sequencing was performed on a DNBSEQ T7 high‐throughput sequencing platform. The resulting data were subjected to quantitative gene analysis. Differential expression analysis was performed using DESeq2 (with thresholds of |log_2_(fold change)| ≥ 1 and *P* ≤ 0.05). Furthermore, gene functional enrichment analysis and pathway enrichment analysis were conducted on the differentially expressed genes.

### 4.33 Establishment of a Coculture Model of HT22 and BV2 Cells

Using the Transwell co‐culture system, BV2 cells were seeded at a density of 1 × 10^5^ cells per well in the upper chamber and HT22 cells in the lower chamber to establish the co‐culture model, followed by randomized grouping. Following an initial 12 h incubation, the Normoxia group maintained conventional culture conditions. The H/R group and drug‐treated groups were switched to medium containing 400 µm CoCl_2_ (with the drug‐treated group additionally receiving 10 or 20 µg/mL MCN), continuing culture for 16 h to simulate hypoxia. Subsequently, medium containing only the corresponding concentration of MCN was introduced for an 8 h reoxygenation period. Upon experimental termination, HT22 cells from the lower chamber were harvested for WB and qPCR analysis, while BV2 cells from the upper chamber underwent WB analysis. Concurrently, culture medium was collected for inflammatory cytokine level determination.

### 4.34 Western Blotting

Lysis and extraction of cortical tissue from different treatment groups (the Sham‐operated group, the I/R group, the MCN treatment group (8 mg/kg)) cortical tissues and proteins from HT22 and BV2 cells from different treatment groups (Normoxia group, H/R group, H/R+MCN treatment group (10 µg/mL), H/R+MCN treatment group (20 µg/mL)). Proteins were separated by 8%–12% SDS‐PAGE and transferred onto PVDF membranes. After blocking with 5% skimmed milk powder in TBST at room temperature for 1.5 h, membranes were incubated overnight at 4°C with primary antibodies (see Table 2 for antibody details). The following day, PVDF membranes were incubated with goat anti‐rabbit secondary antibody or goat anti‐mouse secondary antibody at room temperature for 1 h. Following thorough washing with TBST, bands were visualized using an enhanced chemiluminescence system. Protein band intensity was measured using ImageJ software.

**TABLE 2:** List of WB antibodies.

Primary/secondary antibody	Dilution	Company	Cat. No
β‐actin	1:10000	Proteintech	66009‐1‐Ig
Bax	1:3000	Abcam	ab32503
Bcl2	1:2000	Affinity	BF9103
Cleaved caspase 3	1:1000	Wanleibio	WL02117
Cytochrome C	1:3000	Proteintech	10993‐1‐AP
cGAS	1:2000	Proteintech	29958‐1‐AP
STING	1:2000	Proteintech	19851‐1‐AP
P65	1:2000	Affinity	BF8005
IRF3	1:1000	Affinity	DF6895
P‐IRF3	1:500	Affinity	AF2436
P‐P65	1:500	Affinity	AF2006
Goat anti‐mouse IgG H&L (HRP)	1:3000	Abclonal	AS003
Goat anti rabbit IgG H&L (HRP)	1:5000	Abclonal	AS014
CHOP/GADD153 polyclonal antibody	1:1000	Proteintech	15204‐1‐AP
Phospho‐EIF2S1 (Ser51) monoclonal antibody	1:5000	Proteintech	68023‐1‐Ig
XBP1S‐specific polyclonal antibody	1:2000	Proteintech	24868‐1‐AP
Caspase 12 polyclonal antibody	1:1000	Proteintech	55238‐1‐AP
EIF2S1 polyclonal antibody	1:5000	Proteintech	11170‐1‐AP
Phospho‐TMEM173/STING (Ser366) antibody	1:500	Affinity	AF7416

### 4.35 CCK8 Assay

#### 4.35.1 Biocompatibility Assessment of MCN

HT22 and BV2 cells were seeded at a density of 1 × 10^4^ cells per well in 96‐well plates. After 24 h, cells were further cultured for 24 or 48 h in medium supplemented with varying concentrations of MCN. Subsequently, cell supernatants were removed, and medium containing CCK8 was added for a further 1‐hour incubation. Cell viability was assessed by measuring absorbance values at 450 nm for each group.

#### 4.35.2 Dose Screening of MCN for Improving H/R‐Induced HT22 Cells

HT22 cells were seeded at a density of 1 × 10^4^ cells per well in 96‐well plates. After 24 h, cells were randomly assigned to the Normoxia group, the H/R group, and FQNZ treatment groups at different concentrations (2.5, 5, 10, 20, 40, and 80 µg/mL). Cell supernatants were removed, and CCK8‐containing medium was added for a further 1‐hour incubation. Cell viability was assessed by measuring absorbance at 450 nm for each group. Cells treated with medium lacking cobalt chloride and MCN served as the Normoxia control group.

### 4.36 DHE Staining of Cerebral Tissue

SD rats were randomly assigned to the Sham‐operated group, the I/R group, and the MCN treatment group (8 mg/kg). Cerebral tissue from each treatment group was harvested 24 h after ischemia‐reperfusion and fixed in 4% paraformaldehyde for 24 h. The following day, tissues underwent graded dehydration, paraffin embedding, and sectioning (5 µm thickness). Tissue ROS staining was performed using the DHE probe. Cerebral tissue sections from different treatment groups were dewaxed and washed, followed by heat‐induced reactivation with 1 × EDTA. After cooling, sections were washed three times with PBS. DHE (10 µm) was then applied to the sections and incubated for 30 min. Following washing, sections were incubated with DAPI solution at room temperature for 10 min. After three washes with PBS, an anti‐fluorescence quencher was applied, coverslips were mounted, and observations were made using an upright fluorescence microscope.

### 4.37 Immunofluorescence

Cerebral tissue sections from different treatment groups (the Sham‐operated group, the I/R group, the MCN‐treated group (8 mg/kg)) or cell monolayer sheets (Normoxia group, H/R group, H/R+MCN‐treated group (10 µg/mL), H/R+MCN treatment group (20 µg/mL)) were incubated with primary antibodies (specific antibody details in Table 3) at 4°C for 16 h. Following two washes with PBS buffer (6 min each), the slides were incubated with the corresponding secondary antibody at 37°C for 1 h. Following secondary antibody incubation, samples were washed twice with PBS (6 min each). Finally, coverslips were sealed with DAPI‐containing anti‐fluorescence quencher. Images were acquired using a Leica fluorescence microscope and analyzed via ImageJ software.

**TABLE 3:** List of immunofluorescence staining antibodies.

Primary/secondary antibody	Company	Cat. No	Dilution
iNOS antibody	Affinity	AF0199	1:100
CD206 recombinant antibody	Proteintech	81525‐1‐RR	1:100
Anti‐dsDNA antibody	Abcam	AB27156	1:500
TOM20 polyclonal antibody	Proteintech	11802‐1‐AP	1:500
Goat anti‐rabbit IgG (H+L) cross‐adsorbed secondary antibody, Alexa Fluor 555	Invitrogen	A21428	1:500
Goat anti‐mouse IgG (H+L) highly cross‐adsorbed secondary antibody, Alexa Fluor 488	Invitrogen	A11029	1:500

### 4.38 Inflammatory Factor Detection

Cerebral tissue samples were collected from the Sham‐operated group, the I/R group, and the MCN treatment group (8 mg/kg). Following PBS washing, 10% homogenates were prepared and centrifuged at 15 000 rpm for 15 min to obtain the supernatant. Additionally, conditioned microglial culture media were collected from the Normoxia group, H/R group, H/R+MCN treatment group (10 µg/mL), and H/R+MCN treatment group (20 µg/mL). Using the ELISA kits specified in Tables 4 and 5, the expression levels of pro‐inflammatory and anti‐inflammatory factors were quantitatively detected in the cerebral tissue homogenate supernatants and cell culture media, respectively.

**TABLE 4:** List of ELISA kits for tissue.

Primary/secondary antibody	Company	Cat. No
Rat IL‐1β (Interleukin 1 beta) ELISA kit	Elabscience	E‐EL‐R0012
Rat IL‐6 (Interleukin 6) ELISA kit	Elabscience	E‐EL‐R0015
Rat TNF‐α (tumor necrosis factor alpha) ELISA kit	Elabscience	E‐EL‐R2856
Rat IL‐4 (Interleukin 4) ELISA kit	Elabscience	E‐EL‐R0015
Rat IL‐10 (Interleukin 10) ELISA kit	Elabscience	E‐EL‐R0016
Rat IFN‐β (Interferon beta) ELISA kit	Elabscience	E‐EL‐R0545

**TABLE 5:** List of ELISA kits for BV2 cells.

Primary/secondary antibody	Company	Cat. No
Mouse IL‐1β (Interleukin 1 beta) ELISA kit	Elabscience	E‐EL‐M0037
Mouse IL‐6 (Interleukin 6) ELISA kit	Elabscience	E‐EL‐M0044
Mouse TNF‐α (tumor necrosis factor alpha) ELISA kit	Elabscience	E‐EL‐M3063
Mouse IL‐4 (Interleukin 4) ELISA kit	Elabscience	E‐EL‐M0043
Mouse IL‐10 (Interleukin 10) ELISA kit	Elabscience	E‐EL‐M0046
Mouse IFN‐β(Interferon Beta) ELISA kit	Elabscience	E‐EL‐M0033

### 4.39 Copy Number of mtDNA

To quantify mtDNA leakage from mitochondria into the cytoplasm, cytoplasmic and mitochondrial fractions were isolated from cells using a mitochondrial DNA extraction kit (Phygene Biotechnology PH1592) according to the manufacturer's instructions. DNA was extracted from each fraction using a DNA extraction kit (Yeasen, 18700ES50). QPCR was performed using TB Green Premix Ex Taq (Takara, Japan) on a qPCR system. Primers targeting the mitochondrial gene MT‐ND1 were used to detect mitochondrial DNA, while 18S rDNA was used as the nuclear reference gene. The relative copy number of mtDNA in each fraction was calculated using the 2^−ΔΔCt^ method. All samples were analyzed in triplicate.

### 4.40 Quantitative Real‐Time Polymerase Chain Reaction (qPCR)

Total RNA was extracted from cerebral tissue samples of the Sham‐operated group, the I/R group, and the MCN treatment group (8 mg/kg), as well as HT22 cells from the Normoxia group, H/R group, H/R+MCN treatment group (10 µg/mL), and H/R+MCN treatment group (20 µg/mL) using the TRIzol method. Following reverse transcription to obtain cDNA, qPCR analysis was performed using the TB Green chimeric fluorescence method. Gene expression levels were quantified by Ct values and normalized against β‐actin as the internal reference gene.

The qPCR primers used in this study are as follows (Tables 6 and 7):

**TABLE 6:** List of qPCR primers for tissue.

Gene name	Primer sequence (5’‐3’)
BiP‐F	TAC TCG AAT TCC AAA GAT TCA G
BiP‐R	TCA AGC AGA ACC AGG TC
PERK‐F	TCCTGTCTTGGTTGGGTCTG
PERK‐R	TGCGTGCTCCGCTTATTC
ATF_6‐F	GGATTTGATGCCTTGGGAGTCAGAC
ATF_6‐R	ATTTTTTTCTTTGGAGTCAGTCCAT
IRE1‐F	AGAGCCCATCACCTTGCTT
IRE1‐R	TGATCCTGCCATGTGCGTT
eIF2α‐F	TTATGCCTGCGAAAGCAAC
eIF2α‐R	TTCCATTTGTCCTCGAAGGT
ATF4‐F	GCCAAGCACTTCAAACCTCA
ATF4‐R	GCATGGTTTCCAGGTCATCC
XBP1s‐F	CTGAGTCCGCAGCAGGTG
XBP1s‐R	GACCTCTGGGAGTTCCTCCA
JNK‐F	AGTGTAGAGTGGATGCATGA
JNK‐R	ATGTGCTTCCTGTGGTTTAC
Caspase‐12‐F	ATAGCCACTGCTGATACAGA
Caspase‐12‐R	CCACTCTTGCCTACCTTCC
CHOP‐F	CCTGAAAGCAGAAACCGGTC
CHOP‐R	CCTCATACCAGGCTTCCAGC
β‐actin‐F	CACTGCCGCATCCTCTTCCT
β‐actin‐R	AACCGCTCATTGCCGATAGTG
INOS‐F	TCCTCAGGCTTGGGTCTTGT
INOS‐R	ATCCTGTGTTGTTGGGCTGG
CD206‐F	TCAACTCTTGGACTCACGGC
CD206‐R	CATGATCTGCGACTCCGACA
CD86‐F	AGACATGTGTAACCTGCACCAT
CD86‐R	TACGAGCTCACTCGGGCTTA
Arg1‐F	CCAGTATTCACCCCGGCTAC
Arg1‐R	GTCCTGAAAGTAGCCCTGTCT
Sod2‐F	GCAAGGAACCACAGGCCTTA
Sod‐2‐R	TGCTCCCACACATCAATCCC
Gpx1‐F	ATCAGTTCGGACATCAGGAGA
Gpx1‐R	TCACCATTCACCTCGCACTT
Atp5pd‐F	CTGTCCCGGGTGGTACTTTC
Atp5pd‐R	GCCTGTAAAACCCCTGGACC
Cycs‐F	CCAGCCCGGACCGAATTTA
Cycs‐R	CTGTCTTCCGCCCAAACAGA
IL‐1β‐F	CCA GGA TGA GGA CCC AAG CA
IL‐1β‐R	TCC CGA CCA TTG CTG TTT CC
IL‐4‐F	TGC ACC GAG ATG TTT GTA CC
IL‐4‐R	GGA TGC TTT TTA GGC TTT CC
IL‐10‐F	GCA GGA CTT TAA GGG TTA CTT GG
IL‐10‐R	GGG GAG AAA TCG ATG ACA GC
IL‐6‐F	TCC TAC CCC AAC TTC CAA TGC TC
IL‐6‐R	TTG GAT GGT CTT GGT CCT TAG CC

**TABLE 7:** List of qPCR primers for HT22 cells.

Gene name	Primer sequence (5’‐3’)
BiP‐F	TGTGTGTGAGACCAGAACCG
BiP‐R	TAGGTGGTCCCCAAGTCGAT
PERK‐F	AGGCTTTAACTTCCCGCATT
PERK‐R	AGTGCCAGACTGAAAGTAAATACG
ATF‐6‐F	TCGCCTTTTAGTCCGGTTCTT
ATF‐6‐R	GGCTCCATAGGTCTGACTC
IRE1‐F	CCTACAAGAGTATGTGGAGC
IRE1‐R	GGTCTCTGTGAACAATGTTGAGAG
eIF2α‐F	CCGCTCTTGACAGTCCGAG
eIF2α‐R	GCAGTAGTCCCTTGTTAGTGACA
ATF4‐F	CCTGAACAGCGAAGTGTTGG
ATF4‐R	TGGAGAACCCATGAGGTT
XBP1s‐F	CTGAGTCCGAATCAGGTGCAG
XBP1s‐R	GTCCATGGGAAGATGTTCTGG
JNK‐F	GGTATGCCCAAGAGGACAGAGGA
JNK‐R	AGCCCAGATAGAGCCAGTCGTAA
Caspase‐12‐F	ATGGCGGCCAGGAGGACACATG
Caspase‐12‐R	CTAATTCCCGGGAAAAAGGTAG
CHOP‐F	GTCCCTGCCTTTCACCTTGG
CHOP‐R	GGTTTTTGATTCTTCCTCTTCG
β‐actin‐F	ACGAGGCCCAGAGCAAGA
β‐actin‐R	TTGGTTACAATGCCGTGTTCA
ND1‐F	CTAGCAGAAACAAACCGGGC
ND1‐R	CCGGCTGCGTATTCTACGTT
IL‐6‐F	TCCAGAAACCGCTATGAAGTTC
IL‐6‐R	CACCAGCATCAGTCCCAAGA
IL10‐F	TGAATTCCCTGGGTGAGAAGCTGA
IL10‐R	TGGCCTTGTAGACACCTTGGTCTT
IL4‐F	TTGGAAGCCCTACAGACGAG
IL4‐R	TTGAGCAGATGACATTGGGGC
TNF‐α‐F	AGAGGCACTCCCCCAAAAGA
TNF‐α‐R	CGATCACCCCGAAGTTCAGT
18S rDNA‐F	AGGGGAGAGCGGGTAAGAGA
18S rDNA‐R	GGACAGGACTAGGCGGAACA
Sod2‐F	GGAGCAAGGTCGCTTACAGA
Sod‐2‐R	GCGGAATAAGGCCTGTTGTT
Gpx1‐F	TCAGTTCGGACACCAGAATGG
Gpx1‐R	GGAAGGTAAAGAGCGGGTGA
Atp5pd‐F	CCTTGTGGCTTGAGAGATGGTA
Atp5pd‐R	CAGCCCAAGACGCACTTTTC
Cycs‐F	ACCAGCCCGGAACGAATTAAA
Cycs‐R	CCGAACAGACCGTGGAGATT
IL‐1β‐F	GGCTTCCTTGTGCAAGTGTC
IL‐1β‐R	AGTCAAGGGCTTGGAAGCAA

### 4.41 Immunohistochemistry

Paraffin sections of cerebral tissue from different treatment groups (the Sham‐operated group, the I/R group, the MCN treatment group (8 mg/kg)) underwent deparaffinization and rehydration, followed by the following sequential steps: Following antigen retrieval with EDTA buffer, endogenous peroxidase activity was inactivated. Sections were blocked with 5% BSA and incubated overnight at 4°C with cGAS/STING primary antibody. The following day, the reaction sequence comprised: addition of reaction enhancer, incubation with enzyme‐labelled secondary antibody (30 min), and DAB color development. Reactions were terminated by counterstaining with hematoxylin, followed by differentiation with hydrochloric acid ethanol and counterstaining with tap water. Slides were then dehydrated using a gradient ethanol series, cleared with xylene, and mounted with neutral resin. Final immunohistochemical images were observed and captured under an optical microscope.

The antibodies used for immunohistochemical staining in this study are as follows (Table 8):

**TABLE 8:** Antibody list 2.

Primary/secondary antibody	Company	Cat. No	Dilution
cGAS	Proteintech	29958‐1‐AP	1:500
STING	Proteintech	19851‐1‐AP	1:500

### 4.42 Intracellular ROS Level Assay in HT22 Cells

HT22 cells were seeded at 1 × 10^5^ cells per well in a 6‐well plate and incubated for 24 h. They were then randomly assigned to different treatment groups: Normoxia group, H/R group, H/R + MCN treatment group (10 µg/mL), and H/R + MCN treatment group (20 µg/mL). Subsequently, cells in each treatment group were washed three times with PBS. The DCFH‐DA fluorescent probe was added, and the mixture was incubated at 37°C in the dark for 30 min. Cells were then washed three times with PBS to remove residual fluorescent probe. Finally, images of cells in each treatment group were captured under a fluorescence microscope. The experiment was repeated three times, and statistical analysis was performed.

### 4.43 Intracellular Mitosox Level Assay in HT22 Cells

HT22 cells were seeded at 1 × 10^5^ cells per well in a 6‐well plate and incubated for 24 h. They were then randomly assigned to different treatment groups: Normoxia group, H/R group, H/R + MCN treatment group (10 µg/mL), and H/R + MCN treatment group (20 µg/mL). Cells in each treatment group were washed three times with HBSS, followed by the addition of MitoSOX Red fluorescent probe (incubated at 37°C in the dark for 15 min). Residual probe was then removed by washing three times with HBSS. Subsequently, Hoechst working solution was added (incubated at 37°C in the dark for 10 min). After two washes with HBSS, images were acquired under a fluorescence microscope. All experiments were independently repeated three times, and data underwent statistical analysis.

### 4.44 Intracellular MMP Detection in HT22 Cells

HT22 cells were seeded at 1 × 10^5^ cells per well in a 6‐well plate and incubated for 24 h. They were then randomly assigned to different treatment groups: Normoxia group, H/R group, H/R + MCN treatment group (10 µg/mL), and H/R + MCN treatment group (20 µg/mL). Following washing with buffer solution, cells in each group were incubated with JC‐1 probe at 37°C in the dark for 16 mins. After washing, MMP changes were assessed using fluorescence microscopy (via red/green fluorescence intensity ratio) and Cytek DxP Athena flow cytometer (model 01‐DXPSF13‐01). All experiments were independently replicated three times to ensure data reliability.

### 4.45 Intracellular ATP Level Assay in HT22 Cells

HT22 cells were seeded at 1 × 10^5^ cells per well in a 6‐well plate and incubated for 24 h. They were then randomly assigned to different treatment groups: Normoxia group, H/R group, H/R + MCN treatment group (10 µg/mL), and H/R + MCN treatment group (20 µg/mL). Cells from each treatment group were harvested, washed once with pre‐chilled PBS, and thoroughly lysed with RIPA buffer before transfer to centrifuge tubes. Centrifugation at 4°C and 12 000 g for 5 min yielded the supernatant for subsequent use. ATP content in each group was subsequently measured using the BiYunTian Enhanced ATP Detection Kit. All experiments were independently replicated three times, with data subjected to statistical analysis.

### 4.46 TUNEL Staining of Cerebral Tissue

Paraffin sections of cerebral tissue from different treatment groups (the Sham‐operated group, the I/R group, the MCN treatment group (8 mg/kg)) underwent dewaxing and rehydration. Following the TUNEL assay kit protocol (YESSEN, 40306ES50), sections were stained, with cell nuclei subsequently stained using DAPI (Solarbio, C0060). Finally, cells were observed under a fluorescence microscope.

### 4.47 Cell Annexin V‐FITC/PI Analysis

HT22 cells were seeded at 1 × 10^5^ cells per well in 6‐well plates and incubated for 24 h. They were then randomly assigned to different treatment groups: Normoxia group, H/R group, H/R + MCN treatment group (10 µg/mL), and H/R + MCN treatment group (20 µg/mL). Cells from each treatment group were harvested and processed according to the Annexin V‐FITC/PI reagent protocol. Apoptosis in each group was analyzed using flow cytometry (Cytek, DxpAthena, 01‐DXPSF13‐01). The experiment was repeated three times, and statistical analysis was performed.

### 4.48 Biocompatibility Assessment

SD rats were randomly assigned to the Sham‐operated group and the MCN treatment group (*n* = 3), receiving equivalent doses of MCN or physiological saline. Rats were euthanized at 24 h and 28 days post‐treatment. Histological examination of rat brain, heart, liver, spleen, lung, and kidney tissues was conducted using HE staining. Complete blood count parameters were analyzed using whole blood methods. Liver function (alanine aminotransferase (ALT) and gamma‐glutamyltransferase (γ‐GT)) and renal function (blood urea nitrogen (BUN) and creatinine (CR)) parameters were assessed using the fully automated biochemical analyzer BS‐2000M.

### 4.49 Statistical Analysis

All experimental data were presented as mean ± SE, with no experimental samples or data points excluded. In vitro experiments were conducted with three independent replicates (*n* = 3), whilst in vivo experiments comprised at least three animals per group (*n *≥ 3). Statistical analysis employed the following methods: unpaired two‐tailed *t‐*tests for comparisons between two groups, and one‐way analysis of variance (ANOVA) for comparisons involving multiple groups. Specific statistical methods are detailed in the corresponding figure legends, with significance set at *P* < 0.05. Data analysis was performed using statistical software including Origin 2021 (OriginLab Corporation, USA), ImageJ 1.8.0 (National Institutes of Health, USA), and GraphPad Prism 8.0 (GraphPad Software, San Diego, USA).

## Author Contributions

**Jinwen Ge**: methodology, software. **Zhicheng Wang**: formal analysis, validation, visualization, writing – original draft. **Shuya Wang**: formal analysis, validation. **Wenxuan Zheng**: investigation, validation, writing – original draft, methodology, writing – review and editing. **Xin Zhou**: investigation, validation, writing – review and editing. **Chong Liu**: funding acquisition, visualization, project administration, supervision, conceptualization, writing – review and editing, writing – original draft. **Zhen Chen**: methodology. **Yuting Lin**: methodology, investigation. **Ruishi Li**: methodology, investigation. **Tingli Xiong**: investigation, methodology. **Xiaojing Shi**: investigation, validation. **Jiawen Wei**: methodology. **Fei Li**: methodology. **Kelong Ai**: project administration, supervision, conceptualization. **Guiming Deng**: funding acquisition, project administration, conceptualization, data curation, resources.

## Conflicts of Interest

The authors declare no conflicts of interest.

## Supporting information




**Supporting File**: advs76038‐sup‐0001‐SuppMat.doc.

## Data Availability

The data that supports the findings of this study are available in the supplementary material of this article.
