# Supplementary material for: Intrinsically Mitochondria‐Targeting Nanozyme via Coordination‐Assembly of Natural Quercetin for Cascade Antioxidant Therapy of Cerebral Ischemia‐Reperfusion Injury
Source: Adv Sci (Weinh). 2026 Jun 11:e76038. Online ahead of print. doi: 10.1002/advs.76038 (PMC13336828; doi:10.1002/advs.76038)
Supplement: Supplementary file 1 — Supporting File: advs76038‐sup‐0001‐SuppMat.doc. [file ADVS-9999-e76038-s001.doc]

Supporting Information

**Intrinsically Mitochondria-Targeting Nanozyme via Coordination-Assembly of Natural Quercetin for Cascade Antioxidant Therapy of Cerebral Ischemia-reperfusion Injury**

*Wenxuan Zheng^1,3^*^†^*, Zhicheng Wang^1,2^*^†^, *Xin Zhou^1,6^, Shuya Wang^3^, Xiaojing Shi^3^, Tingli Xiong^3^, Ruishi Li^3^, Yuting Lin^3^, Zhen Chen^2^ Jiawen Wei^3^, Fei Li^3^, Jinwen Ge^5^, Kelong Ai^3,4^, Chong Liu^3*^, Guiming Deng^1,2*^*

^1^The Second Affiliated Hospital of Hunan University of Chinese Medicine, Changsha, 410005, China.

^2^The First Affiliated Hospital of Hunan University of Chinese Medicine, Changsha, 410007, China.

^3^Xiangya School of Pharmaceutical Sciences, Central South University, Changsha, 410013, China.

^4^Hunan Provincial Key Laboratory of Cardiovascular Research, Xiangya School of Pharmaceutical Sciences, Central South University, Changsha, 410013, China.

^5^Hunan Academy of Chinese Medicine, Changsha, 410013, China.

^6^Chongqing Jiulongpo Traditional Chinese Medicine Hospital, Chongqing, 400050, China.

†The authors Wenxuan Zheng and Zhen Chen contributed equally to the work.

***Corresponding authors**: llchong@csu.edu.cn(Prof. Chong Liu), [dengguiming@hnucm.edu.cn (Prof. Guiming](mailto:dengguiming@hnucm.edu.cn(Guiming) Deng)

Experimental data


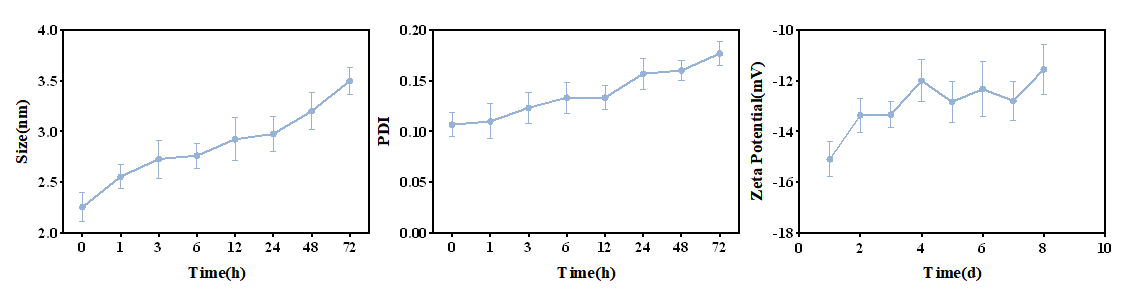


**Figure S1 Colloidal stability of MCN in simulated physiological fluid.** Data were expressed as mean ± SE (in three independent experiments).


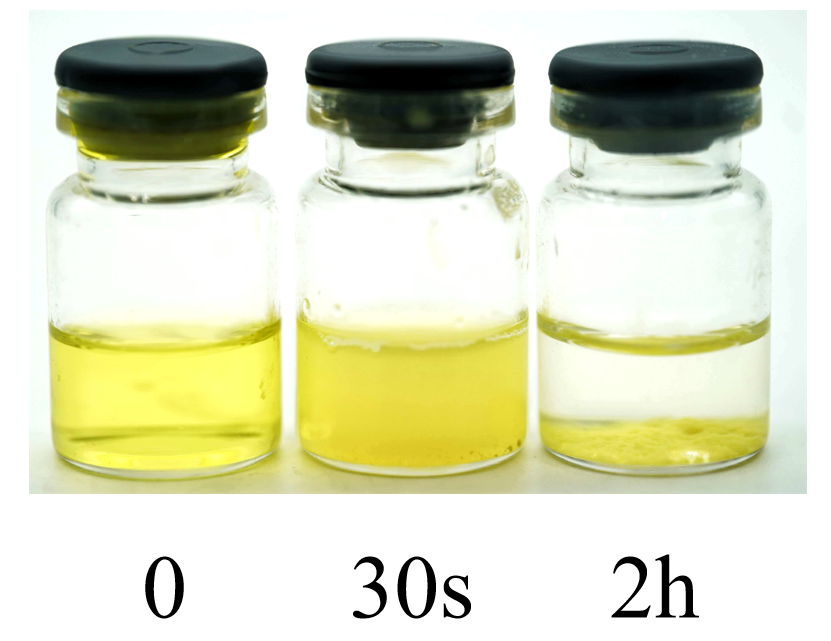

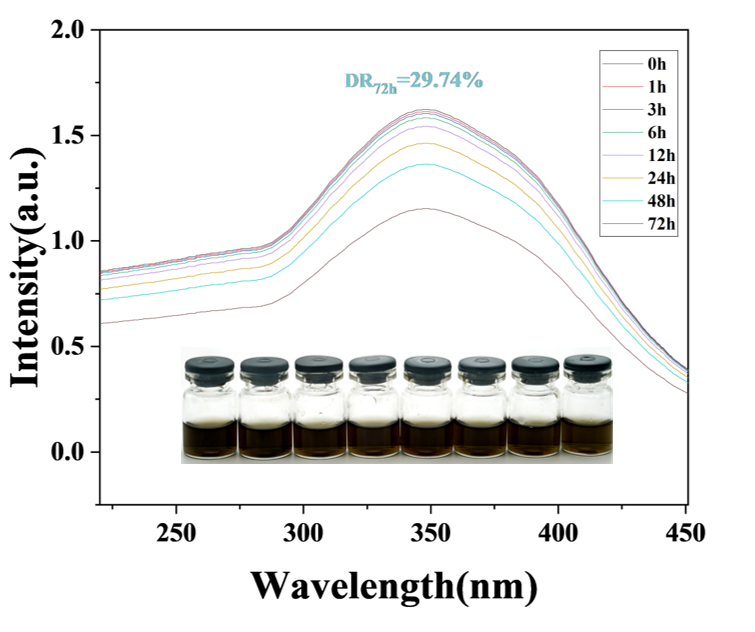


Figure S2 Degradation behavior of Quer (left) and MCN (right) in simulated physiological fluid.


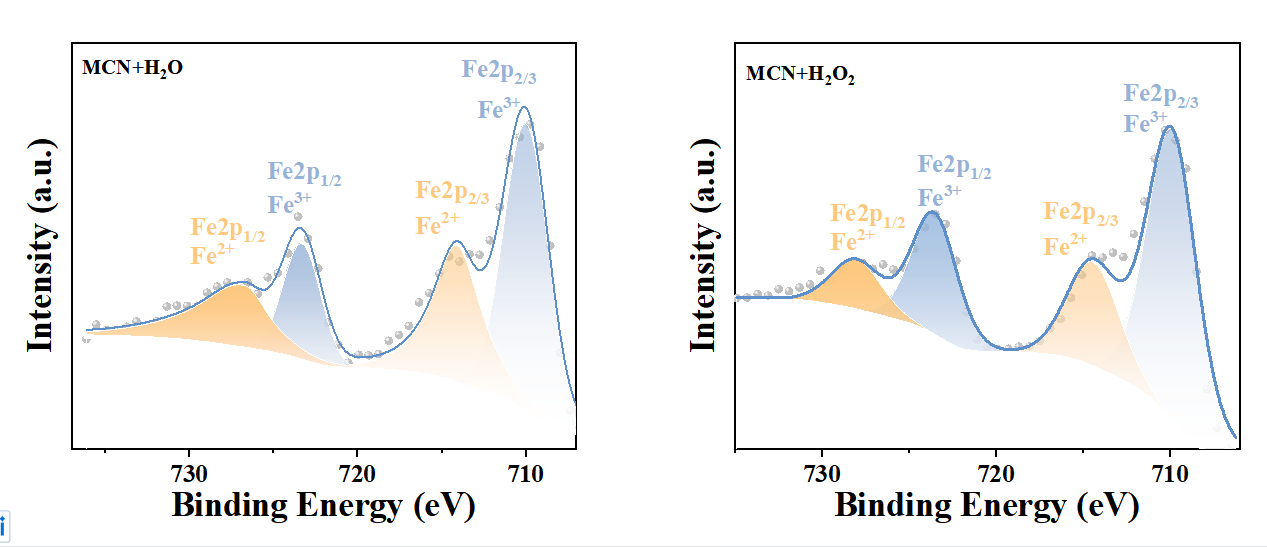


Figure S3 Narrow-scan XPS spectrum of Fe^2+^/Fe^3+^ for MCN with H_2_O (left) and H_2_O_2_ (right).


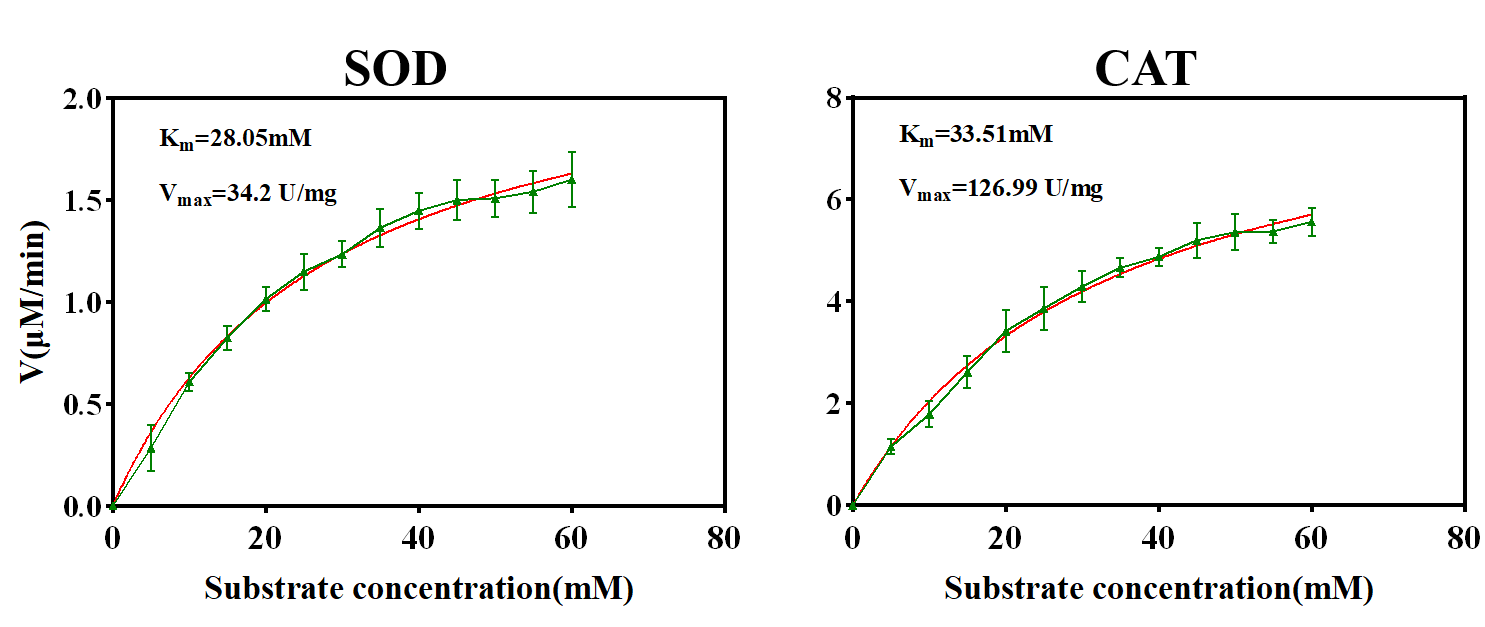


**Figure S4 Kinetic parameters of SOD-like and CAT-like activities of MCN.** Data were expressed as mean ± SE (in three independent experiments).


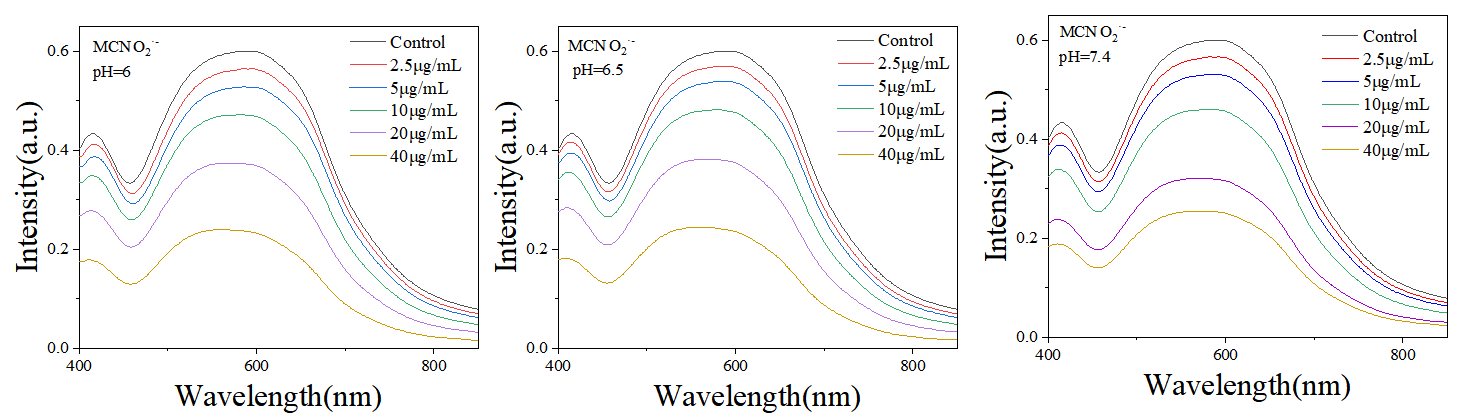


Figure S5 Generation and scavenging ability of O_2_^·-^ by MCN at pH=6 (left) pH=6.5 (center) pH=7.4(right).


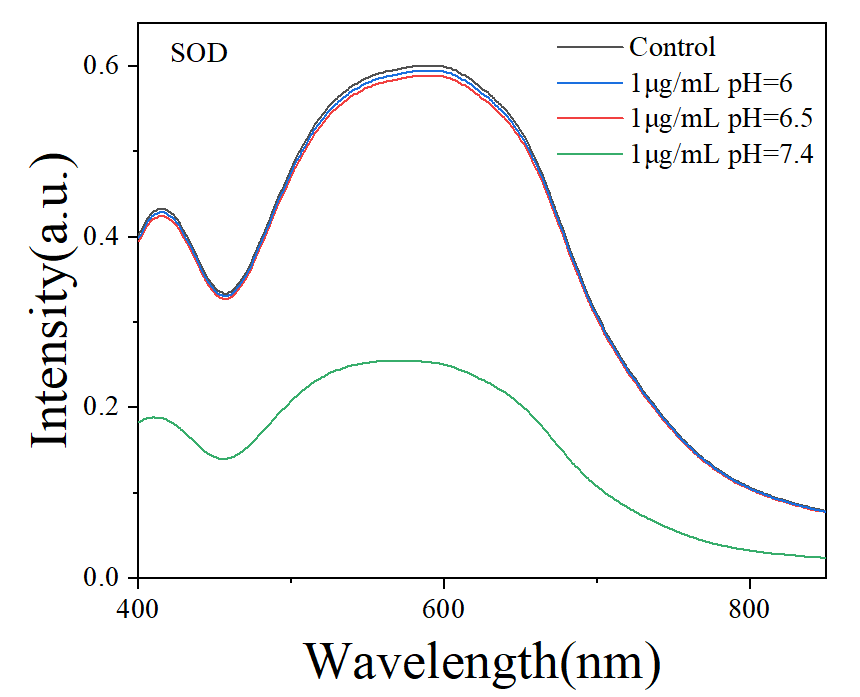


Figure S6 Generation and scavenging ability of O_2_^·-^ by SOD at different pH.


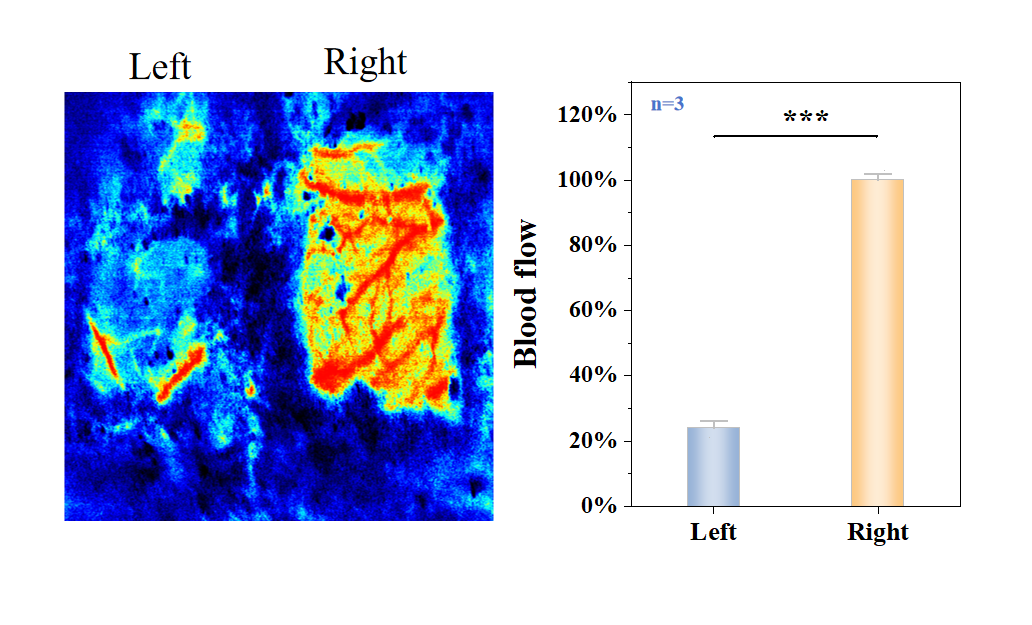


**Figure S7 Representative images (left) and statistics (right) of Verification of MCAO model by laser Doppler flowmetry monitoring of cerebral blood flow in rats.** Data were expressed as mean ± SE (in three independent experiments). Statistical significance was performed by one-way ANOVA with Tukey post hoc test. ns: *P* > 0.05, ^*^*P* < 0.05, ^**^*P* < 0.01, ^***^*P* < 0.001.


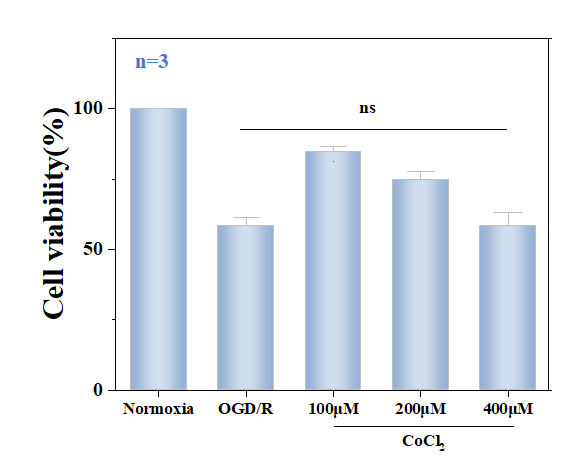


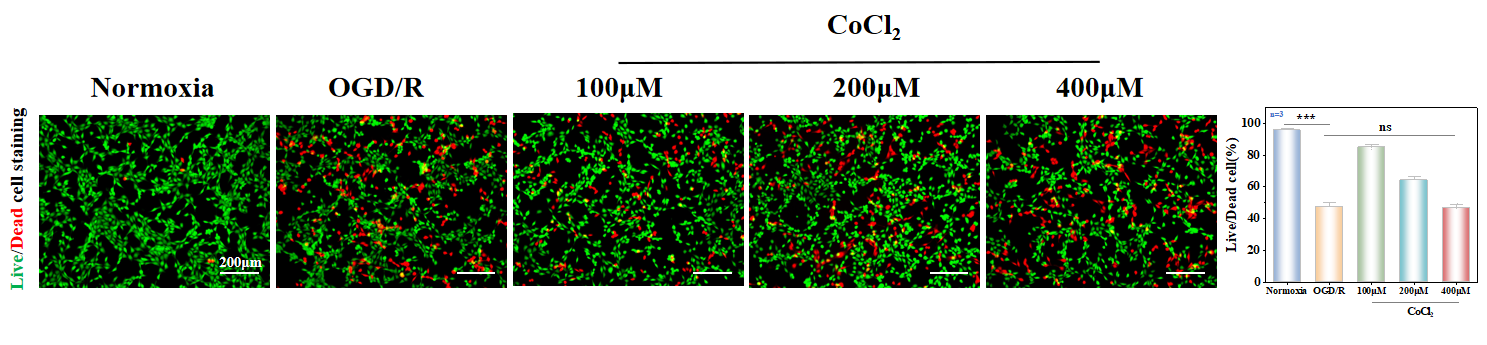


**Figure S8 Validation of the CoCl_2_-induced H/R model mimicking OGD/R injury in HT22 cells by CCK-8 (above) assay and live/dead staining (below).** Data were expressed as mean ± SE (in three independent experiments). Statistical significance was performed by one-way ANOVA with Tukey post hoc test. ns: *P* > 0.05, ^*^*P* < 0.05, ^**^*P* < 0.01, ^***^*P* < 0.001.


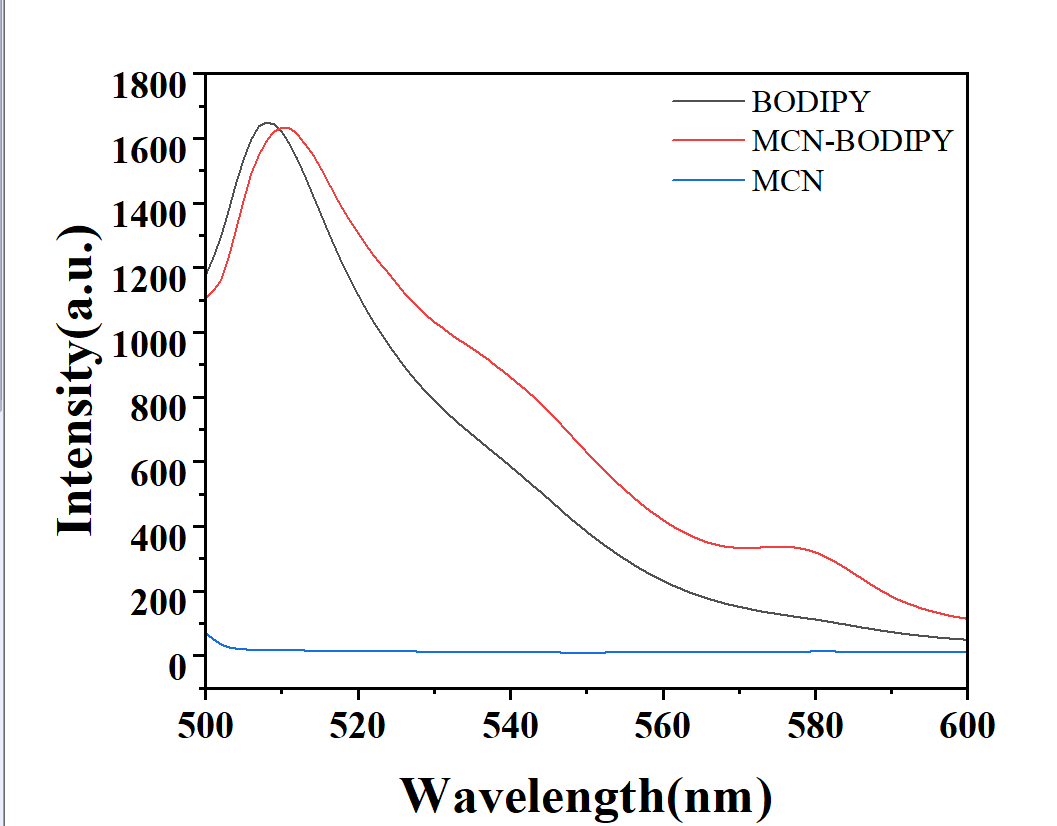


**Figure S9 *In vitro* fluorescence of MCN-BODIPY, BODIPY, MCN.**


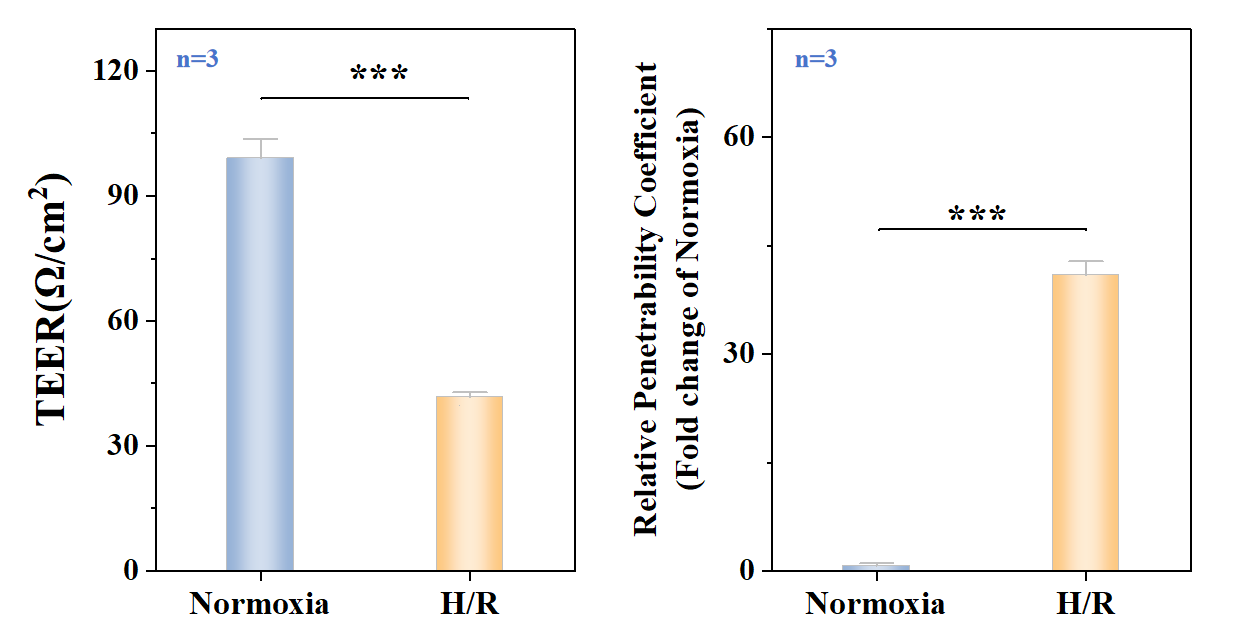


**Figure S10 TEER of hCMEC/D3 monolayer in different treatment groups (left). Relative permeability of MCN-BODIPY in different treatment groups (right).** Data were expressed as mean ± SE (in three independent experiments). Statistical significance was performed by one-way ANOVA with Tukey post hoc test. ns: *P* > 0.05, ^*^*P* < 0.05, ^**^*P* < 0.01, ^***^*P* < 0.001.


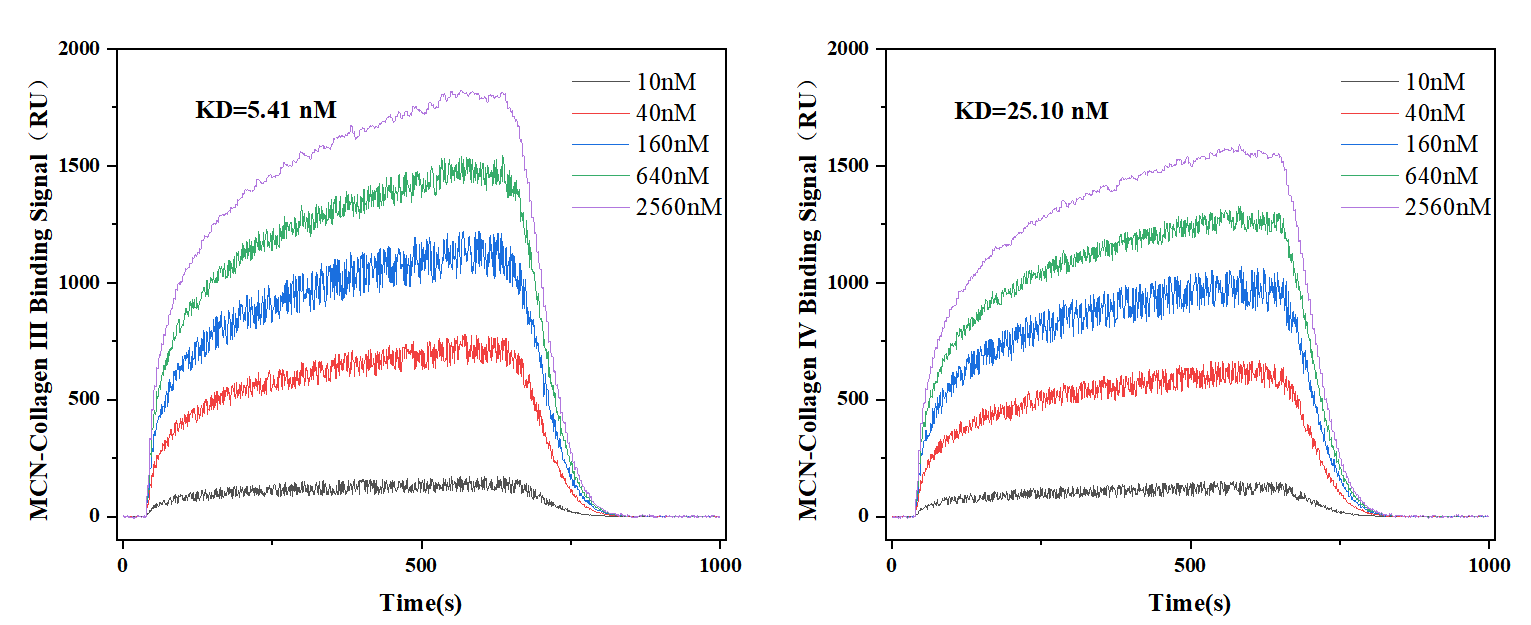


**Figure S11 SPR analysis of the binding affinity between MCN and collagen III (left) or collagen IV (right).**


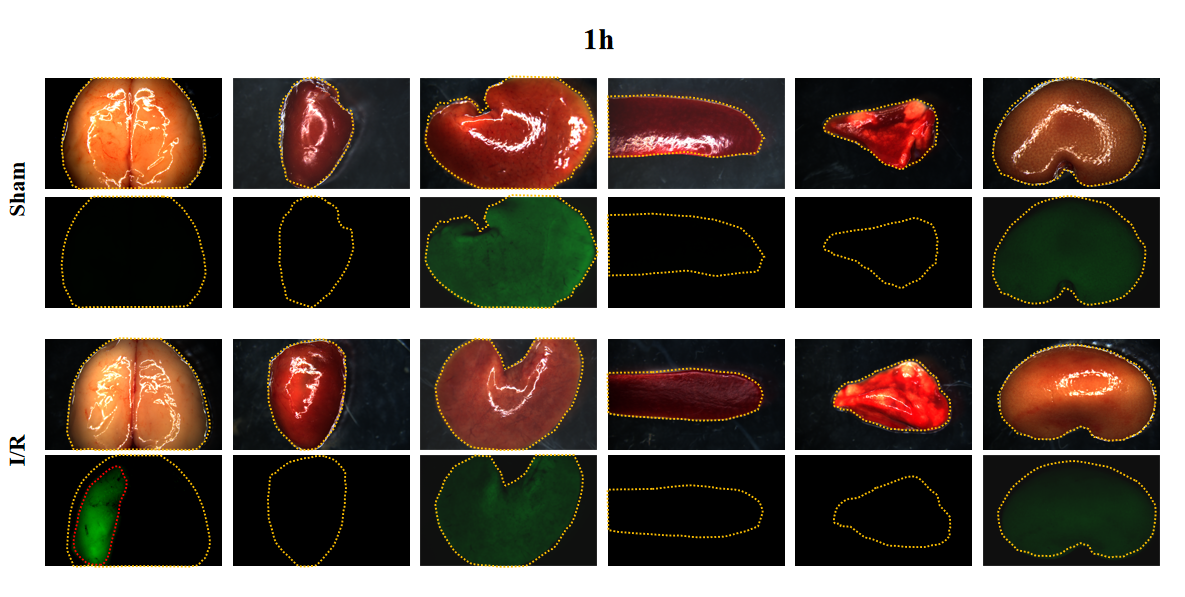


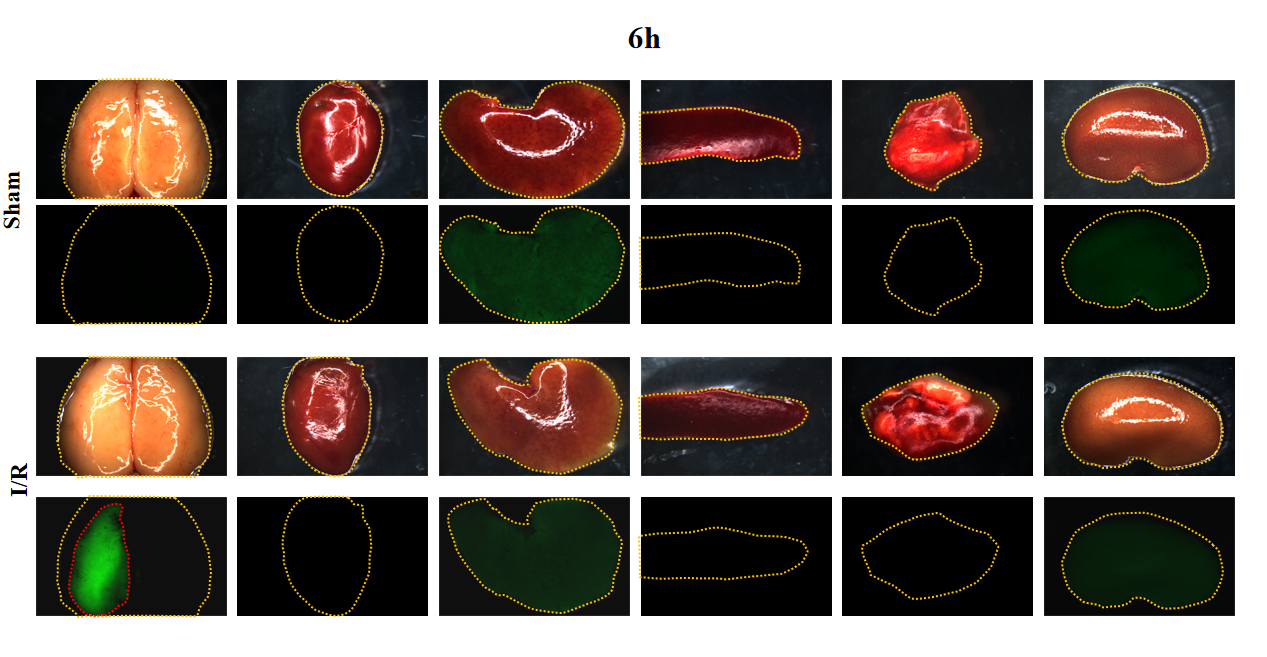


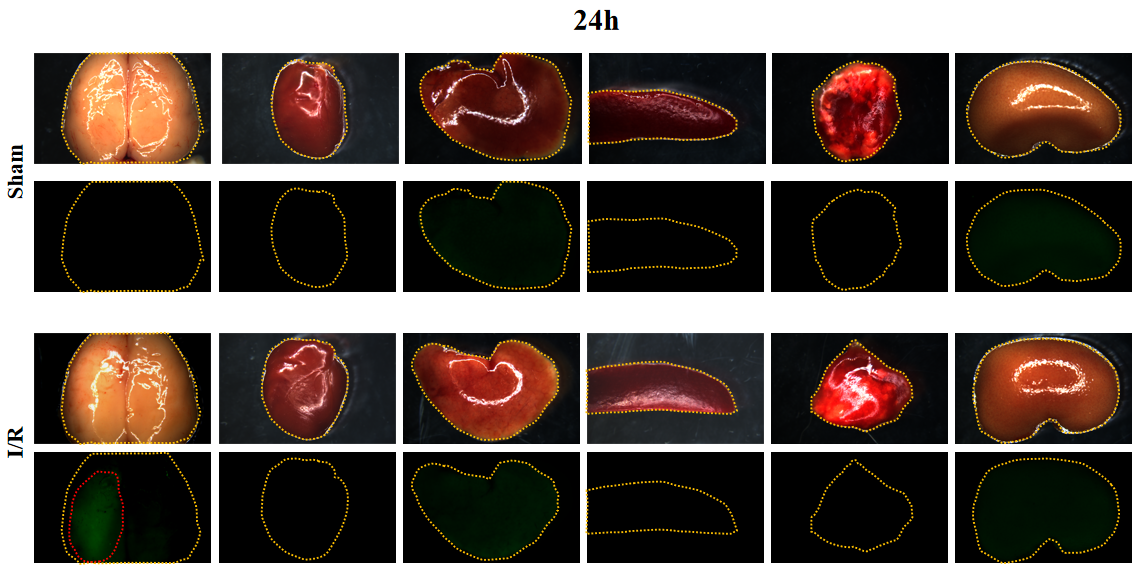


**Figure S12 Representative images of fluorescence imaging in major organs (brain, heart, liver, spleen, lung, and kidney) of rats in the Sham and I/R group 1 h, 6 h, 24 h after sublingual intravenous injection of MCN-BODIPY.** Data were expressed as mean ± SE (in three independent experiments).


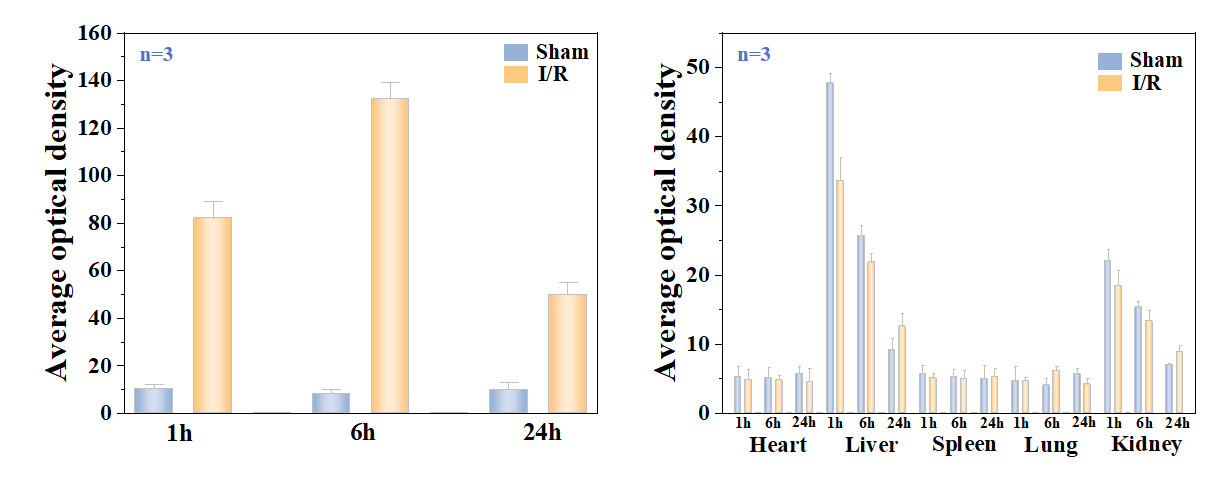


**Figure S13 Statistics of fluorescence intensity in brain (left), and other major organs (right) of rats in the Sham and I/R group 1 h, 6 h, 24 h after sublingual intravenous injection of MCN-BODIPY.** Data were expressed as mean ± SE (in three independent experiments). Statistical significance was performed by one-way ANOVA with Tukey post hoc test. ns: *P* > 0.05, ^*^*P* < 0.05, ^**^*P* < 0.01, ^***^*P* < 0.001.


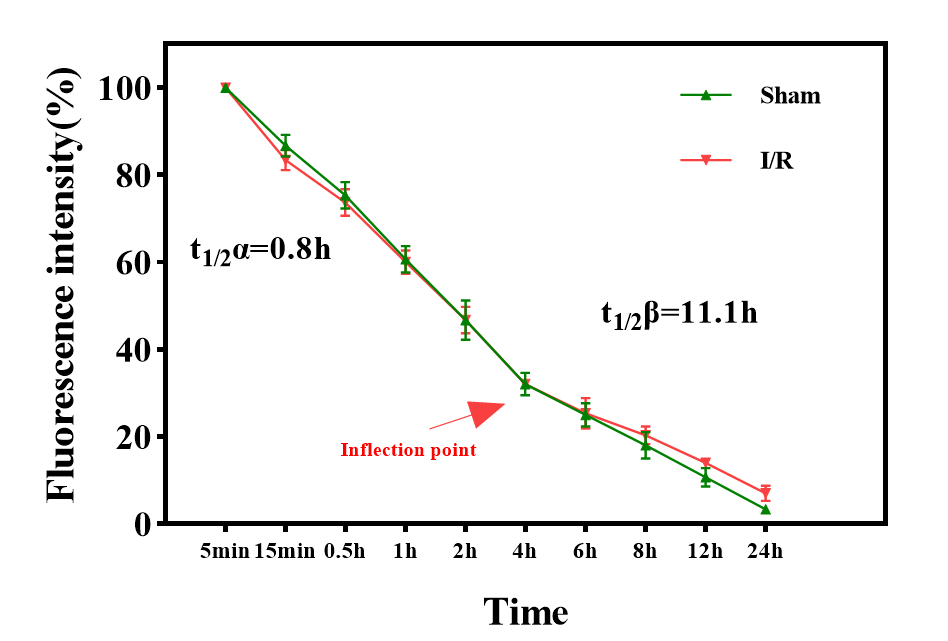


**Figure S14 Plasma concentration-time curve of MCN-BODIPY in different groups.** Data were expressed as mean ± SE (in three independent experiments).


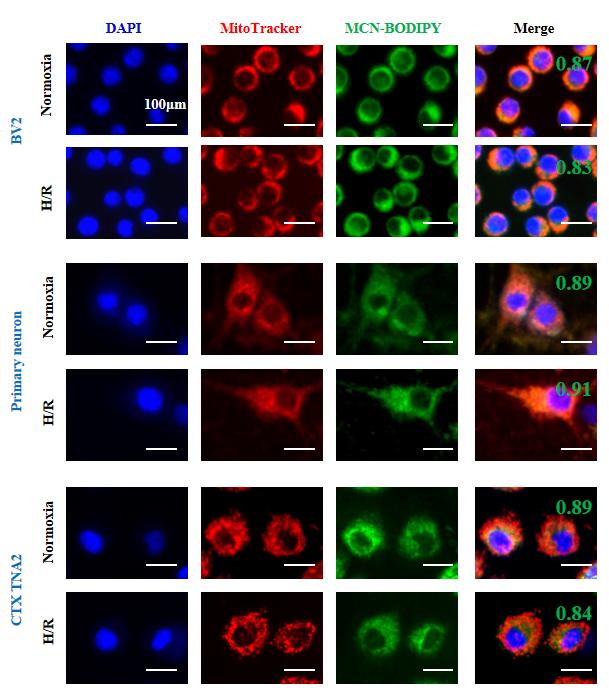


**Figure S15 Representative images of MCN-BODIPY co-localized with mitochondria in different cell types.**

Scale bar: 50 μm. Data were expressed as mean ± SE (in three independent experiments).


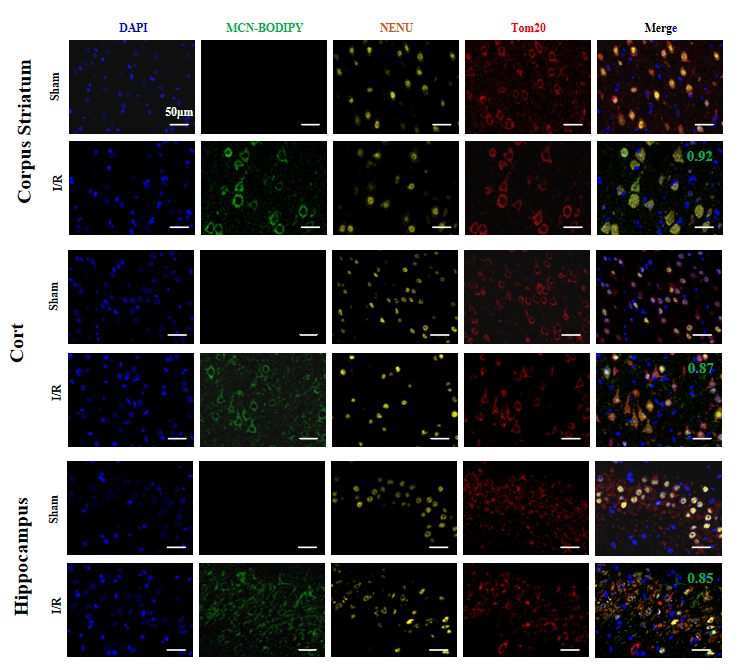


**Figure S16 Representative images of MCN-BODIPY co-localized with mitochondria in different brain regions.** Scale bar: 50 μm. Data were expressed as mean ± SE (in three independent experiments).


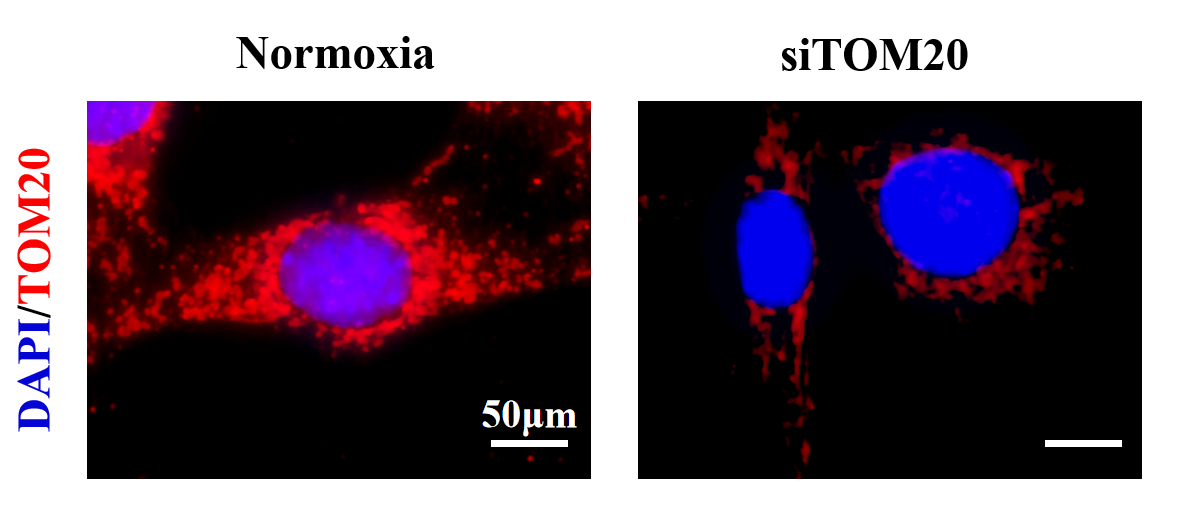

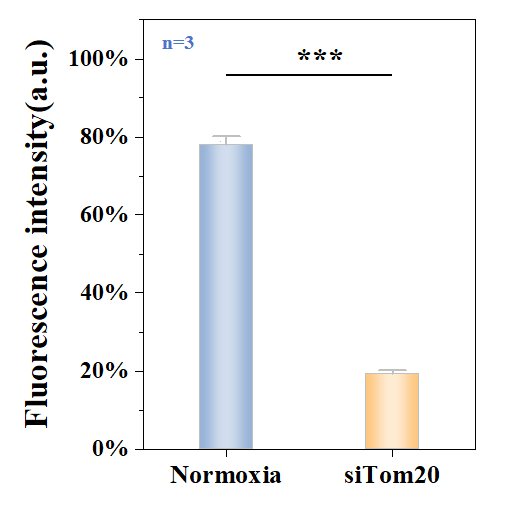


**Figure S17 Representative images (left) and quantitative statistics (right) of TOM20 immunofluorescence staining in different groups.** Data were expressed as mean ± SE (in three independent experiments). Statistical significance was performed by one-way ANOVA with Tukey post hoc test. ns: *P* > 0.05, ^*^*P* < 0.05, ^**^*P* < 0.01, ^***^*P* < 0.001.


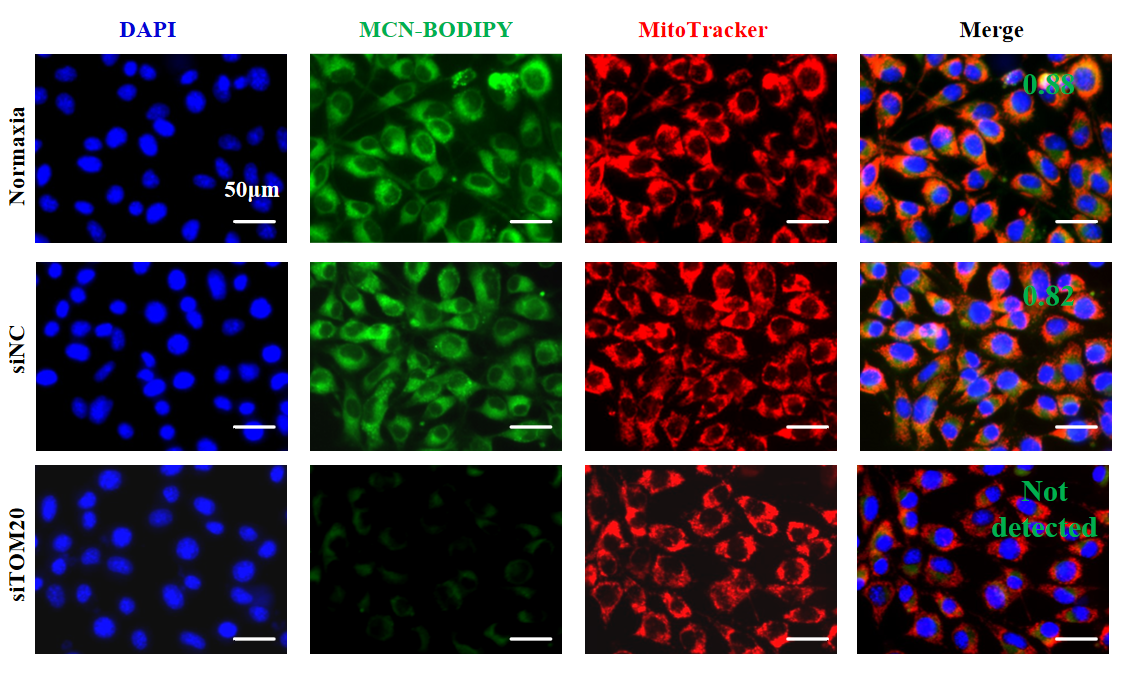


**Figure S18 Representative images of MCN-BODIPY co-localized with mitochondria of HT22 in different groups.** Scale bar: 50 μm. Data were expressed as mean ± SE (in three independent experiments).


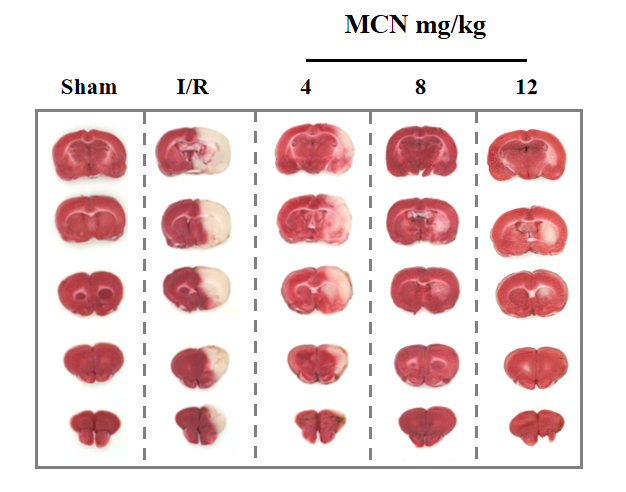


**Figure S19. Dose screening of MCN for the treatment of CIRI. Representative image of TTC staining of rat brain tissue sections after treatment with different doses of MCN.** Data were expressed as mean ± SE (n = 6 animals per group)


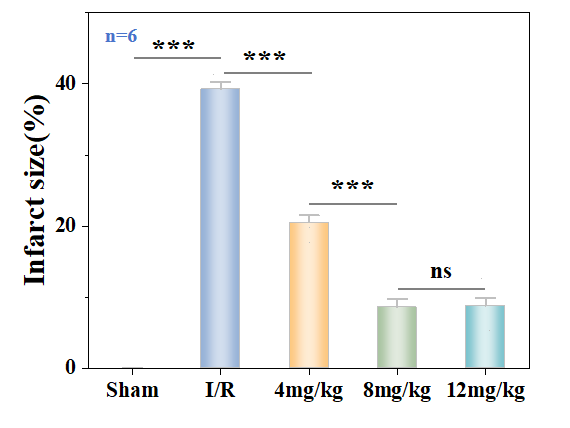


**Figure S20 Dose screening of MCN for the treatment of CIRI. Statistical analysis of TTC staining of rat brain tissue sections after treatment with different doses of MCN.** Data were expressed as mean ± SE (n =6 animals per group). Statistical significance was performed by one-way ANOVA with Tukey post hoc test. ns: *P* > 0.05, ^*^*P* < 0.05, ^**^*P* < 0.01, ^***^*P* < 0.001.


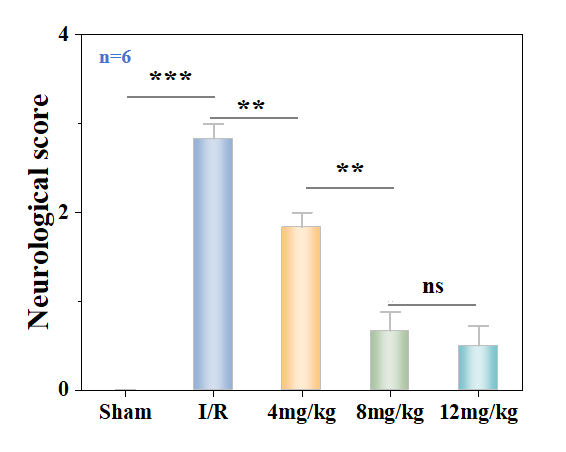


**Figure S21 Dose screening of MCN for the treatment of CIRI. Neurological function scores of rats after treatment with different doses of MCN.** Data were expressed as mean ± SE (n = 6 animals per group). Statistical significance was performed by one-way ANOVA with Tukey post hoc test. ns: *P* > 0.05, ^*^*P* < 0.05, ^**^*P* < 0.01, ^***^*P* < 0.001.


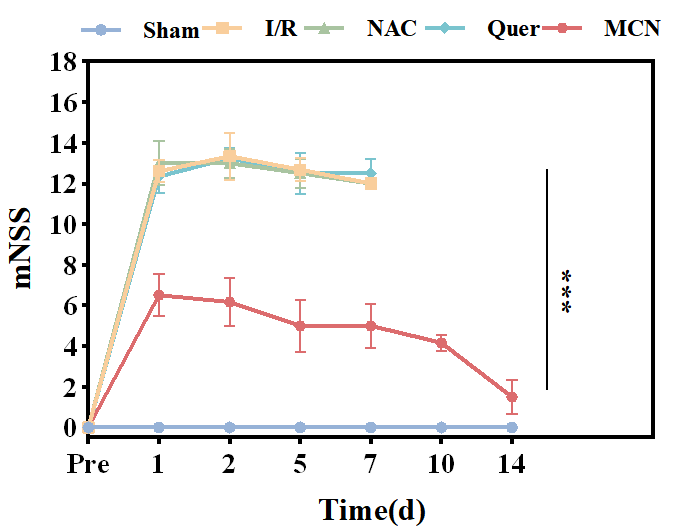


**Figure S22 Mean mNSS scores of rats in different treatment groups during the treatment period.** Data were expressed as mean ± SE (n = 6 animals per group). Statistical significance was performed by one-way ANOVA with Tukey post hoc test. ns: *P* > 0.05, ^*^*P* < 0.05, ^**^*P* < 0.01, ^***^*P* < 0.001. It should be noted that no rats in the I/R, NAC, or Quer groups survived beyond day 10.


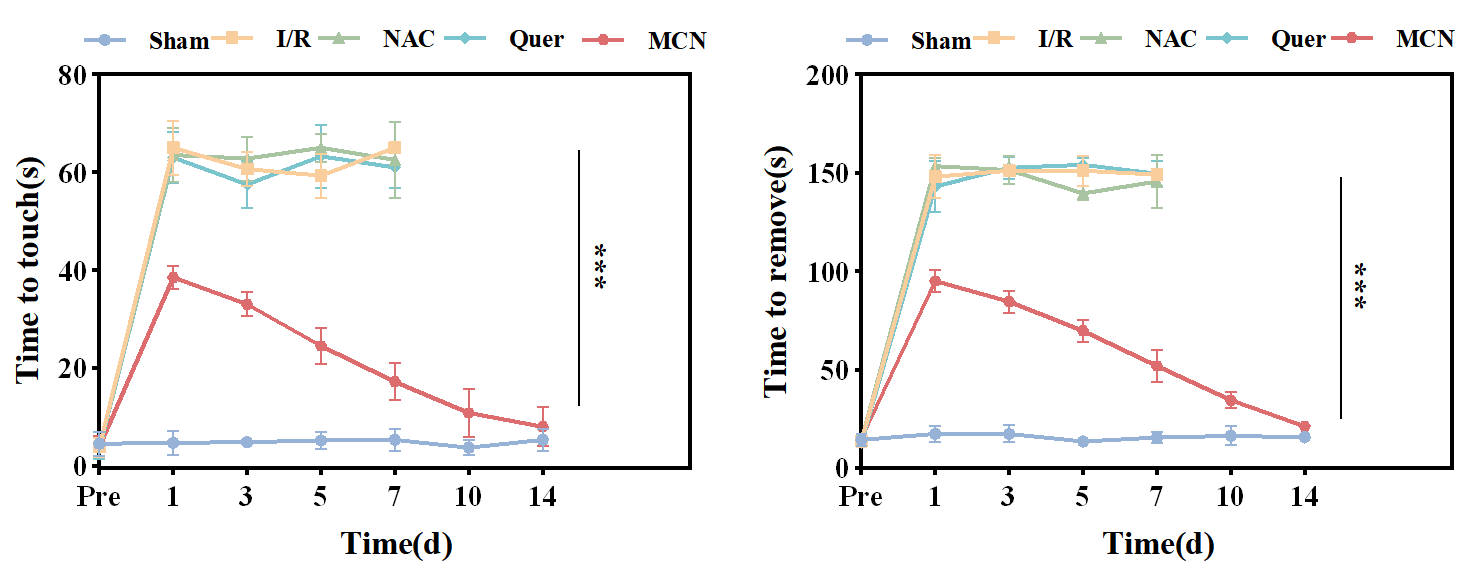


**Figure S23 Adhesive tests evaluate rat behavior, including the first-time rats spend in contact with the tape.** Data were expressed as mean ± SE (n = 6 animals per group). Statistical significance was performed by one-way ANOVA with Tukey post hoc test. ns: *P* > 0.05, ^*^*P* < 0.05, ^**^*P* < 0.01, ^***^*P* < 0.001. It should be noted that no rats in the I/R, NAC, or Quer groups survived beyond day 10.


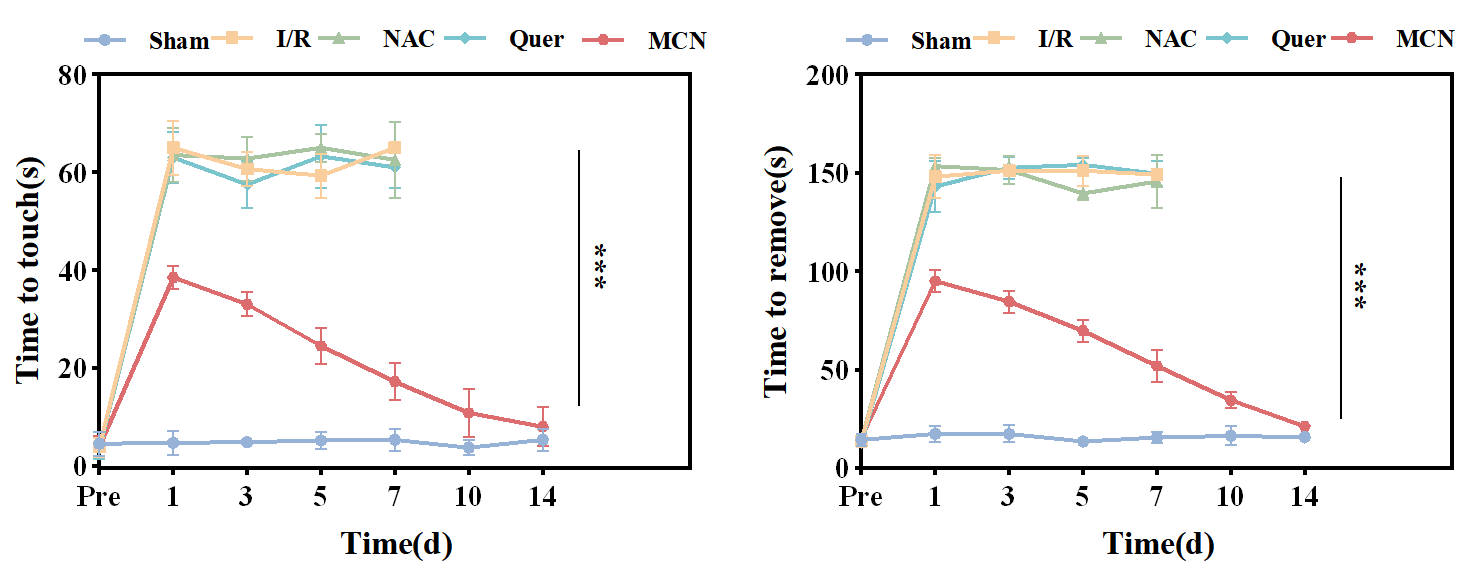


**Figure S24 Adhesive tests evaluate rat behavior, including the time it takes to successfully remove the tape (right).** Data were expressed as mean ± SE (n = 6 animals per group). Statistical significance was performed by one-way ANOVA with Tukey post hoc test. ns: *P* > 0.05, ^*^*P* < 0.05, ^**^*P* < 0.01, ^***^*P* < 0.001. It should be noted that no rats in the I/R, NAC, or Quer groups survived beyond day 10.


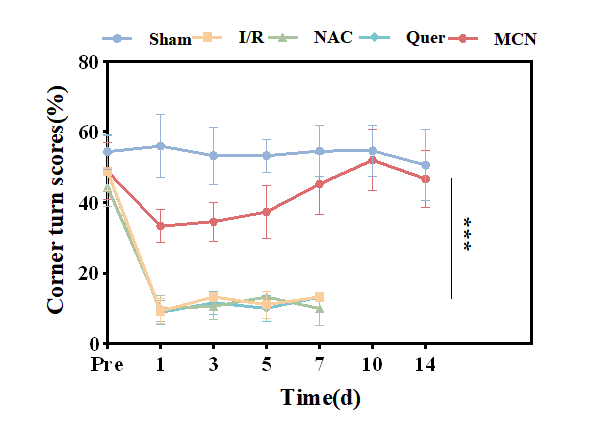


**Figure S25 Corner turn test result.** Data were expressed as mean ± SE (n = 6 animals per group). Statistical significance was performed by one-way ANOVA with Tukey post hoc test. ns: *P* > 0.05, ^*^*P* < 0.05, ^**^*P* < 0.01, ^***^*P* < 0.001. It should be noted that no rats in the I/R, NAC, or Quer groups survived beyond day 10.


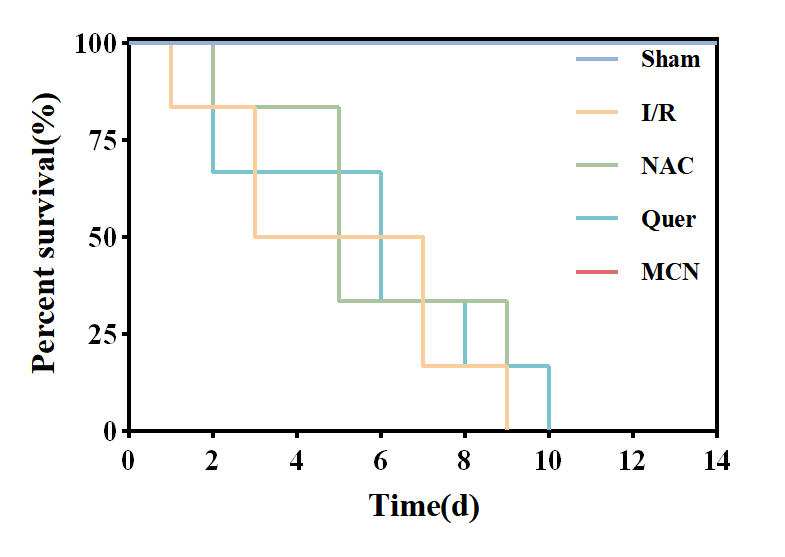


**Figure S26 Survival rate.** Data were expressed as mean ± SE (n = 6 animals per group). It should be noted that no rats in the I/R, NAC, or Quer groups survived beyond day 10.


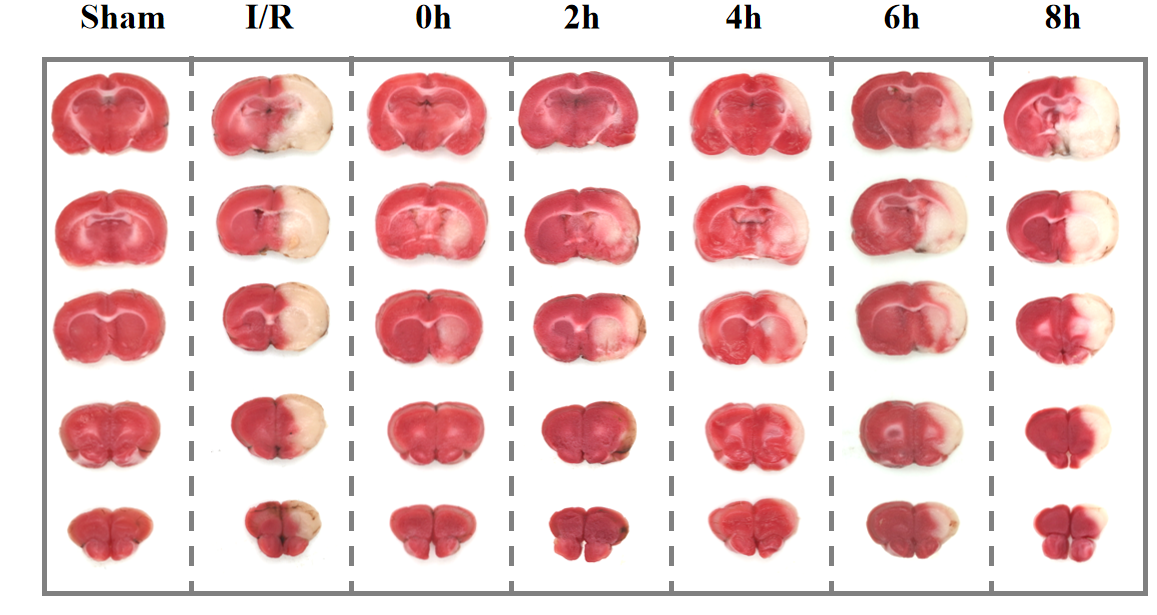


**Figure S27 Representative image of TTC staining of brain tissue in different delayed time points after reperfusion in rats.** Data were expressed as mean ± SE (n = 6 animals per group).


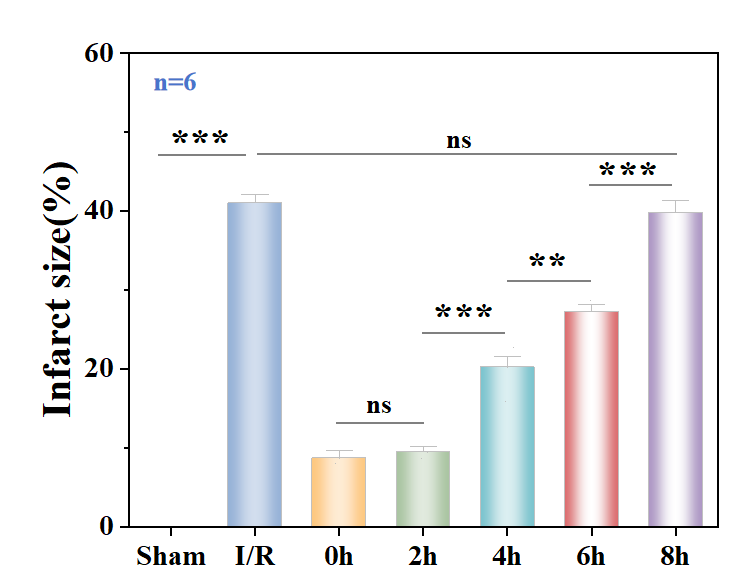


**Figure S28 Infarct area statistics of brain tissue in different delayed time points after reperfusion in rats.** Data were expressed as mean ± SE (n = 6 animals per group). Statistical significance was performed by one-way ANOVA with Tukey post hoc test. ns: *P* > 0.05, ^*^*P* < 0.05, ^**^*P* < 0.01, ^***^*P* < 0.001.


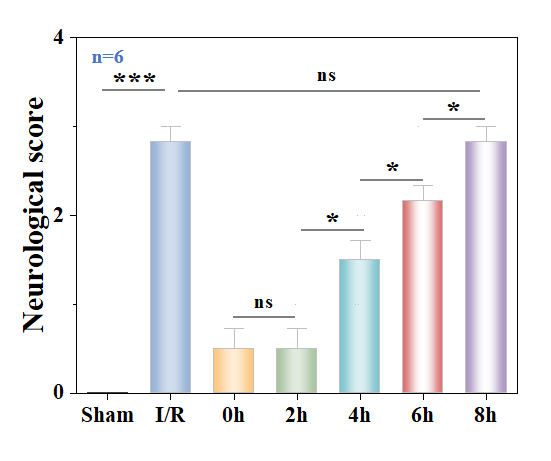


**Figure S29 Neurological function scores in different delayed time points after reperfusion in rats.** Data were expressed as mean ± SE (n = 6 animals per group). Statistical significance was performed by one-way ANOVA with Tukey post hoc test. ns: *P* > 0.05, ^*^*P* < 0.05, ^**^*P* < 0.01, ^***^*P* < 0.001.


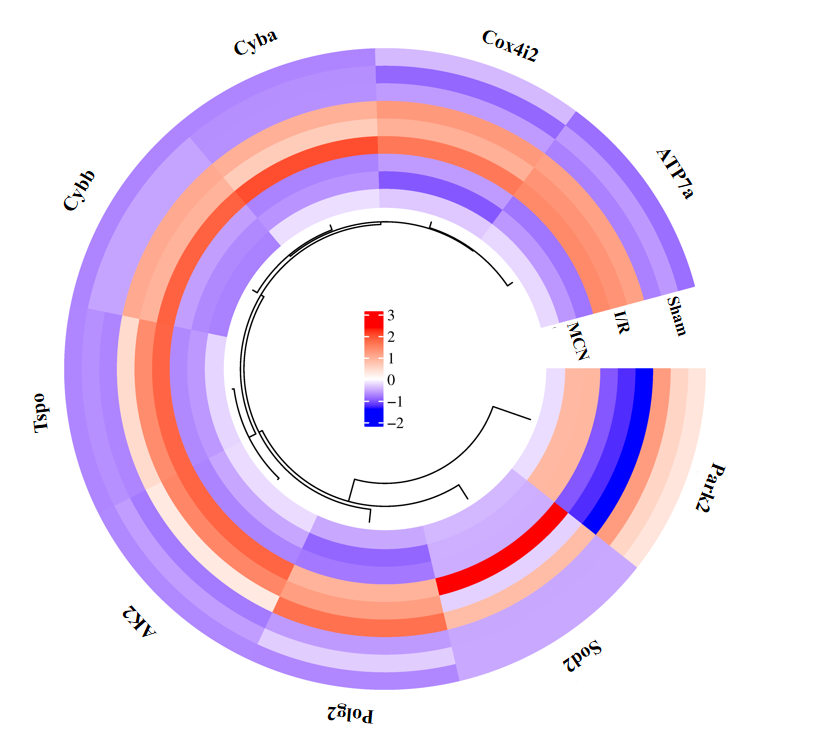


**Figure S30 Heatmap of selected mitochondria-related genes in the Sham, I/R, and MCN groups.**


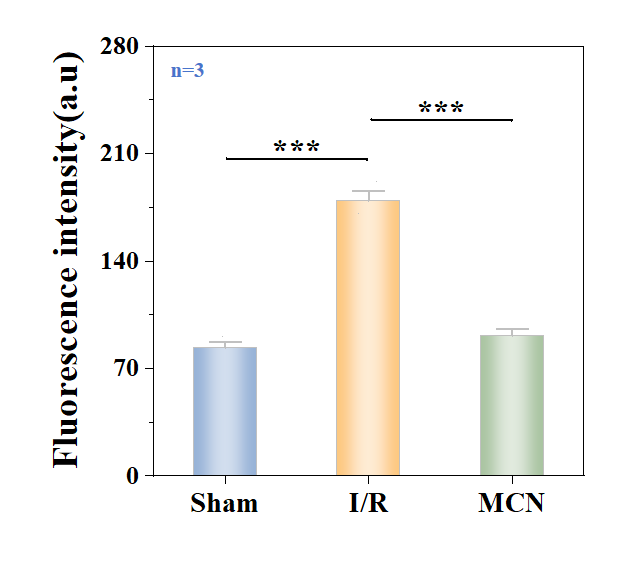


**Figure S31 Quantitative statistics of ROS immunofluorescence staining in brain tissue.** Data were expressed as mean ± SE (in three independent experiments). Statistical significance was performed by one-way ANOVA with Tukey post hoc test. ns: *P* > 0.05, ^*^*P* < 0.05, ^**^*P* < 0.01, ^***^*P* < 0.001.


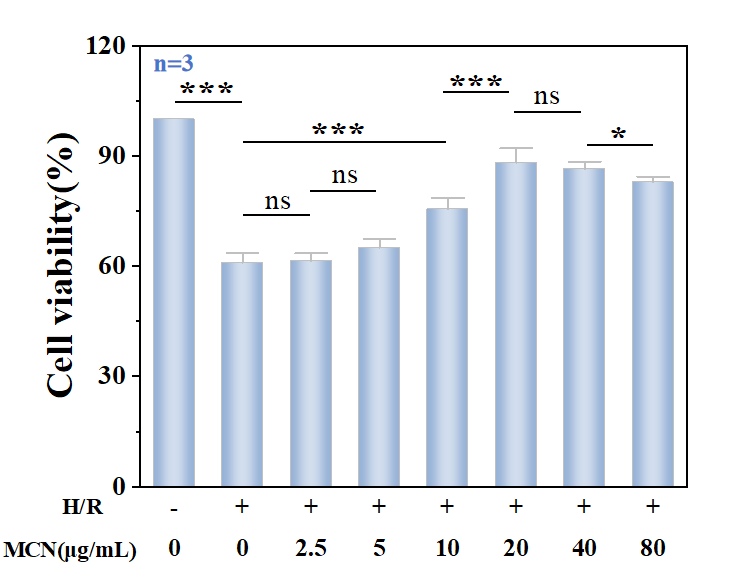


**Figure S32 Effects of MCN on the viability of HT22 cells induced by H/R.** Data were expressed as mean ± SE (in three independent experiments). Statistical significance was performed by one-way ANOVA with Tukey post hoc test. ns: *P* > 0.05, ^*^*P* < 0.05, ^**^*P* < 0.01, ^***^*P* < 0.001.


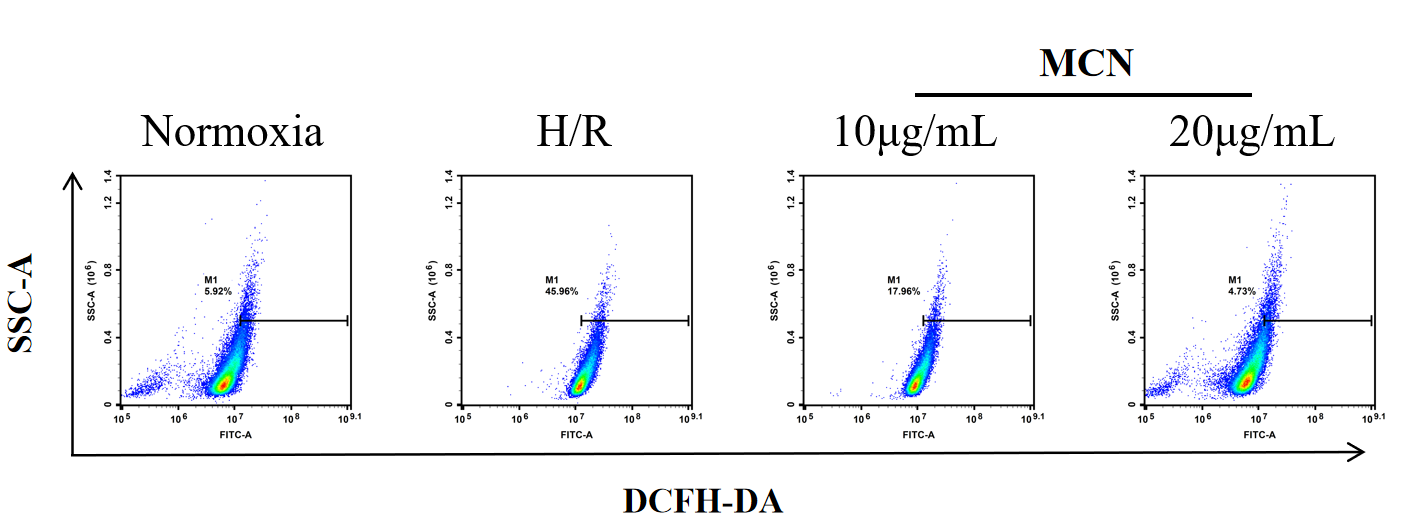


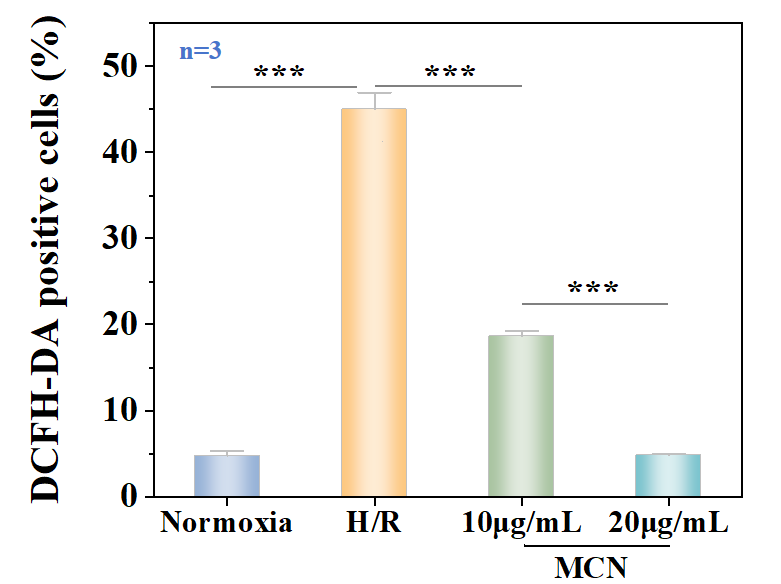


**Figure S33 Flow cytometric analysis of ROS (above) in HT22 cells and quantification analysis(below) of the relative mean fluorescence intensity.** Data were expressed as mean ± SE (in three independent experiments). Statistical significance was performed by one-way ANOVA with Tukey post hoc test. ns: *P* > 0.05, ^*^*P* < 0.05, ^**^*P* < 0.01, ^***^*P* < 0.001.


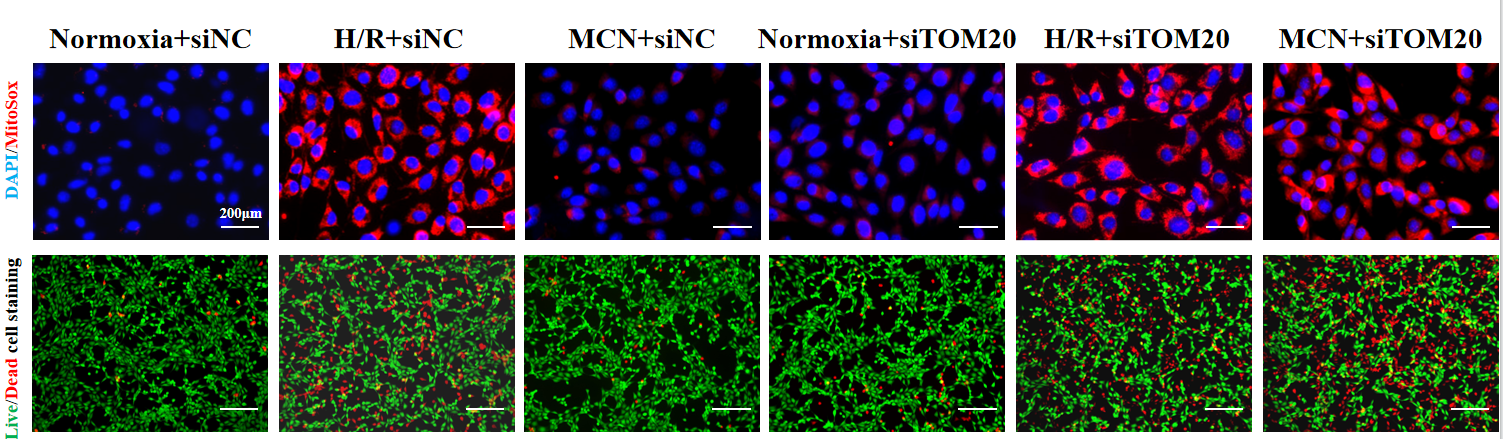


**Figure S34 Representative images of MitoSOX staining(above) and Live/Dead cells staining (below) in HT22 cells in different treatment groups.** Data were expressed as mean ± SE (in three independent experiments).


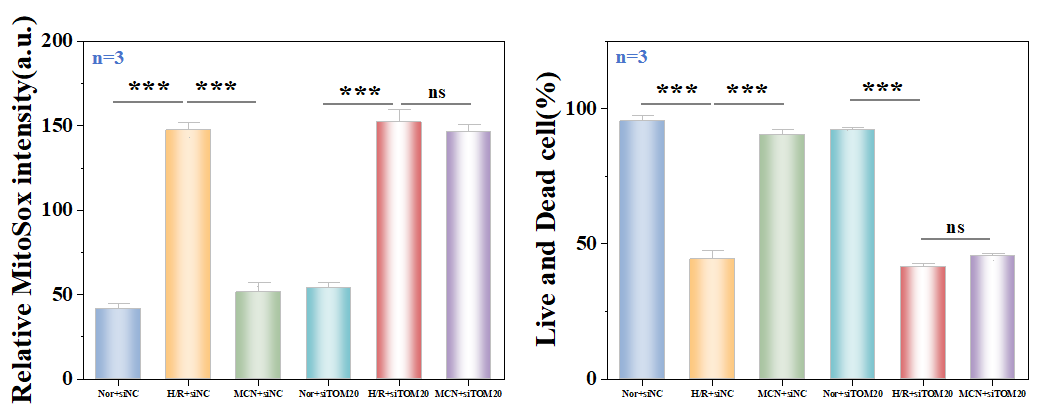


**Figure S35 Statistical analysis of MitoSOX staining(above) and Live/Dead cells staining (below) in HT22 cells in different treatment groups.** Data were expressed as mean ± SE (in three independent experiments). Statistical significance was performed by one-way ANOVA with Tukey post hoc test. ns: *P* > 0.05, ^*^*P* < 0.05, ^**^*P* < 0.01, ^***^*P* < 0.001.


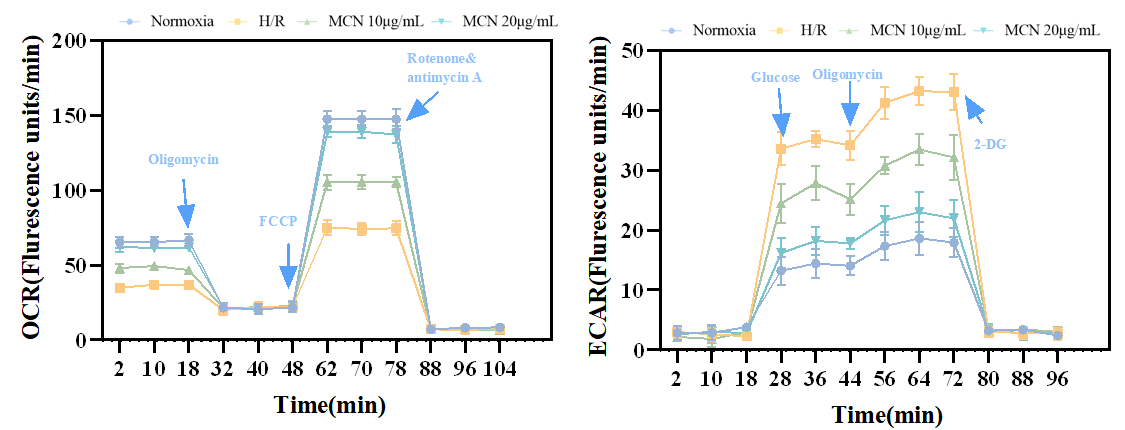


**Figure S36 Effects of MCN on OCR and ECAR in HT22.** Data were expressed as mean ± SE (in three independent experiments).


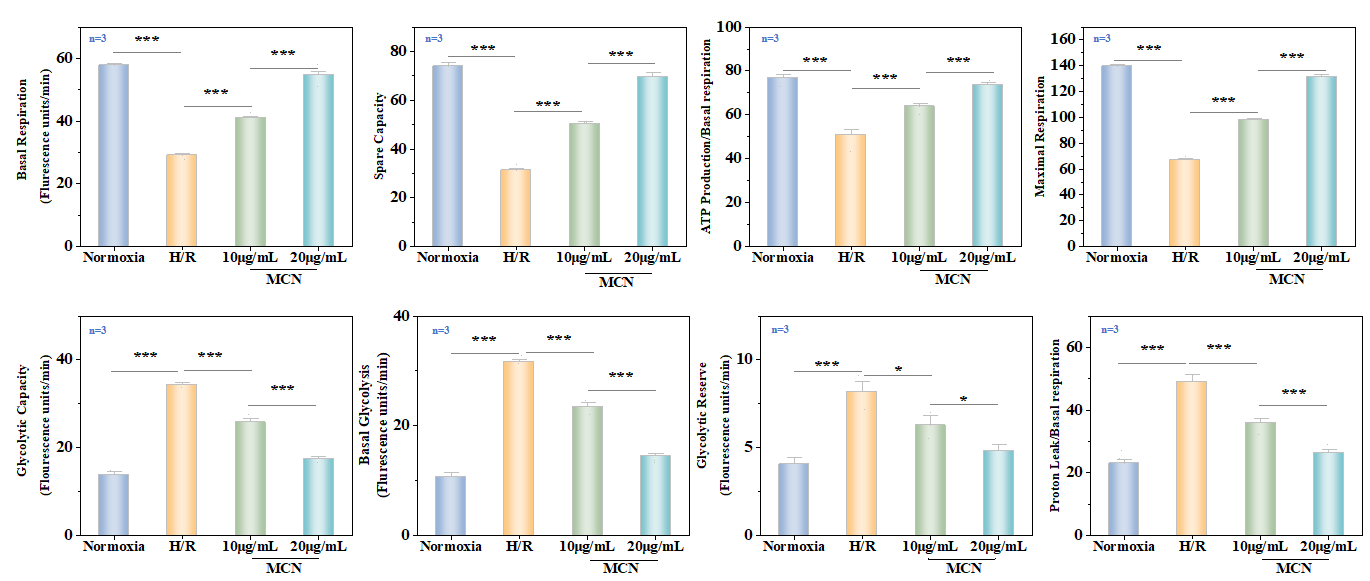


**Figure S37 Statistical analysis of MCN on OCR and ECAR in HT22.** Data were expressed as mean ± SE (in three independent experiments). Statistical significance was performed by one-way ANOVA with Tukey post hoc test. ns: *P* > 0.05, ^*^*P* < 0.05, ^**^*P* < 0.01, ^***^*P* < 0.001.


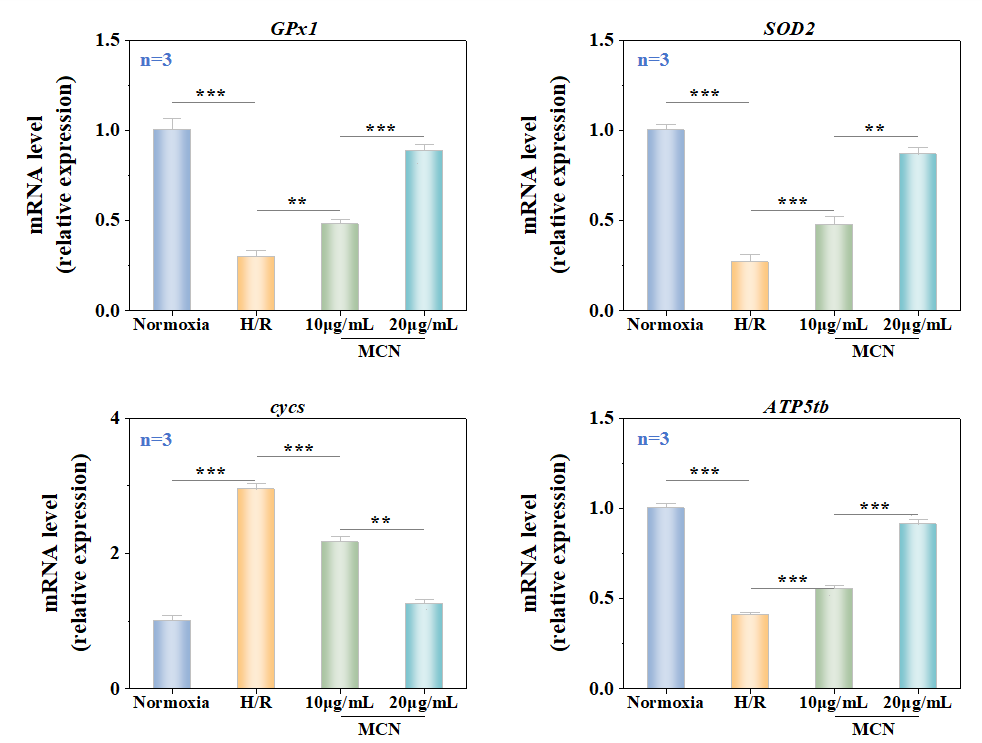


**Figure S38 QPCR was used to detect the expression levels of *GPx1*, *SOD2*, *cycs* and *ATP5tb* in HT22 cell of different treatment groups.** Data were expressed as mean ± SE (in three independent experiments). Statistical significance was performed by one-way ANOVA with Tukey post hoc test. ns: *P* > 0.05, ^*^*P* < 0.05, ^**^*P* < 0.01, ^***^*P* < 0.001.


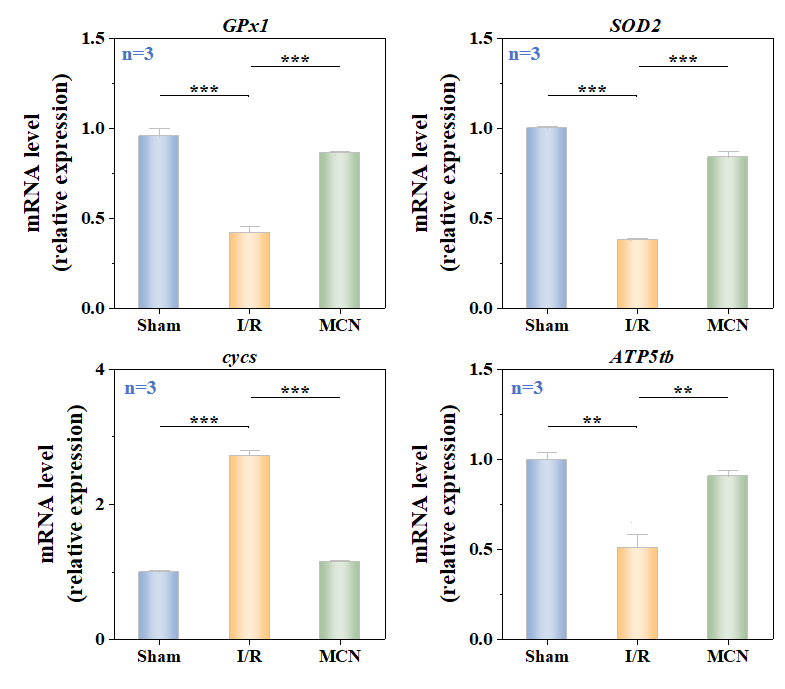


**Figure S39 QPCR was used to detect the expression levels of *GPx1*, *SOD2*, *cycs* and *ATP5tb* in brain tissue of different treatment groups.** Data were expressed as mean ± SE (in three independent experiments). Statistical significance was performed by one-way ANOVA with Tukey post hoc test. ns: *P* > 0.05, ^*^*P* < 0.05, ^**^*P* < 0.01, ^***^*P* < 0.001.


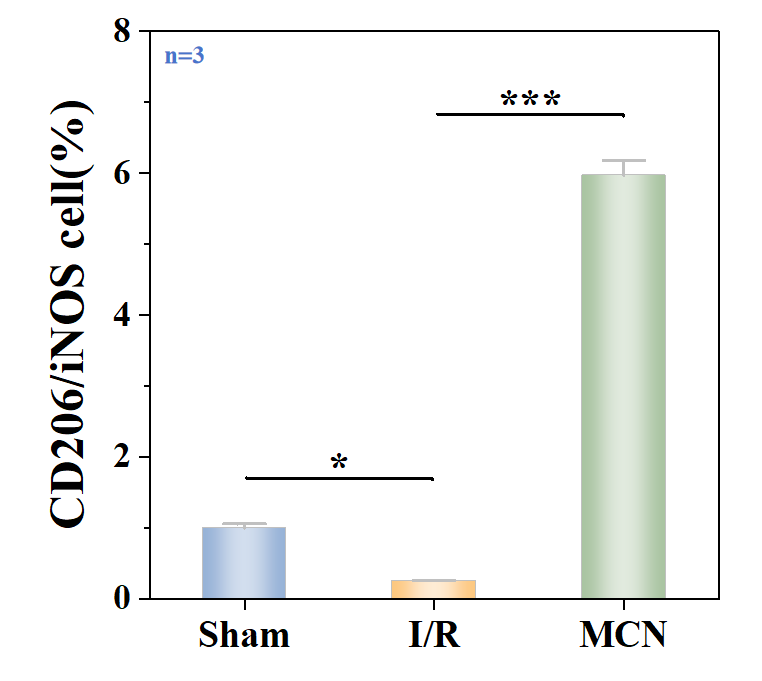


**Figure S40 Quantitative statistics of CD206/iNOS fluorescence in the cerebral infarction area of each group.** Data were expressed as mean ± SE (in three independent experiments). Statistical significance was performed by one-way ANOVA with Tukey post hoc test. ns: *P* > 0.05, ^*^*P* < 0.05, ^**^*P* < 0.01, ^***^*P* < 0.001.


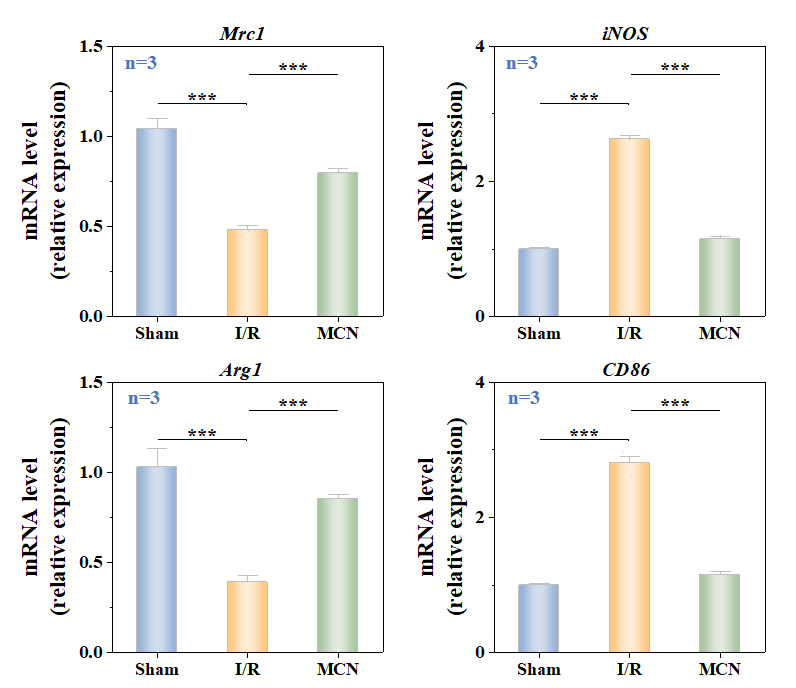


**Figure S41 QPCR was used to detect the expression levels of *Mrc1*, *Arg1*, *CD86* and *iNOS* in brain tissue of different treatment groups.** Data were expressed as mean ± SE (in three independent experiments). Statistical significance was performed by one-way ANOVA with Tukey post hoc test. ns: *P* > 0.05, ^*^*P* < 0.05, ^**^*P* < 0.01, ^***^*P* < 0.001.


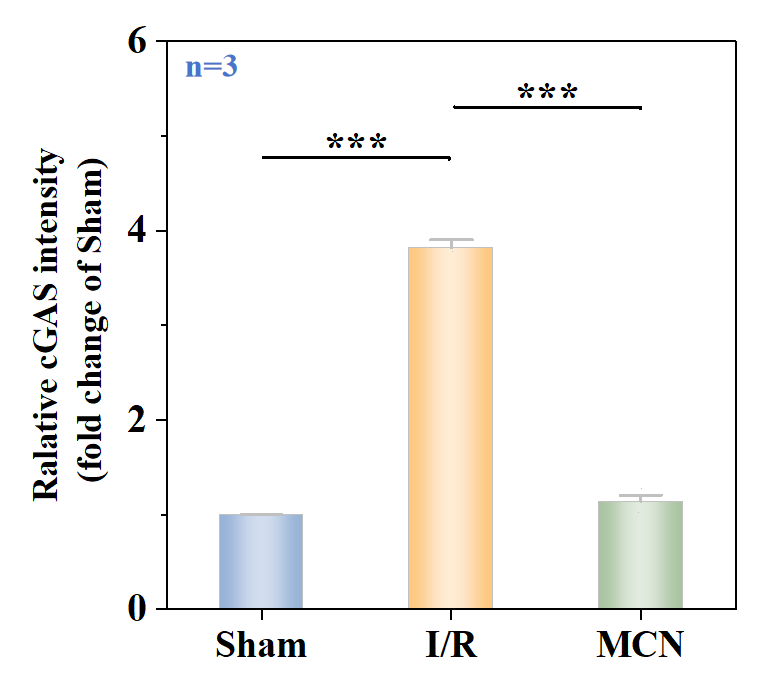


**Figure S42 Statistical analysis of cGAS immunohistochemical staining in rat brain tissue from different treatment groups. Scale bar: 20 μm.** Data were expressed as mean ± SE (in three independent experiments). Statistical significance was performed by one-way ANOVA with Tukey post hoc test. ns: *P* > 0.05, ^*^*P* < 0.05, ^**^*P* < 0.01, ^***^*P* < 0.001.


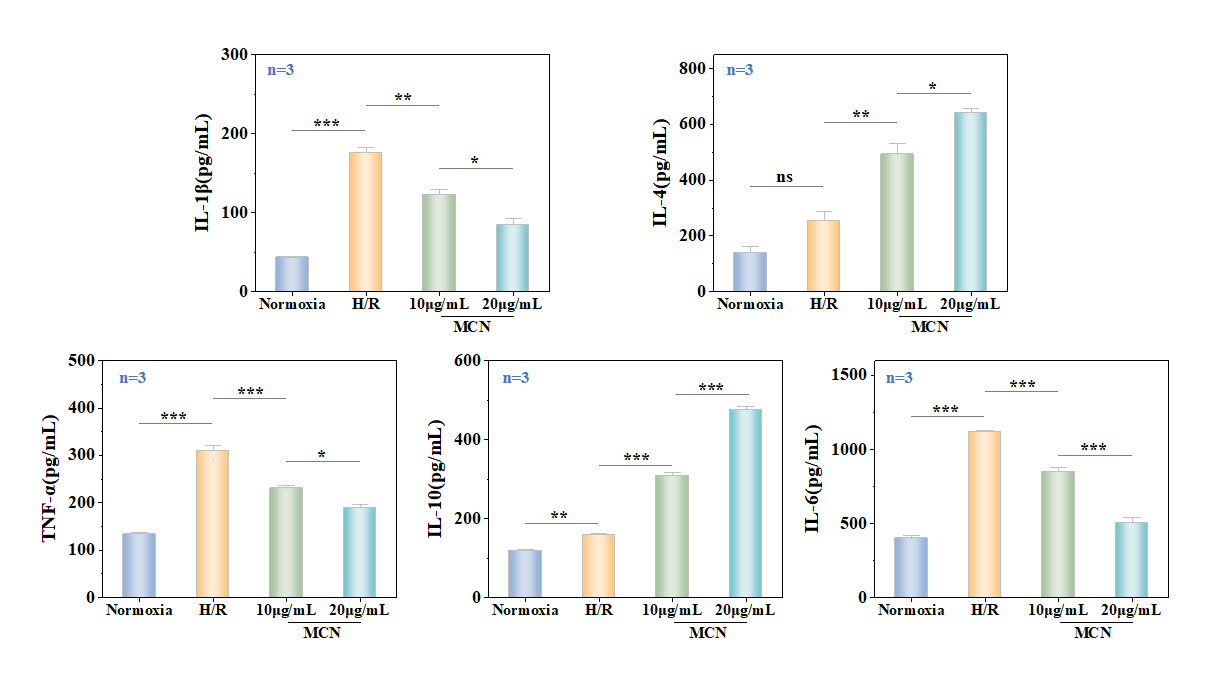


**Figure S43 Etection of inflammatory factor levels in BV2 cells in different treatment groups. TNF-α、IL-1β、 IL-6、IL-4、IL-10.** Data were expressed as mean ± SE (in three independent experiments). Statistical significance was performed by one-way ANOVA with Tukey post hoc test. ns: *P* > 0.05, ^*^*P* < 0.05, ^**^*P* < 0.01, ^***^*P* < 0.001


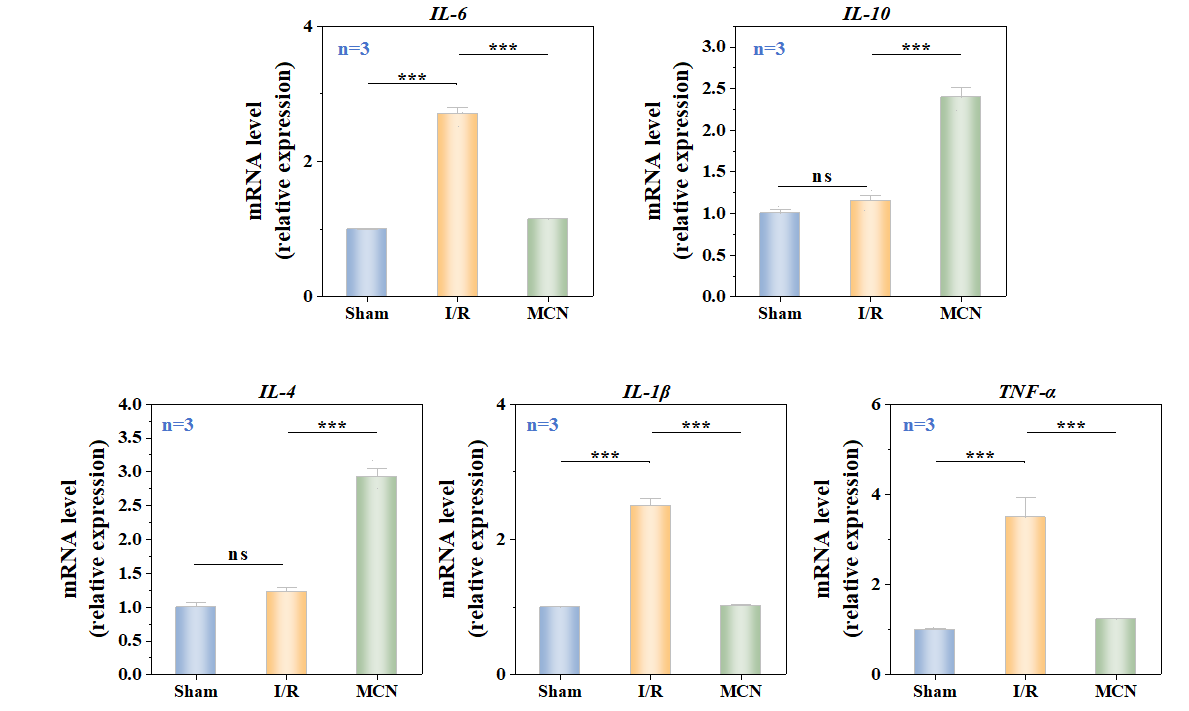


**Figure S44 QPCR was used to detect the expression levels of *IL-6*, *IL-10*, *IL-4*, *IL-1β* and *TNF-α* in brain tissue of different treatment groups.** Data were expressed as mean ± SE (in three independent experiments). Statistical significance was performed by one-way ANOVA with Tukey post hoc test. ns: *P* > 0.05, ^*^*P* < 0.05, ^**^*P* < 0.01, ^***^*P* < 0.001.


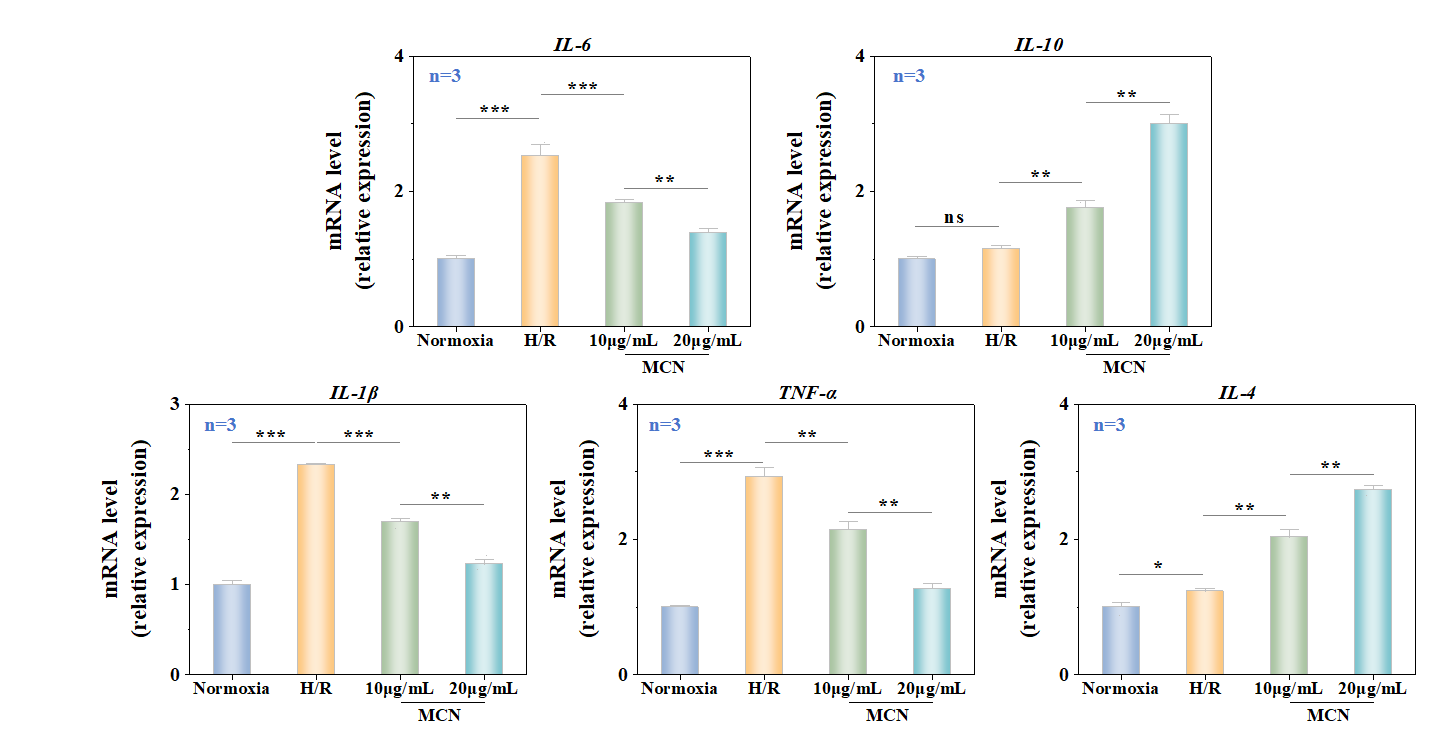


**Figure S45 QPCR was used to detect the expression levels of *IL-6*, *IL-10*, *IL-4*, *IL-1β* and *TNF-α* in BV2 cell of different treatment groups.** Data were expressed as mean ± SE (in three independent experiments). Statistical significance was performed by one-way ANOVA with Tukey post hoc test. ns: *P* > 0.05, ^*^*P* < 0.05, ^**^*P* < 0.01, ^***^*P* < 0.001.


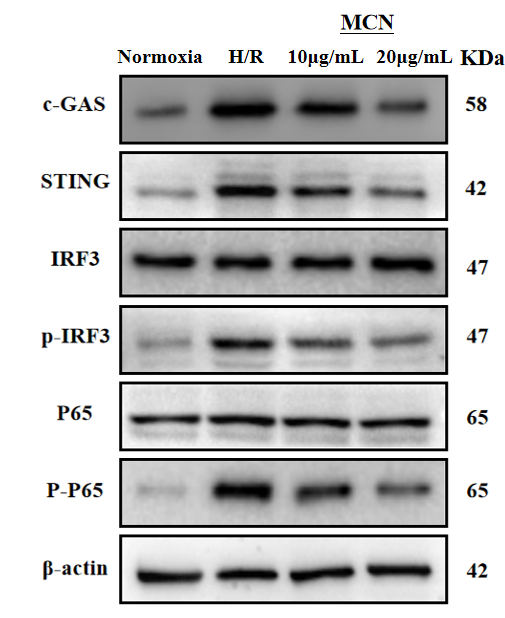


**Figure S46 Representative diagram of WB detection of apoptosis-related protein expression in BV2 cells.**Data were expressed as mean ± SE (in three independent experiments).


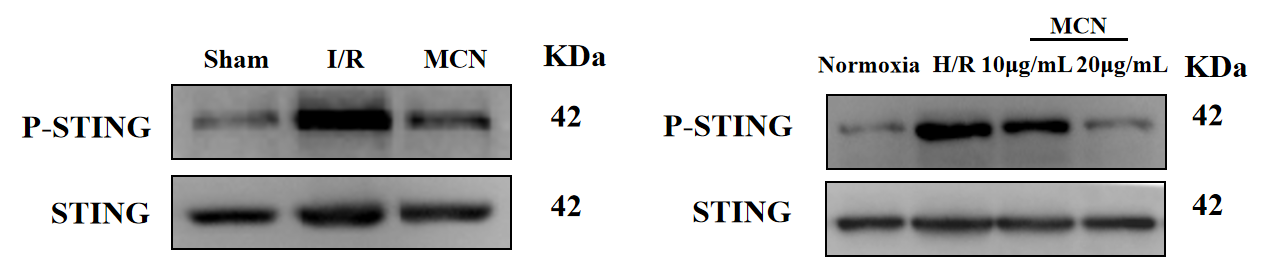


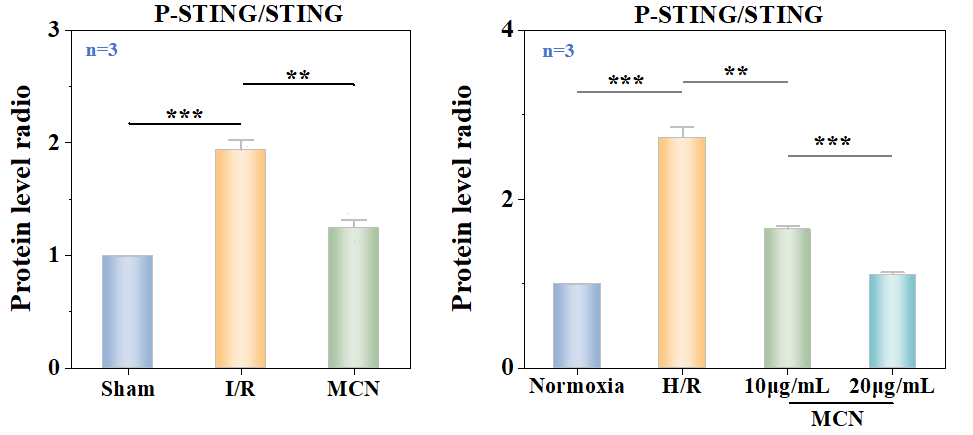


**Figure S47 Representative diagram(above) and statistical analysis (below) of WB detection of P-STING protein expression in BV2 cells and brain tisue.**Data were expressed as mean ± SE (in three independent experiments). Statistical significance was performed by one-way ANOVA with Tukey post hoc test. ns: *P* > 0.05, ^*^*P* < 0.05, ^**^*P* < 0.01, ^***^*P* < 0.001.


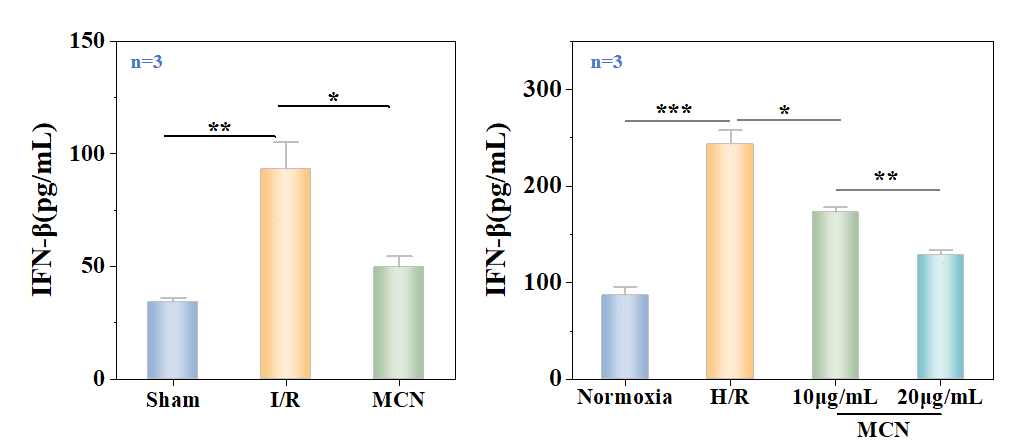


**Figure S48 Expression of IFN-β in brain tissue and BV2 of different treatment groups.** Data were expressed as mean ± SE (in three independent experiments). Statistical significance was performed by one-way ANOVA with Tukey post hoc test. ns: *P* > 0.05, ^*^*P* < 0.05, ^**^*P* < 0.01, ^***^*P* < 0.001.


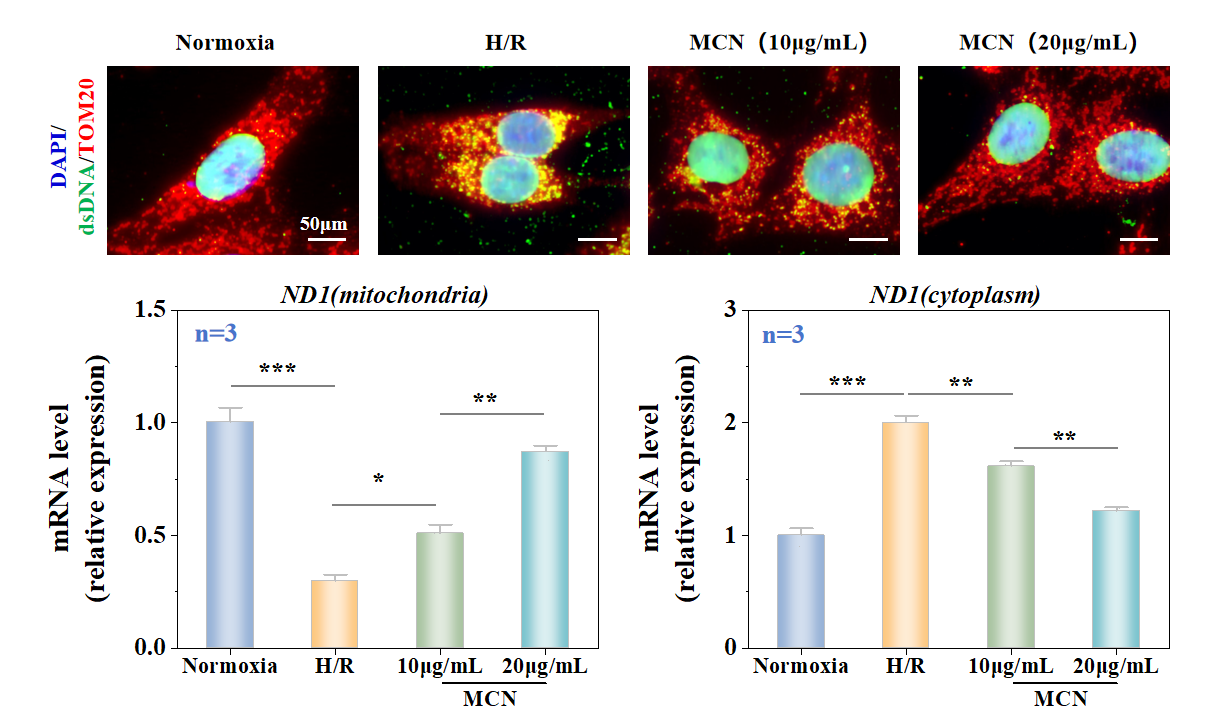


**Figure S49 Representative images of dsDNA/Tom20 immunofluorescence staining (above) and qPCR was used to detect the expression levels of mtDNA (below) of HT22 cells from different treatment groups. Scale bar: 50 μm.** Data were expressed as mean ± SE (in three independent experiments). Statistical significance was performed by one-way ANOVA with Tukey post hoc test. ns: *P* > 0.05, ^*^*P* < 0.05, ^**^*P* < 0.01, ^***^*P* < 0.001.


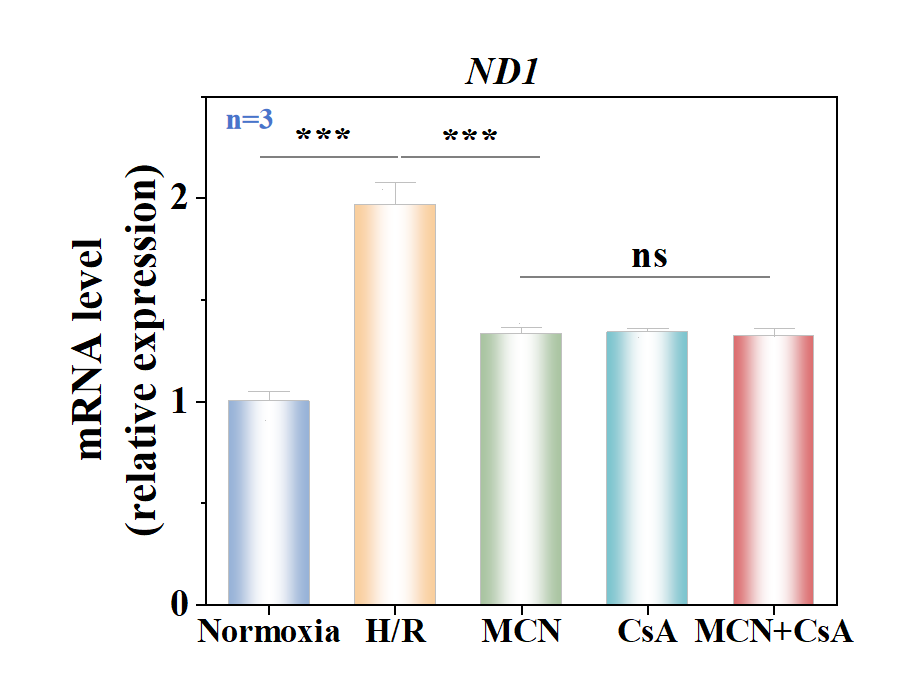


**Figure S50 QPCR was used to detect the expression levels of mtDNA of HT22 cells from different treatment groups.** Data were expressed as mean ± SE (in three independent experiments). Statistical significance was performed by one-way ANOVA with Tukey post hoc test. ns: *P* > 0.05, ^*^*P* < 0.05, ^**^*P* < 0.01, ^***^*P* < 0.001.


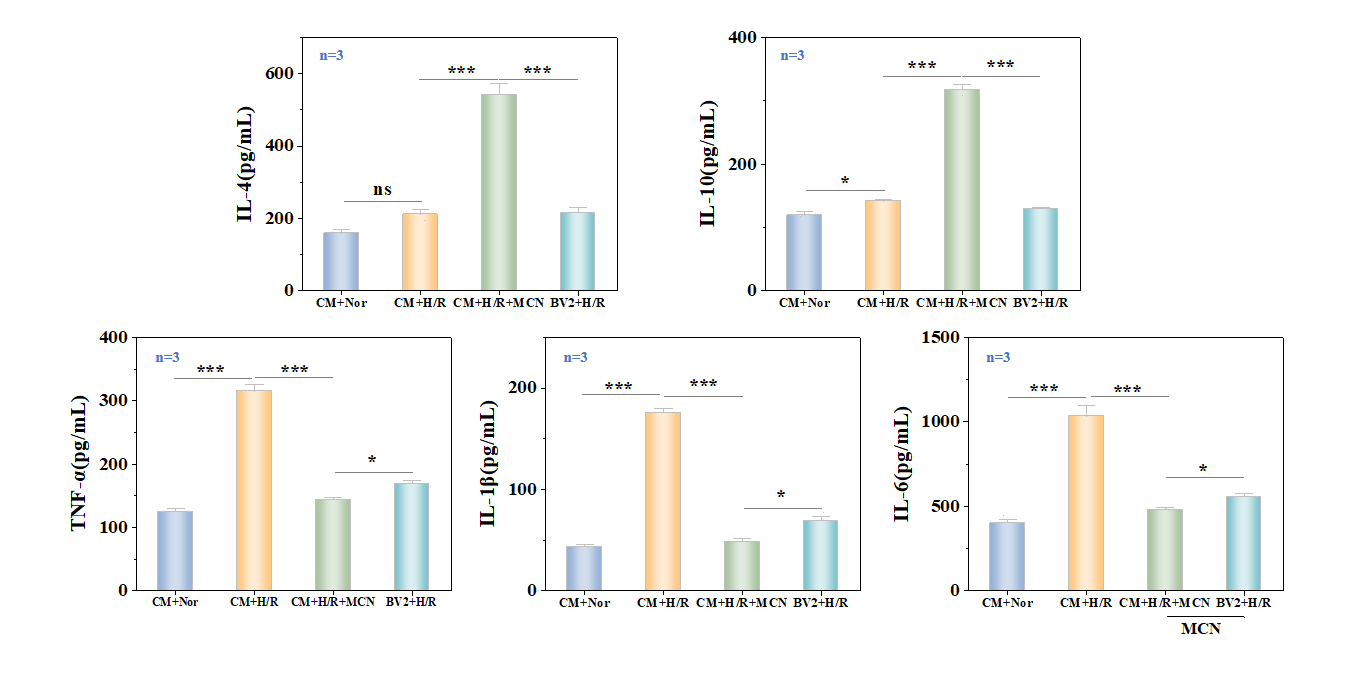


**Figure S51 Expression of pro-inflammatory and anti-inflammatory factors (IL-10, IL-6, TNF-α, IL-1β, IL-4)) in BV2 of different treatment groups.** Data were expressed as mean ± SE (in three independent experiments). Statistical significance was performed by one-way ANOVA with Tukey post hoc test. ns: *P* > 0.05, ^*^*P* < 0.05, ^**^*P* < 0.01, ^***^*P* < 0.001.


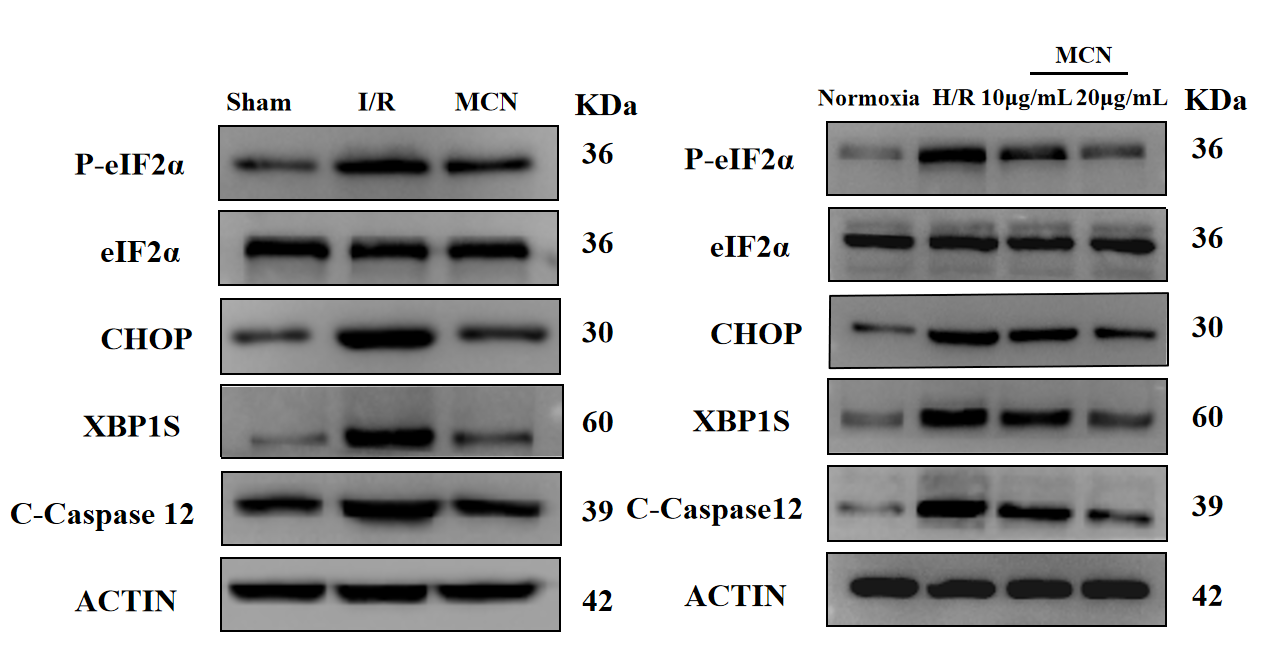


**Figure S52 Representative diagram of WB detection of ER stress-related protein expression in HT22 cells and brain tissue.** Data were expressed as mean ± SE (in three independent experiments).


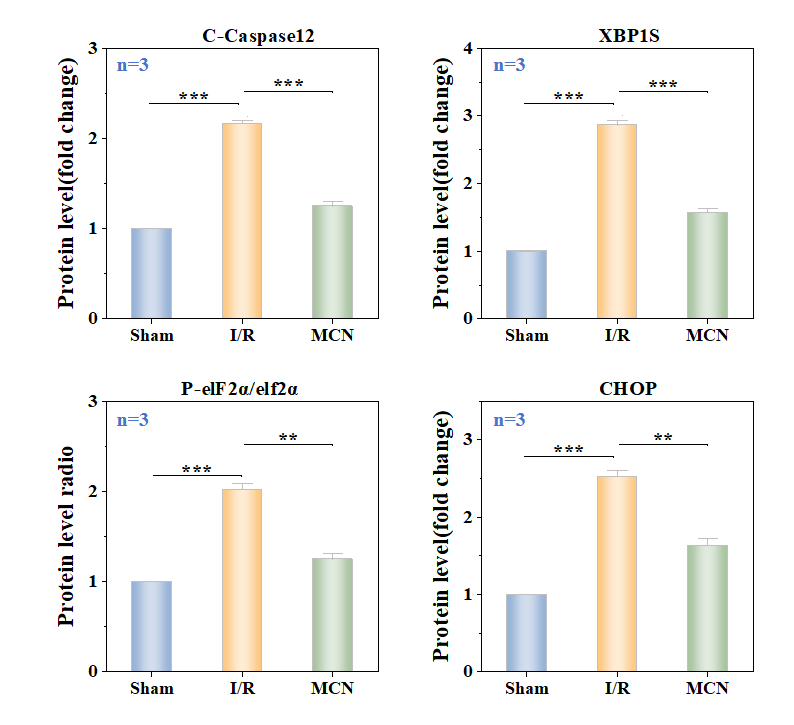


**Figure S53 Statistical analysis of WB detection of ER stress-related protein expression in brain tissue.** Data were expressed as mean ± SE (in three independent experiments). Statistical significance was performed by one-way ANOVA with Tukey post hoc test. ns: *P* > 0.05, ^*^*P* < 0.05, ^**^*P* < 0.01, ^***^*P* < 0.001.


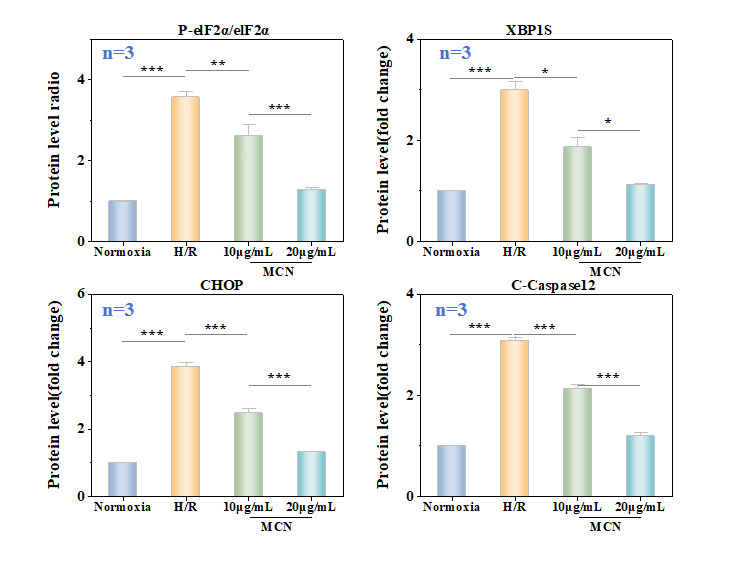


**Figure S54 Statistical analysis of WB detection of ER stress-related protein expression in HT22 cells.** Data were expressed as mean ± SE (in three independent experiments). Statistical significance was performed by one-way ANOVA with Tukey post hoc test. ns: *P* > 0.05, ^*^*P* < 0.05, ^**^*P* < 0.01, ^***^*P* < 0.001.


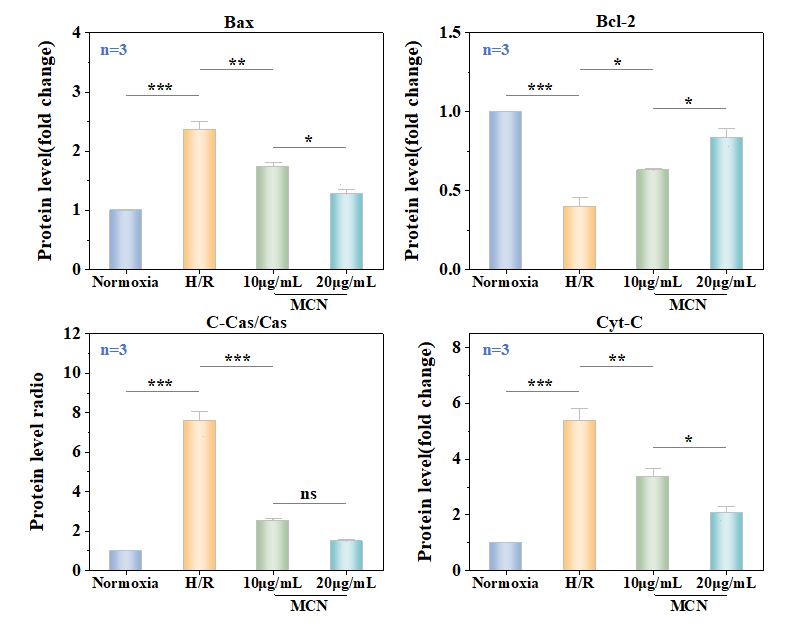


**Figure S55 Statistical analysis of WB detection of apoptosis-related protein expression in HT22 cells.** Data were expressed as mean ± SE (in three independent experiments). Statistical significance was performed by one-way ANOVA with Tukey post hoc test. ns: *P* > 0.05, ^*^*P* < 0.05, ^**^*P* < 0.01, ^***^*P* < 0.001.


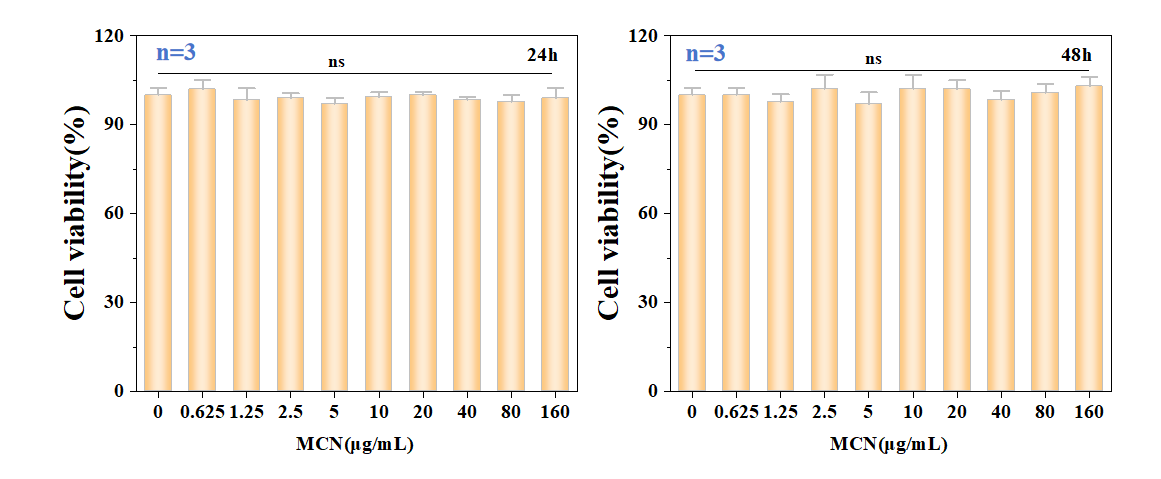


**Figure S56 CCK8 assay to detect the effect of different concentrations of MCN on cell viability after 24 h and 48 h treatment of HT22 cells.** Data were expressed as mean ± SE (in three independent experiments). Statistical significance was performed by one-way ANOVA with Tukey post hoc test. ns: *P* > 0.05, ^*^*P* < 0.05, ^**^*P* < 0.01, ^***^*P* < 0.001.


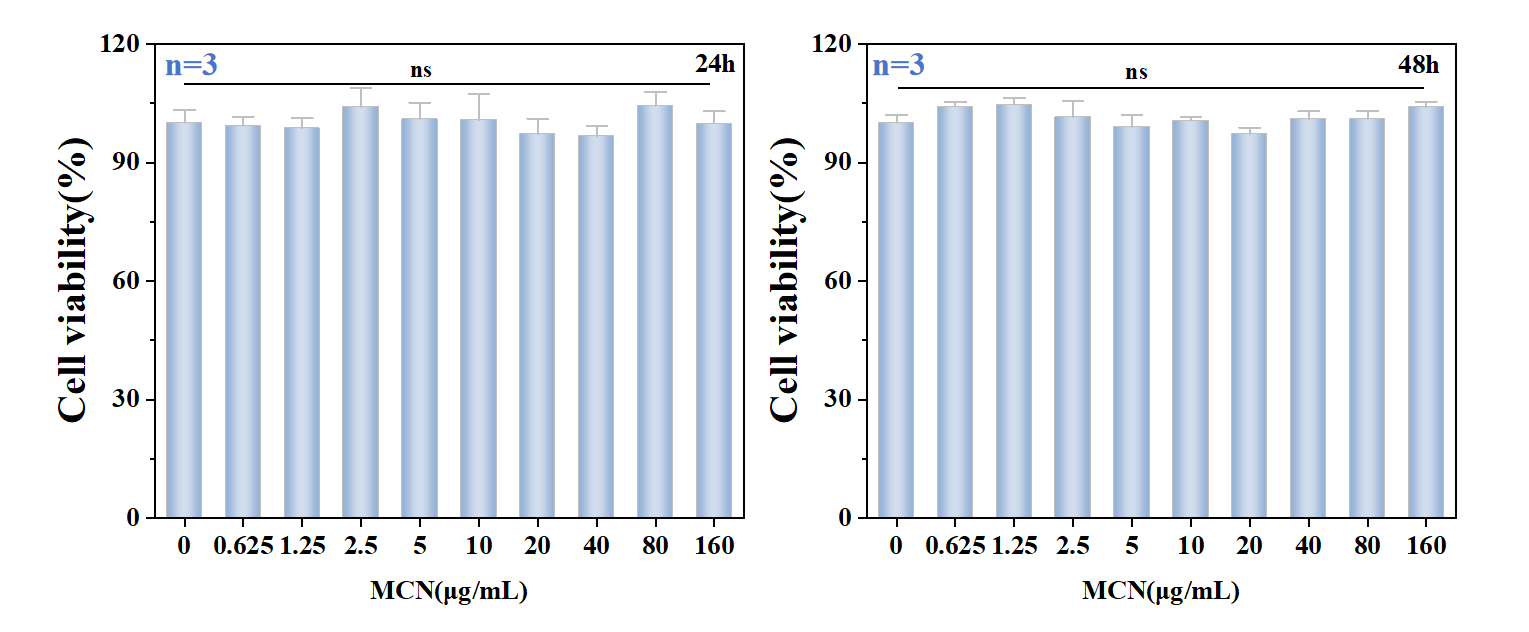


**Figure S57 CCK8 assay to detect the effect of different concentrations of MCN on cell viability after 24 h and 48 h treatment of BV2 cells.** Data were expressed as mean ± SE (in three independent experiments). Statistical significance was performed by one-way ANOVA with Tukey post hoc test. ns: *P* > 0.05, ^*^*P* < 0.05, ^**^*P* < 0.01, ^***^*P* < 0.001.


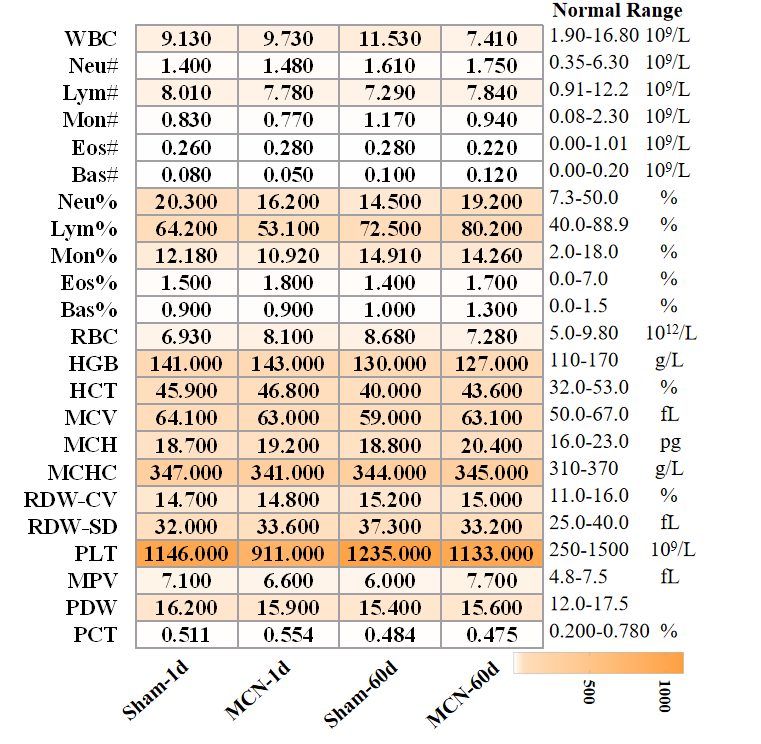


**Figure S58 Heatmap of Complete Blood Count for 1 d and 60 d.** Data were expressed as mean ± SE (in three independent experiments).


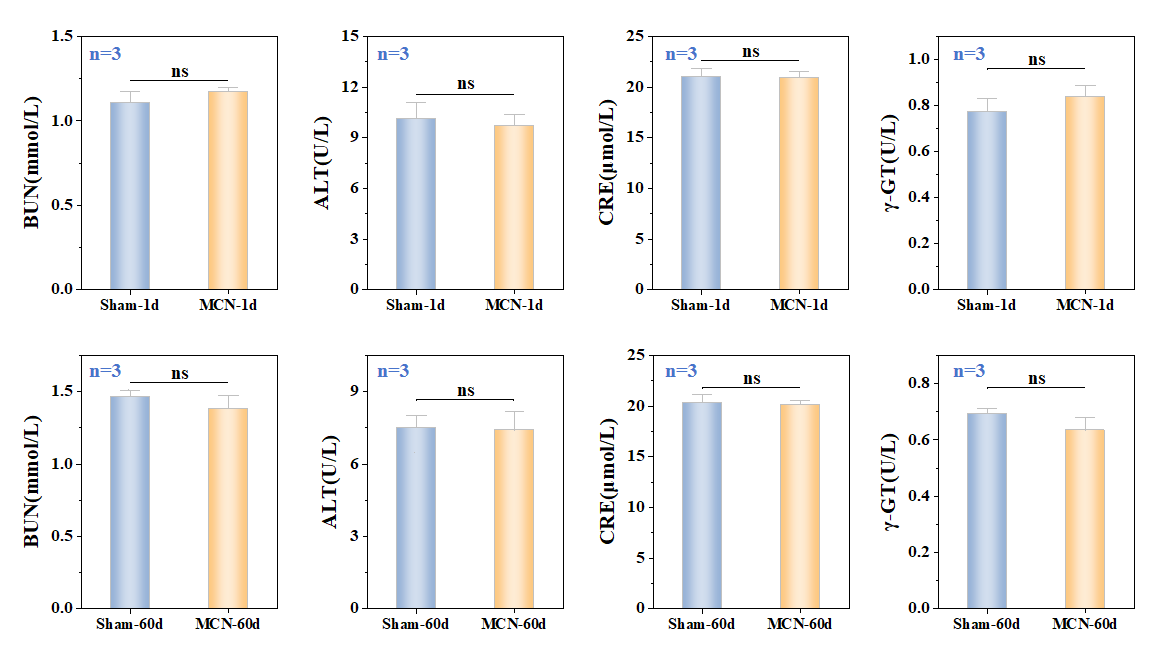


**Figure S59 Effects of MCN on kidney function in rats at 1 day and 60 days.** Data were expressed as mean ± SE (in three independent experiments). Statistical significance was performed by one-way ANOVA with Tukey post hoc test. ns: *P* > 0.05, ^*^*P* < 0.05, ^**^*P* < 0.01, ^***^*P* < 0.001.


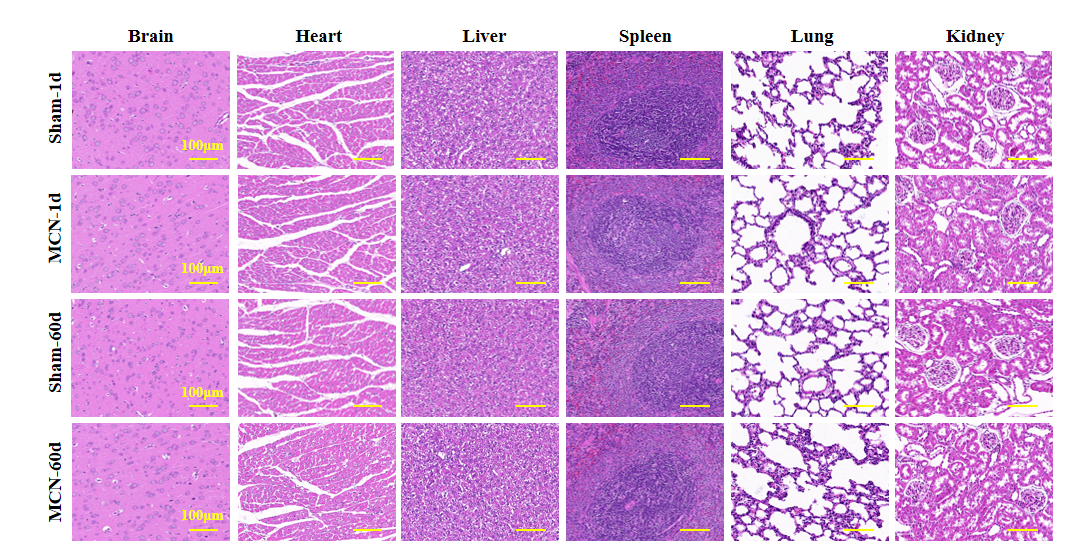


**Figure S60 Representative images of HE staining of major organs of rats for 1 d and 60 d.** Scale bar: 100 μm. Data were expressed as mean ± SE (in three independent experiments).
